# Dynamic Exchange Reactions as Self‐Blowing Agents for the Production of Reprocessable Foams

**DOI:** 10.1002/anie.202502970

**Published:** 2025-05-24

**Authors:** Antoine Adjaoud, Anaë Girault‐Fodil, Farida Baraka, Vincent Boulic, Benoit Marcolini, Laura Puchot, Pierre Verge

**Affiliations:** ^1^ Functional Polymers & Particulate Materials Luxembourg Institute of Science and Technology 5 Avenue des Hauts‐Fourneaux Esch‐sur‐Alzette L‐4362 Luxembourg; ^2^ Department of Physics and Materials Science University of Luxembourg 2 Avenue de l'Université Esch‐sur‐Alzette L‐4365 Luxembourg; ^3^ Engineering Faculty of Gipuzkoa, Chemical and Environmental Engineering Department, Biorefinery Processes Group University of the Basque Country Plaza Europa 1 Donostia 20018 Spain

**Keywords:** Benzoxazine vitrimers, Foam‐to‐resin reprocessing, Self‐blowing Foams, Sustainable Polymers, Transesterification exchange

## Abstract

Thermosetting foams are crucial in the polymer industry due to their unique lightweight and durable properties. Recent developments in sustainable chemistry intend to align foam lifecycles toward circular economy principles and reduce their environmental impact. In this study, this issue is addressed thanks to an innovative and straightforward approach, enabling the manufacture of single‐component self‐blowing vitrimer foam. The self‐blowing mechanism originates from the in situ formation and release of the blowing agent via the polycondensation of an alkylester. This alkylester, composed of a benzoxazine matrix terminated with a *β*‐hydroxylamine, undergoes in a cascade mechanism ring opening polymerization (ROP) and irreversible transesterification exchange, which in turn generates an alcohol gas. The cascade polymerization and alcohol release were optimized and fine‐tuned to develop various foams that have an open‐cell morphology, volume expansion of 223%–853%, porosity of 67%–86%, and compression modulus in the range of 3–38 MPa. The polymerization process results in a dual crosslinked poly(benzoxazine‐*co*‐ester) network composed of *β*‐hydroxylamine moieties, alkyl, and *β*‐aminoester bonds, enabling reversible transesterification dynamic exchanges and mechanical reprocessability, achieving a foam‐to‐resin reprocessing.

## Graphical abstract

This study introduces an innovative approach to designing self‐blown foams by leveraging the irreversible and self‐catalyzed transesterification of alkylester which release alcohol gas as blowing agents. The morphology and thermo‐mechanical properties of these foams can be precisely tailored by modifying the precursor structure. Remarkably, they can be reprocessed as rigid resins through reversible self‐catalyzed transesterification reactions.

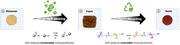

## Introduction

Thermosetting foams are lightweight and rigid porous structures fulfilling the specifications of various cutting‐edge industries.^[^ ^1^ ^]^ They are composed of a polymeric resin, a blowing agent (chemical or physical), and various additives, including catalysts or surfactants. The blowing agent, generally mixed with the precursors of the polymeric resin, generates a gaseous substance concomitantly to the resin cross‐linking, leading to the nucleation of empty cells while the polymeric material hardens irreversibly all around.^[^ ^2^ ^]^ Optimization of formulation and foaming conditions enables the control of the morphology and the thermo‐mechanical properties. Among the various commercially available foams, phenolic resins are ideal for developing foams with thermal and acoustic insulating properties.^[^ ^3^ ^]^ In this context, polybenzoxazine gained considerable interest in the development of lightweight and robust foams.^[^ ^4^, ^5^, ^6^, ^7^, ^8^ ^]^ Azodicarbonamide can be used as a chemical blowing agent, which releases nitrogen during the benzoxazine polymerization.^[^ ^9^ ^]^ Mixtures of methanol/chloroform were also considered as physical blowing solvent.^[^ ^10^ ^]^

The past decades witnessed the emergence of smart and sustainable strategies to improve the lifecycle of thermosetting foams. Particularly, the self‐blowing technique intends to reduce the complexity of the foaming process by avoiding the use of an external blowing agent.^[^ ^11^ ^]^ Self‐foaming resins offer significant advantages in terms of simplicity, durability, cost, and performance, making them potentially appealing for a wide range of industrial and environmental applications. The endogenous generation of the blowing agent comes from the thermolysis or the condensation of thermolabile groups embedded within the polymer network. In that respect, polyurethanes (PU) dominate the sector through the in situ release of carbon dioxide (CO_2_).^[^ ^11^ ^]^ While this self‐blowing mechanism involves the decomposition of unstable carbamic acids formed by the partial hydrolysis of isocyanate, the self‐foaming of nonisocyanate polyurethanes (NIPUs) has also focused attention. For instance, Monie et al. used the aminolysis and the chemoselective addition of thiol onto cyclic carbonates to produce CO_2_ as a self‐blowing agent.^[^ ^12^ ^]^ Similar approaches using CO_2_ alone or in combination with another gas have also been employed for epoxy resins,^[^ ^13^ ^]^ poly(arylene ether ketone),^[^ ^14^ ^]^ or polycarbonate,^[^ ^15^ ^]^ to cite but a few. In the case of polybenzoxazine, Cadiz and coworkers reported the design of self‐blowing foams from a diphenolic acid (DPA)‐based benzoxazine precursor.^[^ ^16^, ^17^ ^]^ CO_2_ was produced from the thermally induced decarboxylation of DPA, leading to foams with superior thermal and mechanical properties thanks to the highly cross‐linked and aromatic structure of polybenzoxazine. In all cases, the kinetics of the release of the foaming agent plays a critical role and must be closely aligned with the polymerization kinetics of the resin to ensure the formation of a foam with optimal properties. If the gas is released too early, before the resin has sufficiently polymerized, the bubbles may escape or collapse. If the gas is released too late, the resin may already be too viscous or solidified, preventing effective and uniform expansion.

Like thermosetting resins, structural foams have limited end‐of‐life options. The incorporation of dynamic covalent bonds within their structure is a way to address this challenge. For instance, self‐blown NIPU foams obtained from the decarboxylative *S*‐alkylation pathway contain reversible hydroxyurethane bonds that can undergo reversible exchanges, either through aminolysis or transcarbamoylation.^[^ ^18^, ^19^, ^20^, ^21^ ^]^ Switching from a polyamine to a hydroxyl‐terminated polycaprolactone, Sardon and coworkers also developed self‐blown foams that can be reprocessed through transcarbonation.^[^ ^15^ ^]^ Du Prez and coworkers introduced thermoreversible triazolinedione (TAD)‐indole or *β*‐aminoester linkages into the design of polyurethane foams. Depending on the type of dynamic exchange, the materials can be reprocessed as elastomers^[^ ^22^ ^]^ or recycled as foams,^[^ ^23^ ^]^ respectively. Finally, Tian et al. reported the design of reprocessable foams based on the transesterification of *β*‐hydroxyester bonds internally catalyzed by tertiary amine groups.^[^ ^24^ ^]^ In all these systems, the endogenous chemical blowing is independent of the dynamic exchange process. Typically, dynamic exchange is employed for recycling, while a separate chemical reaction is responsible for gas formation, allowing for the foaming process. However, it could be envisioned that the dynamic exchange mechanism itself could serve a dual purpose: generating the gas required for foaming and imparting vitrimer properties to the material. Indeed, previous works reported the design of self‐blown foams focusing on thermolabile ester groups relied on the thermolysis of *tert*‐butyl ester^[^ ^25^ ^]^ or the carboxylic acid‐alcohols condensation;^[^ ^26^ ^]^ releasing gaseous isobutene or water as internal self‐blowing agents, respectively. These strategies suggest that the self‐blowing agents can be finely tuned to optimize foam properties.

It is noteworthy that despite the extensive research both on self‐blowing materials and covalent adaptable networks, the combination of polymerization kinetics, dynamic exchanges, and self‐foaming within a single material has not been explored yet. One could envision a scenario where a transesterification reaction (TER) is designed such that, upon thermally triggered exchange, an alcohol gas is released. If this irreversible process occurs concurrently with polymerization during the nucleation stage, it could result in a self‐foaming mechanism. Interestingly, in benzoxazine‐based vitrimers relying on TER self‐catalyzed by in situ generated tertiary amine groups, these phenomena occur simultaneously. Indeed, the benzoxazine ring‐opening polymerization (ROP) triggers both polymerization and dynamic exchange reactions.^[^ ^27^ ^]^ This unique interplay could enable the development of self‐blowing vitrimers with relative ease. More importantly, this approach could potentially address issues of asynchrony, often encountered during foaming processes. Since these reactions occur at the temperature range of the glass transition (*T*_g_) and the topology freezing temperature (*T*_v_), the nucleation phase can benefit from the dynamic exchanges that could help relax the internal stress caused by gas volume expansion on the cell walls.

**Figure 1:**
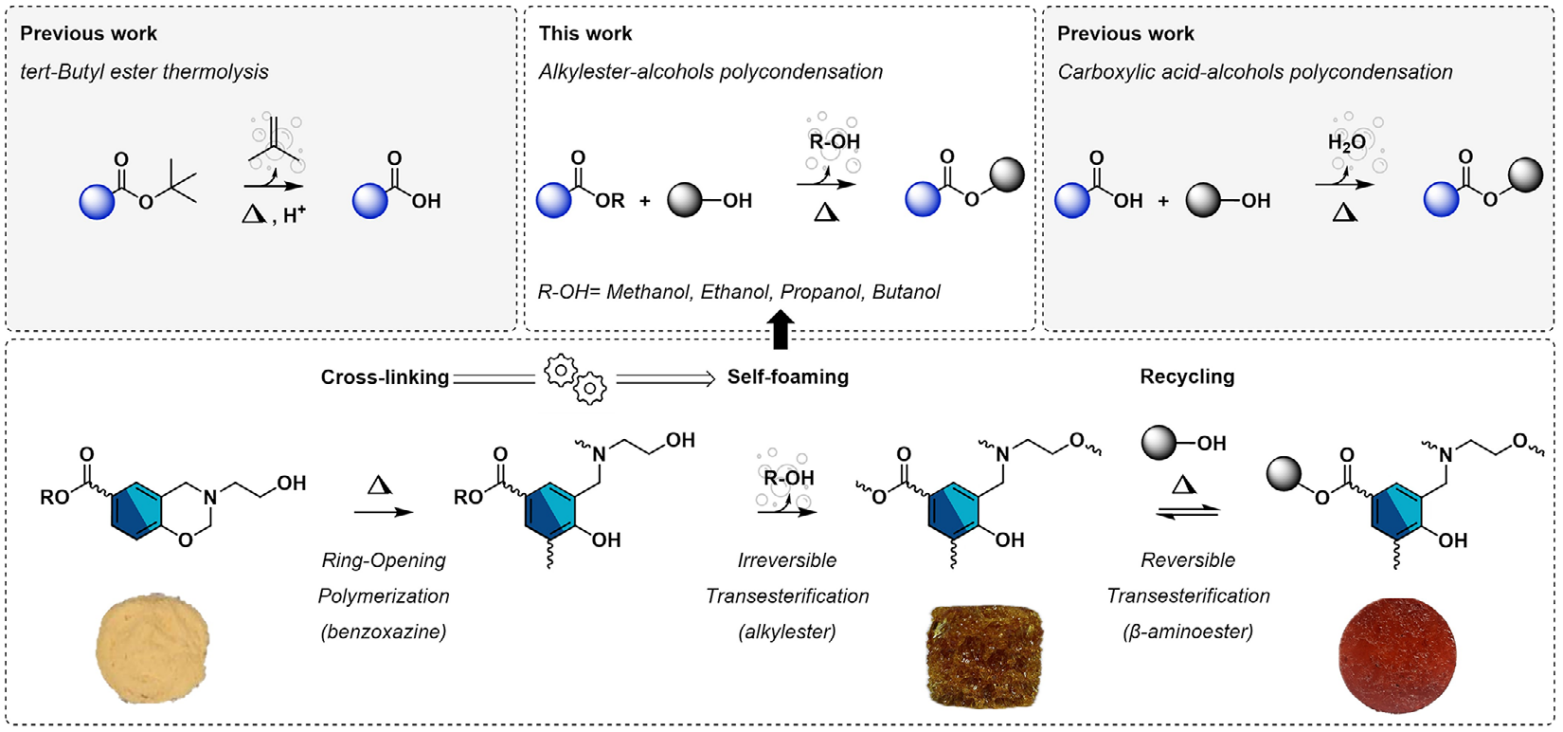
General foaming and mechanical reprocessing of self‐blowing polybenzoxazine foams.

This new concept is exploited in this study to develop single‐component, self‐blowing, and reprocessable foams (Figure 1). Their formation is monitored by the opening of benzoxazine rings upon exposure to heat, which triggers cascade polymerization of the benzoxazine rings, along with the polycondensation between alkylester and hydroxyl groups. The two reactions lead to the formation of a poly(benzoxazine‐*co*‐ester) dual cross‐linked network, along with the release of an alcohol in its gaseous state coming from the alkylester polycondensation, generating a cellular structure. A series of benzoxazine precursors prepared from *β*‐aminoalcohol and alkylester moieties (from CH_3_‐ to CH_3_(CH_2_)_3_‐ester) have been synthesized in a two‐step chemical pathway. Several parameters were investigated, including the nature of the alkylester—and consequently the type of alcohol released—the benzoxazine functionality, and the polymerization kinetics. This enabled the production of foams ranging from brittle to resilient, with diverse cellular structures. Finally, a foam‐to‐resin reprocessability approach is presented.

## Results and Discussion

### Alkylester‐Based Benzoxazine Precursors

**Scheme 1:**
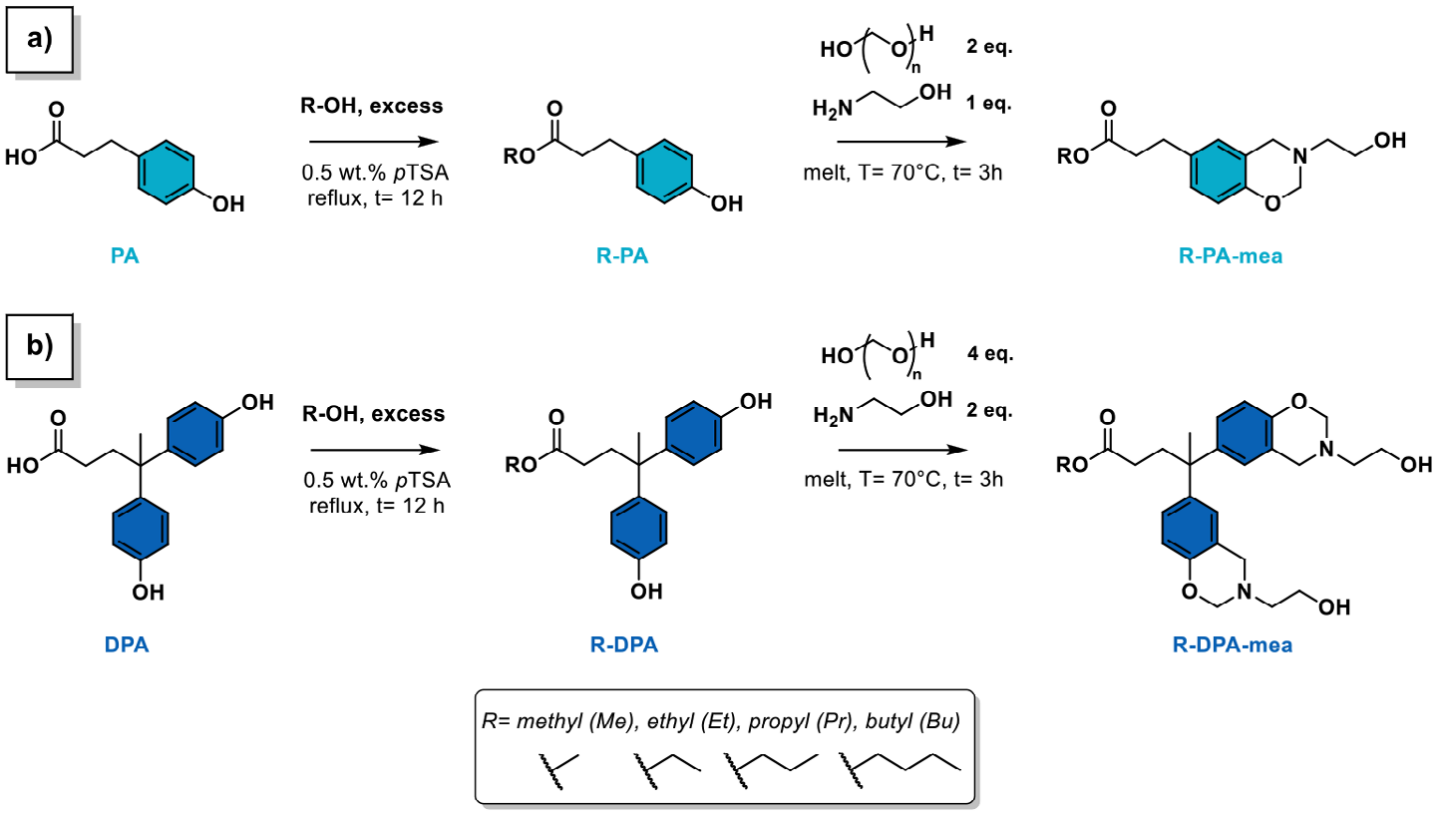
Two‐step synthetic pathway for the design of alkylester‐based benzoxazine precursors.

*Synthesis*. The synthesis of alkylester‐based benzoxazine precursors follows a two‐step chemical pathway reported in Scheme 1. The first step corresponds to a Fischer esterification of a mono‐ or difunctional phenolic acid (phloretic acid = PA or diphenolic acid = DPA in Scheme 1a,b, respectively) with a mono‐alcohol. The length of the alkylester side chain varied from one to four carbons depending on the alcohol, including methanol (Me), ethanol (Et), propanol (Pr), and butanol (Bu) (Figures S1–S8). The second step involves the Mannich‐like condensation using paraformaldehyde and mono‐ethanolamine (mea) as aldehyde and amine sources, respectively. The process yields benzoxazine monomers terminated with a linear alkylester and with one or two benzoxazine rings end‐capped with a *β*‐aminoalcohol moieties on the other side of the molecule. The resulting molecules are designated as R‐PA‐mea or R‐DPA‐mea, depending on the phenolic acid and mono‐alcohol used. It is noteworthy that R‐DPA‐mea is a difunctional molecule, whereas R‐PA‐mea is monofunctional.

**Figure 2:**
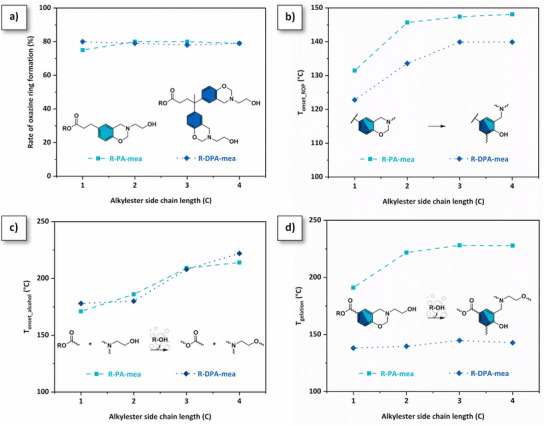
a) Rate of oxazine rings as a function of the length of the alkylester side chain (^1^H NMR spectroscopy, integration of N─CH_2_*─O peak). Monitoring of cross‐linking reactions in R‐PA‐mea and R‐DPA‐mea alkylester‐based benzoxazine precursors as a function of the length of alkylester side chain. b) Onset temperature of benzoxazine ring‐opening reaction (DSC experiment, heating rate 10 °C·min^−1^). c) Onset temperature detection of alcohol self‐blowing agent generated through irreversible TER (TGA‐µGC experiment, heating rate 2 °C·min^−1^). d) Gelation temperature (rheological measurement, heating rate 2 °C·min^−1^).

The chemical structure of the foam precursors was substantiated by ^1^H and ^13^C NMR spectroscopy (Figures S9–S16) and elemental analysis (Table S3). The formation of the aliphatic ester bond is confirmed on the ^13^C spectrum of each precursor by the characteristic peak at *δ* = 173–174 ppm. The vanishment of the phenol signal (*δ* = 9.2 ppm), along with the emergence of methylene protons bound to heteroatoms in six‐membered oxazine ring (*δ* = 3.9 and *δ* = 4.8 ppm for Ar─CH_2_*─N and N─CH_2_*─O, respectively), is indicative of the success of the Mannich‐like condensation. The amount of closed benzoxazine rings in the precursor reaches the equilibrium state of 80% independently of the functionality of the precursors or the length of the alkylester side chain (Figure 2a). The other adduct of the Mannich‐like condensation corresponds to a five‐membered oxazolidine ring originating from a tautomeric equilibrium between the neutral and zwitterionic forms of oxazine rings end‐capped with *β*‐aminoalcohol moieties, recurrently observed in such precursors.^[^ ^27^, ^28^ ^]^

*Monitoring of cross‐linking reactions*. The thermally activated ring‐opening polymerization (ROP) of benzoxazine monomers was monitored by differential scanning calorimetry (DSC). The DSC thermograms of R‐PA‐mea and R‐DPA‐mea monomers are displayed in Figures S17 and S18, respectively. The first exothermic peak detected in the range of 120–200 °C corresponds to the oxazine ring‐opening reactions. The accelerated ROP originates from the neighboring group participation (NGP) of *β*‐aminoalcohol moieties and the formation of reactive oxazolidine‐like substructures.^[^ ^27^ ^]^ The precursors’ functionality as well as the length of the alkylester side chain play a significant role in the ROP. As shown in Figure 2b, the onset temperature of benzoxazine ring‐opening reaction (*T*_onset_) is primarily governed by the functionality of the monomer, followed by the concentration of reactive species. Notably, each difunctional‐based precursor shows a lower *T*_onset_ compared to their monofunctional counterparts due to the higher concentration of reactive *β*‐aminoalcohol moieties. This concentration is also influenced by the length of the alkylester side chain, which explains why *T*_onset_ increases with longer side chains. The second exothermic peak detected in the range of 225–250 °C corresponds to thermal degradation of the monomers.

The heat‐induced polymerization of both R‐PA‐mea and R‐DPA‐mea benzoxazine foam precursors is a cascade mechanism. The benzoxazine ring‐opening generates tertiary amines that catalyze the transesterification exchanges between ester bonds and *β*‐aminoalcohol moieties.^[^ ^27^ ^]^ The condensation of alkylester bonds over *β*‐aminoalcohol moieties contributes to forming a polyester network (Figure 2c). The process is irreversible, as mono‐alcohol species evaporate during the curing process, as observed by thermogravimetric analysis (TGA) coupled with micro‐computed gas chromatography experiment (µGC). Figures S19–S26 complete this observation, demonstrating the release of alcohol (methanol, ethanol, propanol, and butanol) along with the mass loss of each precursor as a function of temperature. Figure 2c highlights that the release of mono‐alcohol solvents can be detected for all benzoxazine foam precursors.

The higher temperatures of detection of alcohol solvents (*T*_onset___alcohol_) measured with increasing alkylester side‐chain length suggest lower capability to evaporate (*T*_onset___alcohol_ = 170–220 °C from methanol‐to‐butanol‐based precursors). The occurrence of irreversible TER is also confirmed by following the complex viscosity of each precursor through rheokinetic measurements using temperature sweep curves (Figures S27 and S28) or isothermal (Figures S29 and S30) experiments. R‐DPA‐mea precursors rapidly form a network due to the presence of two benzoxazine groups per molecule (*T*_gel _= 138–145 °C). Interestingly, R‐PA‐mea also crosslinks, despite being monofunctional. This is notable, as monofunctional benzoxazines are typically known to oligomerize only up to a molecular weight of several thousand g·mol^‐^^1^.^[^ ^29^ ^]^ In these cases, the gelation is only explainable by the polycondensation of alkylester with aliphatic ─OH groups. This is also confirmed by the absence of gelification in a furfurylamine‐based analog,^[^ ^27^ ^]^ as it is not able to undergo esterification due to the absence of aliphatic ─OH groups. The effect of the alkylester side chain is similar for both R‐PA‐mea and R‐DPA‐mea. In both cases, the gelation temperature (*T*_gel_) and time (*t*_gel_) increase with the number of carbon atoms in the side chain (Figure S31). However, one exception is observed: the *T*_gel_ of R‐DPA‐mea remains similar, indicating that these precursors’ functionality primarily governs their gelation mechanism (Figure 2d). Independently of their functionality, the length of the alkylester side chain also plays a significant role in the viscosity of the precursors (Figures S32 and S33). While previous work demonstrated that the opening of benzoxazine rings was responsible for triggering TER within these precursors,^[^ ^27^ ^]^ these new results highlight the release of alcohol in a cascade mechanism. Interestingly, under nonisothermal curing conditions, the increase in complex viscosity begins earlier than the detection of alcohol traces by TGA‐µGC. In fact, the measurements clearly show that alcohol release becomes detectable at a temperature above the sol‐gel transition. One explanation is that benzoxazine rings crosslink faster at lower temperatures as compared to the kinetics of TER. At low crosslinking extents, the material viscosity remains sufficiently low to allow dynamic exchanges, enabling reversible and irreversible TER of alkylester. In fact, while viscosity gradually increases with elevated temperature, the kinetics of dynamic TER decreases, and conditions become more favorable for alcohol release. With precise fine‐tuning of the curing conditions, this mechanism could support a controlled foaming process, making it both feasible and further optimizable.

### Self‐Blown Polybenzoxazine Foams

*Optimization of the foaming process*. The release of an alcohol gas during the curing of alkylester‐based benzoxazine was used as chemical self‐blowing process to produce foams. The weight loss occurring during foaming was directly correlated to the extent rate of irreversible TER (*Y*_TER_), calculated according to Equation S2. It is noteworthy that benzoxazine curing releases very few, if any, by‐products,^[^ ^4^ ^]^ and the thermal degradation of the precursors is not observed below 225 °C (Figures S17 and S18). In other words, the mass loss observed by TGA at this temperature can be directly attributed to the release of the alcohol blowing agent. The volumetric expansion (*V*_exp_) was calculated by dividing the volume of the structural cylinder foam by the initial volume of the precursor, as described in Equation S3.

**Figure 3:**
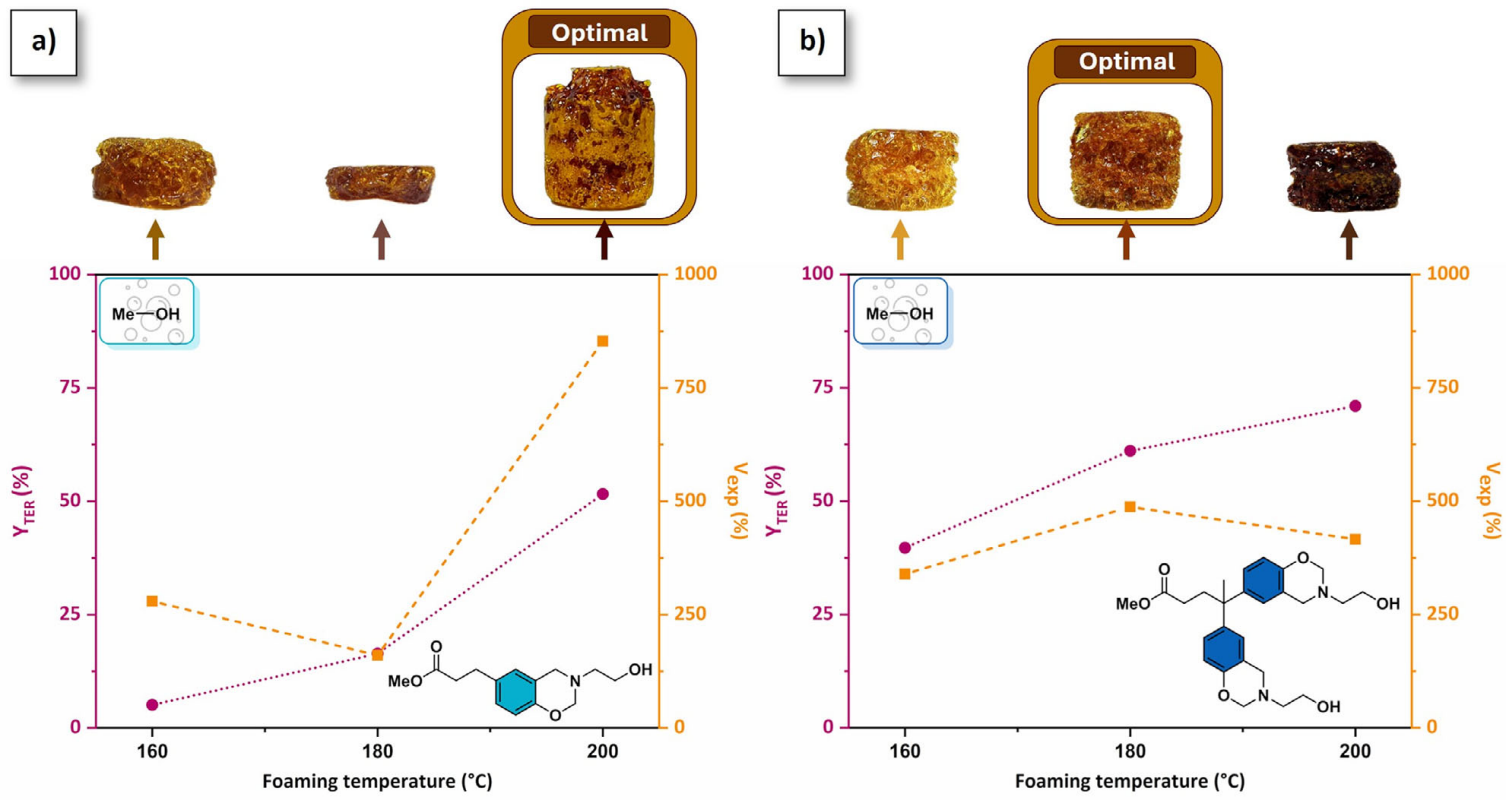
Optimization of the foaming process as a function of the foaming temperature: evolution of the extent rate of irreversible transesterification and volumetric expansion as a function of a) Me‐PA‐mea and b) Me‐DPA‐mea.

Foaming conditions were optimized by adjusting the temperature and duration for methanol‐based precursor curing. While minimal foaming of Me‐PA‐mea precursors was observed at temperatures below 180 °C, *p*(Me‐PA‐mea) foam obtained at 200 °C demonstrates the higher rate of irreversible transesterification and volumetric expansion (*Y*_TER_ = 52%, *V*_exp_ = 853%, Figure 3a). For *p*(Me‐DPA‐mea), *Y*_TER_ increases from 40% to 71% with the temperature increase (Figure 3b), but *V*_TER_ does not follow the same trend and a maximum of 478% of volume expansion is observed at 180 °C. At this temperature, the foaming process is completed within 20 min (Figure S34). Based on this preliminary study, the optimal foaming conditions were selected as a trade‐off between dimensional stability, volumetric expansion, and extent rate of irreversible TER. A heating ramp from room temperature to 130 °C was systematically applied for R‐DPA‐mea precursors to ensure complete melting of the monomer. Following an equilibrium phase at 130 °C over 10 min, the temperature was elevated to induce cascade ring‐opening polymerization of benzoxazine rings and irreversible TER of alkylester (heating rate of 5 °C·min^−1^). The final foaming temperature was maintained for a period of 20 min. The foaming temperature was increased to 220 °C for *p*(Et‐PA‐mea), *p*(Pr‐PA‐mea), and *p*(Bu‐PA‐mea) to maximize the rate of irreversible TER. The optimal foaming temperature was set at 180 °C for *p*(Et‐DPA‐mea) and at 200 °C for *p*(Pr‐DPA‐mea) and *p*(Bu‐DPA‐mea). The absence of an exothermic peak on the DSC thermograms of the self‐blown polybenzoxazine foams indicates complete ring‐opening of benzoxazine moieties (Figures S35 and S36). The brown coloration of the self‐blown polybenzoxazine foams primarily arises from their intrinsic aromatic structure and the presence of substituted phenolic groups.

**Figure 4:**
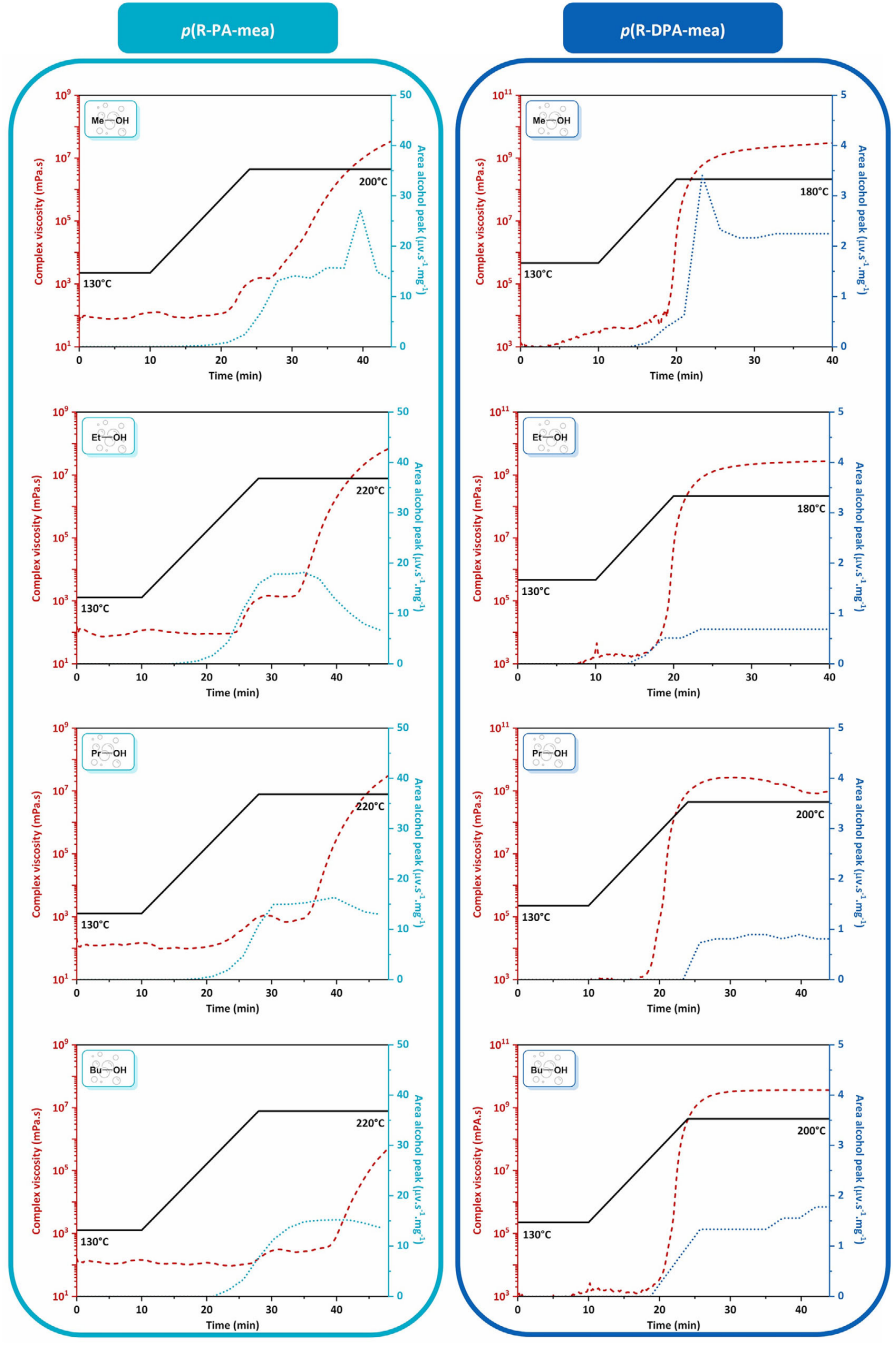
Monitoring of the complex viscosity (η*, *γ* = 1%, *ω* = 1 s^−1^) and the release of self‐blowing agent (*A*_alcohol_) as a function of the optimized foaming process.

Efficient cell nucleation and foam growth require balancing the viscosity of the precursors with the degree of crosslinking and the generation of self‐blowing agent. Figure 4 gathers the evolution of the complex viscosity (η*) and the release of the alcohol (*A*_alcohol_) formed from each alkylester‐based benzoxazine precursor. For all precursors, the complex viscosity remains constant during the isothermal step at 130 °C. Upon temperature increase, the rise in complex viscosity originating from cross‐linking is concomitant to the release of an alcohol solvent. These experiments suggest that the foaming begins at the boundary of the viscoelastic liquid and sol‐gel rubbery zones, or even within the sol/gel rubbery zone, and it continues until the material reaches the rubbery or vitreous gel zones. Within this viscosity window, the material possesses sufficient fluidity to trap gas bubbles and allow their nucleation and growth. The increasing crosslink density prevents the collapse of the bubbles, ultimately providing the structural stability needed to maintain the foam in the vitreous state. The foaming process was optimized for each precursor by carefully balancing viscosity during crosslinking with the activation temperature of the self‐blowing agents.

**Figure 5:**
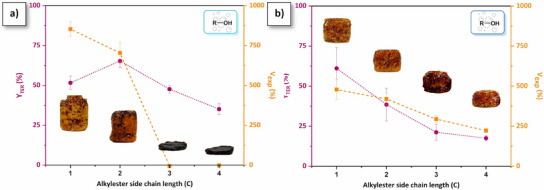
Evolution of the extent rate of irreversible transesterification and volumetric expansion of a) *p*(R‐PA‐mea) and b) *p*(R‐DPA‐mea) as a function of the length of the alkylester side‐chain.

The extent rate of irreversible TER (*Y*_TER_) and the volumetric expansion (*V*_exp_) of the foams are reported in Table 1 and Figure 5. It is a reproducible process (Figure S37) applicable to different container volumes and shapes (Figure S38). In *p*(R‐PA‐mea), the *Y*_TER_ values are relatively similar, ranging between 35% and 65%. The expansion volume reaches 850% for *p*(Me‐PA‐mea) and 700% for *p*(Et‐PA‐mea). However, *p*(Pr‐PA‐mea) and *p*(Bu‐PA‐mea) do not foam. This is due to the fact that the alcohol is released while the material remains in the liquid phase, preventing it from being trapped within the structure of the forming gel. By comparison, *V*_exp_ for difunctional precursors falls within the 250%–500% range, decreasing linearly with both the length of the alkylester side chain and the *Y*_TER_ values. In these cases, the gas release occurs in the rubbery sol‐gel phase. The reduced foaming observed with increasing alkyl ester chain lengths can be attributed to the lower volatility of the resulting alcohols at the curing temperature. However, as the alkylester chain length influences both reactivity and the cross‐linking density of resulting materials, drawing clear conclusions remains challenging. The foaming parameter (*η*_foaming_, Table 1, column 5), corresponding to the relative amount of volume expansion observed as compared to the volume of alcohol vapor generated, varies from 4.6% to 13.5% (Equation S4, details of calculation in the Supporting Information). These values indicate that a minimal quantity of self‐blowing agent is required to form a foam cellular structure.

**Table 1:** Characteristics of self‐blown foams.

Foam	Final foaming temperature (°C)	*Y*_TER_ (%)^a)^	*V*_exp_ (%)^b)^	*η*_foaming_ (%)^c)^
*p*(Me‐PA‐mea)	200	52 ± 4	853 ± 46	13.5
*p*(Et‐PA‐mea)	220	65 ± 4	704 ± 67	9.5
*p*(Pr‐PA‐mea)	220	48 ± 3	−6 ± 12	n.a.
*p*(Bu‐PA‐mea)	220	35 ± 3	−2 ± 10	n.a.
*p*(Me‐DPA‐mea)	180^d)^	61 ± 13	478 ± 62	11.8
*p*(Et‐DPA‐mea)	180^d)^	39 ± 10	420 ± 36	6.8
*p*(Pr‐DPA‐mea)	200^d)^	21 ± 5	294 ± 21	5.5
*p*(Bu‐DPA‐mea)	200^d)^	18 ± 2	223 ± 14	4.6

^a)^ Extent rate of irreversible transesterification calculated according to Equation S2.

^b)^ Volumetric expansion calculated according to Equation S3.

^c)^ Foaming parameter calculated according to Equation S4.

^d)^ Initial heating ramp from room temperature to 130 °C.

**Figure 6:**
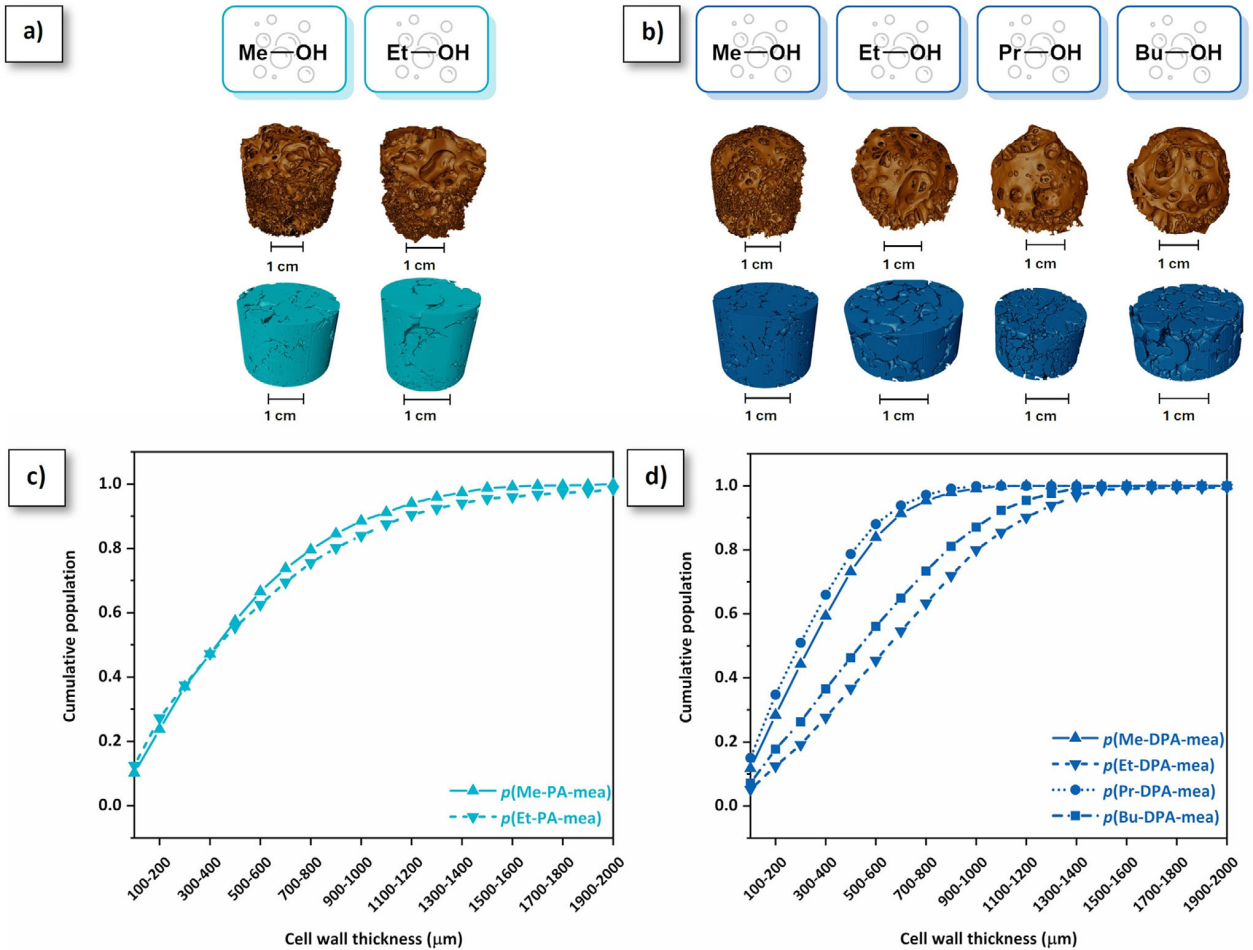
3D tomography images of a) *p*(R‐PA‐mea) and b) *p*(R‐DPA‐mea). Distribution of cell wall thickness of c) *p*(R‐PA‐mea) and d) *p*(R‐DPA‐mea).

*Microstructure and thermo‐mechanical properties*. The microstructure of the polybenzoxazine foams was analyzed by micro‐computed X‐ray tomography (µCT). 3D tomography images of *p*(R‐PA‐mea) and *p*(R‐DPA‐mea) are shown in Figure 6a,b, respectively. The 3D reconstruction reveals an open‐cell morphology with interconnected cells. Although the median pore size was challenging to determine due to the open‐cell interconnected structure, the cell wall thickness was assessed in the condensed phase (Figure 6c,d). It ranged from less than 100 to over 2000 µm, with a distribution influenced by the structural characteristics of the precursors and the foaming process. Processes and precursors with high *Y*_TER_ promoted the cell merging and rupture, resulting in a reduction of cell wall thickness. At similar foaming temperatures (180 °C for *p*(Me‐DPA‐mea) and *p*(Et‐DPA‐mea) and 200 °C for *p*(Pr‐DPA‐mea) and *p*(Bu‐DPA‐mea)), the thinner distribution of pore cell wall was obtained for shorter alcohol (Me vs. Et and Pr vs. Bu). Additionally, Figure S39 illustrates the significant impact of the foaming temperature on the distribution of cell wall's thickness of *p*(Me‐DPA‐mea).

Table 2 summarizes the morpho‐structural and thermo‐mechanical properties of the self‐blown polybenzoxazine foams. The gradual decrease in the porosity (*e*) of the resulting foams follows a similar trend, decreasing from *e* = 85.9% for *p*(Me‐DPA‐mea) to *e* = 66.7% for *p*(Pr‐DPA‐mea), except for *p*(Bu‐DPA‐mea) that could be attributed to the restricted gas diffusion due to its size, resulting in the formation of thicker cell's wall (Figure 6b). The bulk density (*ρ*) tends to increase with the carbon chain length, from 240 to 335 kg·m^−3^ from *p*(Me‐DPA‐mea) to *p*(Bu‐DPA‐mea), as a consequence of the decreased extent rate of irreversible TER (Figure 5b). For the same self‐blowing agent (methanol), the lower bulk density observed for *p*(Me‐PA‐mea) compared to *p*(Me‐DPA‐mea) (161 and 240 kg·m⁻^3^, respectively) can be attributed to the higher concentration of the self‐blowing agent in the monofunctional precursor.

**Table 2:** Morpho‐structural and thermo‐mechanical properties of the self‐blown polybenzoxazine foams.

Foam	*e* (%)^a)^	*ρ* (kg.m^−3^)^b)^	*ε*_break_ (%)^c)^	*σ*_strength_ (kPa)^d)^	*E*_compression_ (MPa)^e)^	*T*_d5%_ (°C)^f)^	CR_800 °C_ (%)^g)^
*p*(Me‐PA‐mea)	80.8	161 ± 6	0.8 ± 0.1	20 ± 9	2.8 ± 1.3	217	35.8
*p*(Et‐PA‐mea)	82.0	182 ± 12	0.7 ± 0.2	23 ± 9	2.8 ± 1.7	234	36.8
*p*(Me‐DPA‐mea)	85.9	240 ± 26	1.6 ± 0.2	578 ± 88	35 ± 9	239	24.8
*p*(Et‐DPA‐mea)	71.9	241 ± 28	1.4 ± 0.3	518 ± 176	38 ± 4	235	25.3
*p*(Pr‐DPA‐mea)	66.7	285 ± 26	2.4 ± 0.2	662 ± 342	20 ± 5	240	25.7
*p*(Bu‐DPA‐mea)	74.8	335 ± 16	2.6 ± 0.3	655 ± 190	21 ± 5	244	27.1

^a)^ Average porosity calculated by µCT.

^b)^ Average bulk density.

^c)^ Compression stress at break.

^d)^ Compression strength (compression strain at break).

^e)^ Young modulus (compression testing, 2 mm·min^−1^).

^f)^ Temperature of 5 wt.% thermal degradation (N_2_ atmosphere).

^g)^ Char yield at 800 °C.

The mechanical properties of *p*(R‐PA‐mea) and *p*(R‐DPA‐mea) were evaluated by uniaxial compression tests (Figures S40 and S41). The compressive profile is characteristic of rigid foams with three different regimes: the elastic deformation (initial linear slope at strain deformation ranging from 0% to 1%), the crushing domain (collapse of cell walls), and finally the densification (drastic increase of the compressive stress).^[^ ^30^ ^]^ While *p*(R‐PA‐mea) foams exhibit the higher rate of irreversible TER, these foams are more friable (*E*_compression_ < 3 MPa) due to their lower crosslinking density. Foams obtained from difunctional precursors are characterized by higher mechanical properties compared to their monofunctional counterparts (*σ*_strength_ = 500–700 kPa, *E*_compression_ = 20–38 MPa). For similar final foaming temperatures (i.e., 180 °C for *p*(Me‐DPA‐mea) and *p*(Et‐DPA‐mea), and 200 °C *p*(Pr‐DPA‐mea) and *p*(Bu‐DPA‐mea)), foams with higher bulk density exhibit higher compression modulus. The difunctionality also allows for higher thermal stability (Figures S42 and S43), as of *T*_d5%_ > 235 °C compared to *T*_d5%_ > 217 °C for the monofunctional precursor.

### Foam‐to‐Resin Reprocessing

**Figure 7:**
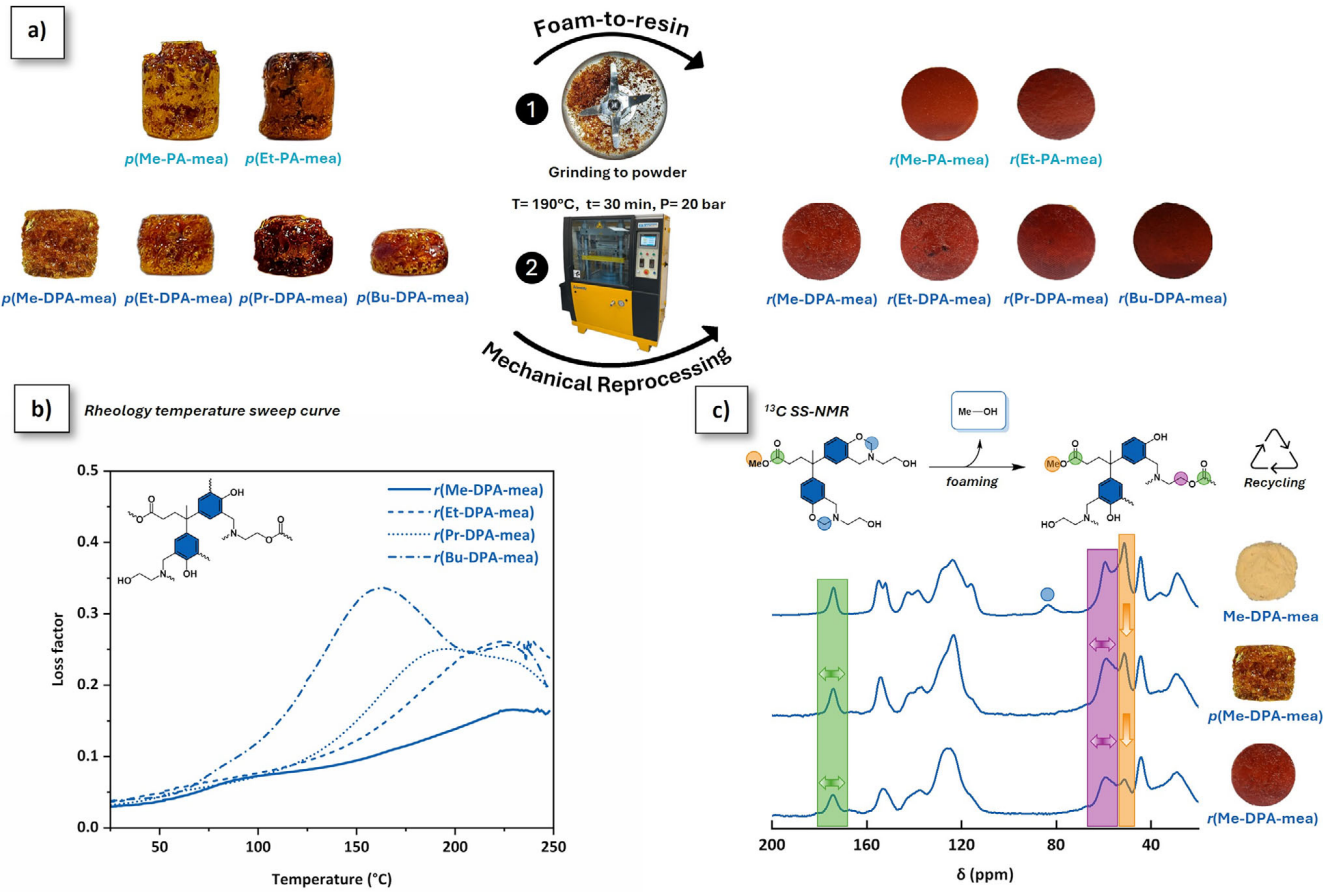
a) Photographs of mechanical reprocessing of self‐blown polybenzoxazine foams by thermo‐reprocessing. b) Evolution of the loss factor *r*(DPA‐mea) determined by rheology temperature sweep curve experiment (*f* = 1 Hz, 2 °C·min^−1^). c) ^13^C NMR spectrum of Me‐DPA‐mea, *p*(Me‐DPA‐mea), and *r*(Me‐DPA‐mea).

**Scheme 2:**
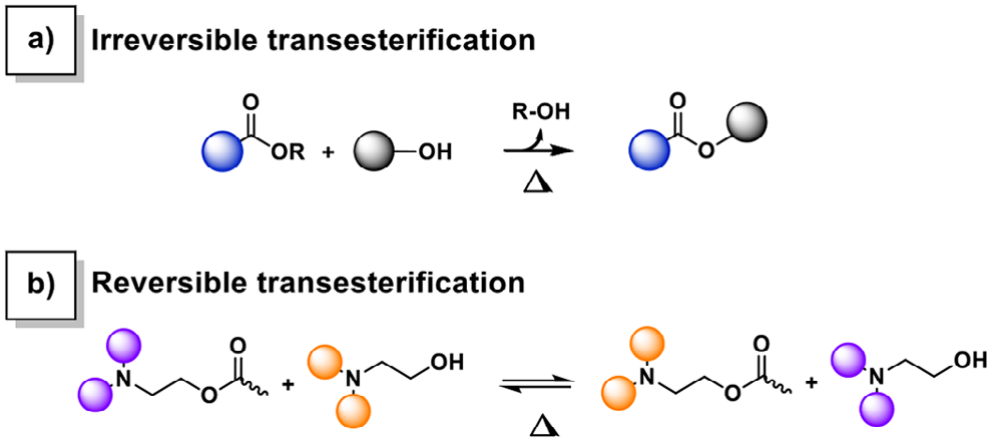
Possible a) irreversible and b) reversible TER involved in the thermo‐reprocessing of self‐blown polybenzoxazine foams.

Dynamic covalent bonds are known to promote recyclability into crosslinked networks.^[^ ^31^ ^]^ The foam‐to‐resin mechanical reprocessing of the foams is shown in Figure 7a. Translucent homogeneous reprocessed resins (*r*(R‐PA‐mea) and *r*(R‐DPA‐mea)) were obtained by hot pressing of crushed foam at 190 °C. The mechanical reprocessing is reproducible for all self‐blown polybenzoxazine foams (Figure S44) and extended to four cycles for *r*(Me‐DPA‐mea) (Figure S45). R‐PA‐mea precursors have a stoichiometric amount of dynamic aliphatic ─OH and ester bonds. After curing, the irreversible TER would theoretically result in a structure devoid of aliphatic ─OH groups, which are essential for the dynamic exchanges to occur. However, this step is not quantitative (Figure 5), and unreacted alkylester and *β*‐aminoester adducts can still be found, which explains why mechanical reprocessing remains possible as shown in Scheme 2.

As illustrated in Figure 7a, the reprocessed resins present a homogenous distribution of condensed phase with minimal porosity, highlighting the efficiency of the mechanical reprocessing (Table 3, column 2). The average bulk density of the resulting reprocessed resin approximates the density of the monomer (Tables 3, column 3, and Table S1, column 2). The mechanical properties of the reprocessed resins were analyzed by torsional shear testing (shear strain–stress curves, Figures S47 and S48; Table 3, columns 4–6). The shear modulus is in the range of 1 GPa. The *α*‐mechanical relaxations (*T*_α_) were measured by torsional rheology temperature sweep experiments (Figures 7b and S49–S51). They are between 144 and 114 °C for *r*(R‐PA‐mea), depending on the length of the alkylester side chain, and 232 and 164 °C for *r*(R‐DPA‐mea). The molecular weight between cross‐linking nodes (Mc) was quantitatively determined from the shear storage modulus (*G*’) in the rubbery plateau region (Table S4). For the reprocessed *r*(R‐DPA‐mea) resins, Mc increased progressively from 50 to 160 g·mol⁻¹ with the length of the alkylester side chain. These values—slightly higher than those typically observed for conventional polybenzoxazines—are consistent with the formation of a dual cross‐linked poly(benzoxazine‐co‐ester) network. The slightly higher *T*_d5%_ observed for the reprocessed materials is attributed to additional irreversible TER occurring during the reprocessing step (Table 3, columns 7 and 8; Figures S52 and S53).

**Table 3:** Morpho‐structural and thermo‐mechanical properties of the reprocessed polybenzoxazine resins.

Resin	*e* (%)^a)^	*ρ* (kg.m^−3^)^b)^	*γ*_break_ (%)^c)^	*τ*_strength_ (MPa)^d)^	*G* (GPa)^e)^	*T*_d5%_ (°C)^f)^	CR_800 °C_ (%)^g)^
*r*(Me‐PA‐mea)	0.0009	1358 ± 66	4.1 ± 1.8	47.2 ± 16.2	1.3 ± 0.3	259	36.7
*r*(Et‐PA‐mea)	0.0014	1348 ± 117	7.4 ± 0.7	53.8 ± 1.4	0.9 ± 0.1	257	38.2
*r*(Me‐DPA‐mea)	0.2220	1317 ± 109	3.7 ± 1.0	31.2 ± 6.5	1.0 ± 0.1	259	25.2
*r*(Et‐DPA‐mea)	0.0023	1410 ± 166	3.1 ± 0.4	21.1 ± 7.1	0.8 ± 0.1	243	25.0
*r*(Pr‐DPA‐mea)	0.0022	1105 ± 30	3.1 ± 0.4	23. 9 ± 4.3	0.9 ± 0.1	259	26.4
*r*(Bu‐DPA‐mea)	0.0012	1292 ± 257	5.4 ± 2.1	40.4 ± 11.4	0.9 ± 0.2	265	27.3

^a)^ Average porosity calculated by µCT.

^b)^ Average bulk density.

^c)^ Shear strain at break.

^d)^ Ultimate shear strength (shear stress at break).

^e)^ Shear modulus (shear testing, 0.5%·min^−1^).

^f)^ Temperature of 5 wt.% thermal degradation (N_2_ atmosphere).

^g)^ Char yield at 800 °C.

^13^C solid‐state (SS) NMR was recorded on Me‐DPA‐mea, *p*(Me‐DPA‐mea), and *r*(Me‐DPA‐mea) to evaluate the impact of the mechanical reprocessing at a molecular level (Figure 7c). Once foamed, the typical resonance peak of oxazine rings (N─CH_2_*─O located at *δ* = 84 ppm in the ^13^C SS‐NMR spectrum of Me‐DPA‐mea) totally vanished, confirming the near‐quantitative ring‐opening of benzoxazine moieties. In the aliphatic region of the ^13^C SS‐NMR spectrum of *p*(Me‐DPA‐mea), the intensity of the peak corresponding to the unreacted methyl ester (CH_3_*─O, *δ* = 51 ppm) significantly reduced. This decrease, even more pronounced after thermo‐reprocessing of *r*(Me‐DPA‐mea), originates from irreversible TER and the release of methanol self‐blowing agent (Scheme 2a). In addition, the widening of the methylene signal chemically bonded to a heteroatom (nitrogen or oxygen, *δ* = 55–65 ppm) suggests the formation of *β*‐aminoester moieties generated through irreversible TER. In the carbonyl region, the ester bond peak (*δ* = 174 ppm) is gradually widening after foaming and reprocessing, further evidencing the occurrence of irreversible TER of alkylester bond during mechanical reprocessing.

## Conclusion

This work presents the first reported example of using dynamic covalent exchanges in vitrimers to produce a self‐blowing agent, enabling the formation of reprocessable vitrimeric foams. The concept relies on the release of an alcohol through irreversible transesterification (TER), triggered by the ring‐opening polymerization of benzoxazine‐based precursors. Alcohol release occurs during the polymerization, when the material transits from the rubbery to the vitreous sol‐gel states, allowing its foaming, with volume expansion reaching up to 850% in some cases. The density, volume expansion, and thermal and mechanical properties were highly dependent on the functionality of the benzoxazine precursors and the type of alcohol released. Monofunctional precursors produced low‐density (160 kg·m^−^^3^) but brittle foams, while difunctional precursors resulted in extremely rigid and durable foams, with compressive Young's modulus ranging from 20 to 38 MPa. The optimized foaming conditions enable the manufacture of a series of foams with dimensional stability and open‐cell morphology (*e* = 67%–86%). Finally, as the resulting foams consist of aliphatic ─OH and ester bonds (alkylester and *β*‐aminoester) internally catalyzed by tertiary amines, the mechanically reprocessed product was converted into a resin, offering a foam‐to‐resin reprocessability aptitude.

## Conflict of Interests

The authors declare no conflict of interest.

## Supporting information



Supporting Information

## Data Availability

The data that support the findings of this study are available in the Supporting Information of this article.
