# Supplementary material for: Dynamic Exchange Reactions as Self‐Blowing Agents for the Production of Reprocessable Foams
Source: Angew Chem Int Ed Engl. 2025 May 24;64(28):e202502970. doi: 10.1002/anie.202502970 (PMC12232877; doi:10.1002/anie.202502970)
Supplement: Supplementary file 1 — Supporting Information [file ANIE-64-e202502970-s001.docx]

**Dynamic Exchange Reactions as Self-Blowing Agents for the Production of Reprocessable Foams**

Antoine Adjaoud ^a^, Anaë Girault-Fodil ^a,b^, Farida Baraka ^c^, Vincent Boulic ^a ,b^, Benoit Marcolini ^a^, Laura Puchot ^a^, and Pierre Verge ^a, *^

*^a^* Luxembourg Institute of Science and Technology, 5 Avenue des Hauts-Fourneaux, L-4362, Esch-sur-Alzette, Luxembourg

^b^ Department of Physics and Materials Science, University of Luxembourg, 2 Avenue de l’Université, L-4365, Esch-sur-Alzette, Luxembourg

^c^ Biorefinery Processes Group, Chemical and Environmental Engineering Department, Engineering Faculty of Gipuzkoa, University of the Basque Country UPV/EHU, Plaza Europa 1, 20018 Donostia, Spain

* Corresponding author: pierre.verge@list.lu

# Experimental

**Materials and chemicals:** 3-(4-hydroxyphenyl) propionic acid (98 %, phloretic acid, PA), 4,4-bis(4-hydroxyphenyl)valeric acid (95 %, diphenolic acid, DPA), 1-propanol (≥ 99.5%, Pr), 1-butanol (99.9 %, Bu), *para*-toluene sulfonic acid monohydrate (≥ 98.5 %, *p*TSA), 2-aminoethanol (> 98 %, monoethanolamine, mea), and paraformaldehyde (95 %, PFA) were purchased from Sigma-Aldrich^®^. Extra pure methanol (≥ 99.5 %, Me) and absolute ethanol (>99.5 %, Et) were purchased from Fischer Chemical^TM^. All solvents and chemicals were used as received without any purification.

**Synthetic procedure:** The synthesis of alkylester-based benzoxazine precursors followed a similar two-steps chemical pathway reported in our previous work with minor modification.^[1]^ Phenolic acid precursors (PA, DPA) were reacted with an excess of alcohol solvents (Me, Et, Pr, and Bu; labelled as R) in the presence of *p*TSA as an acid catalyst (0.5 wt.%) *via* Fischer esterification. After 12 h of reaction at the reflux temperature of alcohol solvents under magnetic stirring (500 rpm), the reaction media was cooled to room temperature and concentrated under reduced pressure. Then, the extract was redissolved in chloroform (R-PA) or butanone (R-DPA) and purified by three liquid−liquid extractions with 5% sodium bicarbonate (NaHCO_3_), followed by three liquid−liquid extractions with ultrapure water (H_2_O, Merck Millipore Milli-Q™). The organic layer was dried over magnesium sulfate (MgSO_4_), evaporated under reduced pressure, dried overnight under reduced pressure (<1 mBar, 50°C) and recovered mostly as fine powder (pure yield ≥ 70%). In a second step, the alkylester intermediates (R-PA or R-DPA, 1.0 equiv) were reacted solventless with paraformaldehyde (PFA, 2.0 or 4.0 equiv), and mono-ethanolamine (1.0 or 2.0 equiv) at 70°C for 3h under magnetic (R-PA-mea, 250 rpm) or mechanical stirring (R-DPA-mea, Ministar 20 Digital, 250 rpm) *via* a Mannich-like condensation. All alkylester-based benzoxazine precursors were solubilized in chloroform and thoroughly dried over MgSO_4_. After evaporation of the solvent under reduced pressure, the precursors were dried overnight under reduced pressure (<1 mBar, 50°C), and used without any other purification. Precursors were obtained in almost quantitiative mass yield as viscous liquid (R-PA-mea) or solid powder (R-DPA-mea).

**Preparation of alkylester-based polybenzoxazine foams:** The foaming process was performed in a convection oven using typically 2.5 g of alkylester-based benzoxazine precursor placed into a sealed 15 mL polytetrafluoroethylene sample tub (PTFE, chemically inert and autoclavable). For larger container, a similar ratio between monomer’s weight and container’s capacity was maintained. The sample is heated from room tempreature to 130°C (for R-DPA-mea) or directly introduced in the convection oven at 130°C (R-PA-mea). After complete softening of the monomer at 130°C during 10 minutes, the temperature is then increased to 180, 200, or 220°C at a 5°C·min^-1^ rate and maintained for a period of 20 min to maximize the rate of cross-linking reactions. (fan= 50%). The sample is weighted before and after the process to calculate the experimental weight loss with formula (1):

$\text{W}\text{experimental}\text{ (\%)=}\frac{\text{W}\text{i}\text{-W}\text{f}}{\text{W}\text{i}} \text{x 100}$ (S1)

with W_i_ corresponding to the weight of the dried monomer before the foaming process and W_f_ corresponding to the weight of the sample after the foaming process.

Table S1 summarizes the theoretical weight loss for each precrusors considering that all alkylester (1 eq.) has quantitatively reacted to release the alcohol solvent adduct of irreversible transesterification reactions. Assuming that the precursors are properly dried and only the alcohol solvent is released during the foaming process (and responsible for the weight loss), the extent rate of irreversible transesterification (Y_TER_) can be calculated according to equation (S2):

$\text{Y}\text{TER}\text{ (\%)=}\frac{\text{W}\text{experimental}}{\text{W}\text{theoretical}}$ x 100 (S2)

with W_experimental_ corresponding to the experimental weight loss and W_theoretical_ corresponding to the theoretical ones.

**Table S1** Theoretical weight loss considering quantitative irreversible transesterification of alkylester-based benzoxazine precursors.

| Molecule | Average bulk density (kg·m^-3^) | M (g·mol^-1^) | W_theoretical_ (%) |
| --- | --- | --- | --- |
| Me-PA-mea | 1450 | 265.3 | 12.1 |
| Et-PA-mea | 1420 | 279.4 | 16.5 |
| Pr-PA-mea | 1360 | 293.4 | 20.4 |
| Bu-PA-mea | 1300 | 307.4 | 24.1 |
| Me-DPA-mea | 1390 | 470.5 | 6.8 |
| Et-DPA-mea | 1330 | 484.6 | 9.5 |
| Pr-DPA-mea | 1200 | 498.6 | 12.0 |
| Bu-DPA-mea | 1120 | 512.6 | 14.4 |

The volumetric expansion of the polybenzoxazine foam is controlled by the release of the self-blowing agent and buble coalescence filling the free-volume of the container. The upper surface of the regular cylinder was polished to obtain parallel surface (Tegramin-25 Stuers^®^, silicon carbide grinding paper grit 80). As the foam expansion is oriented solelyl toward the vertical direction, the volumetric expansion can be calculated according equation S3:

$\text{V}\text{exp }\text{(\%)=}\frac{\text{H}\text{foam }\text{- H}\text{monomer}}{\text{H}\text{monomer}} \text{x 100}$ (S3)

with H_monomer_ corresponding to the initial height of the monomer and H_foam_ the final hieght of the foam after surface’s polishing.

The extent of the foaming process (η_foaming_), corresponding to the actual volume of alcohol self-blowing agent generated to produce the foam structure, can be calculated according equation S4:

$\text{η}\text{foaming }\text{(\%)=}\frac{\text{V}\text{exp}}{\text{V}\text{exp}\text{,max}}$ x 100 (S4)

with V_exp,max_ corresponding to the maximum volumetric expansion considering the total volume of alcohol self-blowing agent generated through irreversible TER (following the optimized foaming process) and V_exp_ the volumetric expansion (actual volume of alcohol vapor produced to generate the foam structure). V_exp,max_ is calculated as follows:

$\text{V}\text{exp,max }\text{(\%)=}\frac{\text{V}\text{alcohol,experimental}\text{ x }\text{ρ}\text{monomer }}{\text{m}\text{monomer}}$ x 100

with V_alcohol,experimental_ corresponding to the total volume of alcohol gas generated through irreversible TER (following the optimized foaming process of each monomer), ρ_monomer_ the average bulk density of the monomer, and m_monomer_ the initial mass of monomer. V_alcohol,experimental_ is calculated as follows:

$$\text{V}\text{alcohol,experimental }\text{(mL)= n}\text{alcohol,theoretical}\text{ }\text{x Y}\text{TER}\text{ }\text{x }\text{V}\text{mol}$$

with $\text{n}\text{alcohol,theoretical}$ corresponding to the molar quantity of alcohol gas generated through irreversible TER following the optimized foaming process of each monomer, Y_TER_ the extent rate of irreversible transesterification following the optimized foaming process of each monomer, and V_mol_ the molar volume of an ideal gas.

The average bulk density (ρ) of the self-blown polybenzoxazine foams was calculated by dividing the foam’s weight over the bulk volume that was defined by assimilating the foam to a regular cylinder.

**Reprocessing of self-blown polybenzoxazine foams:** The foams were grinded into powder and compressed in a hydraulic press at 190°C for 30 min under 20 MPa pressure in a disk shape mold (h= 1 mm, ⌀= 20 mm).

**Determination of the cross-linking density of reprocessed polybenzoxazine resins:** The cross-linking density of the reprocessed resins was quantitatively assessed within the rubbery plateau region in torsional rhelogy temperature sweep-curve experiment. The molecular weight between crosslinks can be calculated according equation S5:

$\text{Mc}\text{ }\text{(g·mol}\text{-1}\text{) =}\frac{\text{R x T x }\text{ρ}}{\text{G'}\text{rubbery}}$ (S5)

with R corresponding to the universal gas constant, T the absolute temperature, ρ the density of the polymer, and G’_rubbery_ the shear storage modulus within the rubbery pateau region (T_α_ + 40°C).

Alternatively, the crosslinking density of the reprocessed resin can be calculated using equation (S6):

$\text{ν}\text{e }\text{(}\text{mol}\text{·}\text{cm}\text{-3}\text{) =}\frac{\text{G'}\text{rubbery}\text{ }}{\text{R x T }}$ (S6)

# Equipment and characterizations

**Nuclear Magnetic Resonance (NMR)** spectroscopy was performed on an AVANCE III HD Bruker spectrometer operating at a proton frequency of 600.16 MHz and equipped with a 5 mm BBO-probe. All chemical shifts are given as δ value (ppm) referenced to tetramethyl silane (TMS) as an internal standard. Assignments were performed using a combination of ^1^H, ^13^C, COSY, HSQC, and HMBC spectra. Peak multiplicity was indicated as follows: singlet (s); doublet (d); triplet (t) or multiplet (m). The coupling constants (J) were reported in Hertz (Hz). Solid State NMR spectra were recorded on the similar spectrometer equipped with a 4 mm Dual Channel MAS probe. Samples were loaded into 4 mm ZrO_2_ rotors capped with KEL-F stoppers. ^13^C-^1^H cross-polarization spectra were obtained acquiring 1024 or 10240 transients using a ramped (70-100%) contact of 2 ms at 62.5 kHz field on the first spinning sideband with a recycling delay of 5 s.

**Elemental analysis (CHNS/O measurements)** was performed on a Vario MACRO cube (Elementar France SARL). Samples were put into an oxygen-enriched furnace at 1150°C, where a combustion process converted carbon to carbon dioxide; hydrogen to water; nitrogen to nitrogen gas/oxides of nitrogen and sulfur to sulfur dioxide. The combustion products were heated separately to the corresponding desorption temperature in order to release the components as follows: CO_2_ (T_desorption_ = 240°C), H_2_O (T_desorption_ = 150°C) and SO_2_ (T_desorption_ = 100°C or 230°C).

**Differential scanning calorimetry (DSC)** thermograms were recorded on a Netzsch DSC 204 F1 Phoenix device in standard pierced aluminum crucibles (40 μL) and a sample mass of 5-10 mg. A linear heating ramp at a constant heating rate was applied from 25 to 300°C under a nitrogen flow rate (N_2_, 40 mL·min^-1^). Exothermic phenomenons shown with a positive signal (exo up).

**Rheology** experiments were recorded using an Anton Paar Physica MCR 302 rheometer equipped with a CTD 450 temperature control device. The rheo-kinetic measurements were performed using small quantities of the samples loaded in a parallel plate-plate geometry (Ø= 25 mm, gap 0.5 mm). The cross-linking reactions were investigated by monitoring the evolution of the complex viscosity (η*) in the oscillation mode at a controlled strain of 0.1% (1 Hz). Heating ramps of 20°C·min^-1^ was applied to reach the targeted temperature in isothermal conditions. Heating ramp of 2°C·min^-1^ was applied in temperature sweep curve experiment. The viscosity (η) was determined in isothermal conditions at 50°C at a shear rate ranging logarithmically from 0.5 to 50 s^-1^. Rheology temperature sweep curve experiment was performed on bar-shaped material in the torsion-mode under constant deformation of 0.1% at a frequency of 1 Hz and a heating rate of 2°C·min^-1^ (sample size: length:width:thickness = 25mm:5mm:1.25mm). The α-mechanical relaxation (T_α_) was determined from the maximum of the loss factor curves peak. The shear stress-strain curves were measured at room temperature on the reprocessed resins at a shear rate of 0.5 %.min^-1^ until rupture of the material (sample size: length:width:thickness = 25mm:5mm:1.25mm). The shear modulus was determined on the slope of the shear stress–strain curve within the linear range and calculated as the mean value of three measurements.

**Thermogravimetric analysis coupled with micro-gas chromatography (TGA- micro GC)** was completed on the Mettler Toledo TGA 2 device coupled with SOLIA 490 micro-GC.^[2]^ TGA experiment was conducted on 10 mg of sample in a ceramic alumina pan from 25 to 800°C (10°C·min^-1^) under an inert atmosphere (N_2_, 20 mL·min^-1^). During the same time, micro GC scans were acquired every two minutes (each data point corresponds to an individual micro-GC injection). For the quantitative analysis, the analyser was calibrated using standard alcohol solvents. The integration of the area of the alcohol peak is normalized to the alcohol’s solvent standard and is used to monitor the progress of transesterification reactions. Details on the retention time of the carrier gas and standard gas sample used for the calibration are provided in Table S2. The relative integration of the alcohol peak’ area was performed on module C using Soprane II software.

**Table S2** Retention time of the carrier gas and alcohol standards on the micro-GC column.

| Module | A | B | C |
| --- | --- | --- | --- |
| Retention time  carrier gas (s) | 39 - 39.2 / 39.7 – 40.2 | 17.8 / 63.1 – 63. 4 | 16.3 – 16.4 / 19.3 – 19.5 |
| Retention time  methanol standard (s) | 35.2 | 105.0 – 107.0 | 21.9 – 22.1 |
| Retention time  ethanol standard (s) | n.a. | n.a. | 26.5 – 26.7 |
| Retention time propanol standard (s) | n.a. | n.a. | 39.7 |
| Retention time  butanol standard (s) | n.a. | n.a. | 67.1 – 67.2 |

**Micro-computed X-ray tomography (μCT)** was performed on a laboratory x-ray cone-beam CT system EasyTom 160 produced by RX Solutions. The acquired projections were used as an input for the three-dimensional volume reconstruction by using the software Xact64. Geometrical corrections and ring artefacts attenuation were previously applied. Finally, the volume reconstruction was carried out by means of the filtered back-projection algorithm implemented in the software Xact64. Three-dimensional (3D) image analysis of μCT results was carried out with the commercial software Avizo. The average porosity (e) of the self-blown foams was determined in a four steps process: 1) editing a cylinder fully included in the foam, 2) threshold the voids within the cylinder and extract its 3D volume, 3) threshold the voids and the matrix within the cylinder and extract its volume, and finally 4) the average porosity is obtained by dividing the volume obtained of the voids (step 2)) by the total volume of the cylinder (step 3). The cell’s wall thickness was determined on the condensed phase by thresholding the matrix part of the self-blown polybenzoxazine foam and applying the thickness map option. This option consists of attributing to each voxel the local diameter of the largest ball involved in the object containing the voxel (classified in 100 µm ranged bin).

**Compression tests** were conducted on an Instron 5967 compression machine equipped with a 500 N load cell. The samples were compressed with a traverse speed of 2 mm·min^-1^ until they reached 10% of their initial thickness. The Young modulus was determined on the slope of the stress–strain compression curve within the linear range and calculated as the mean value of three measurements.

**Thermogravimetric analysis (TGA)** was completed on the Mettler Toledo TGA 2 device in ceramic alumina pan from 25 to 800°C at 10°C·min^-1^ rate under inert atmosphere (N_2_).

**Alkylester-based benzoxazine precursors**

-
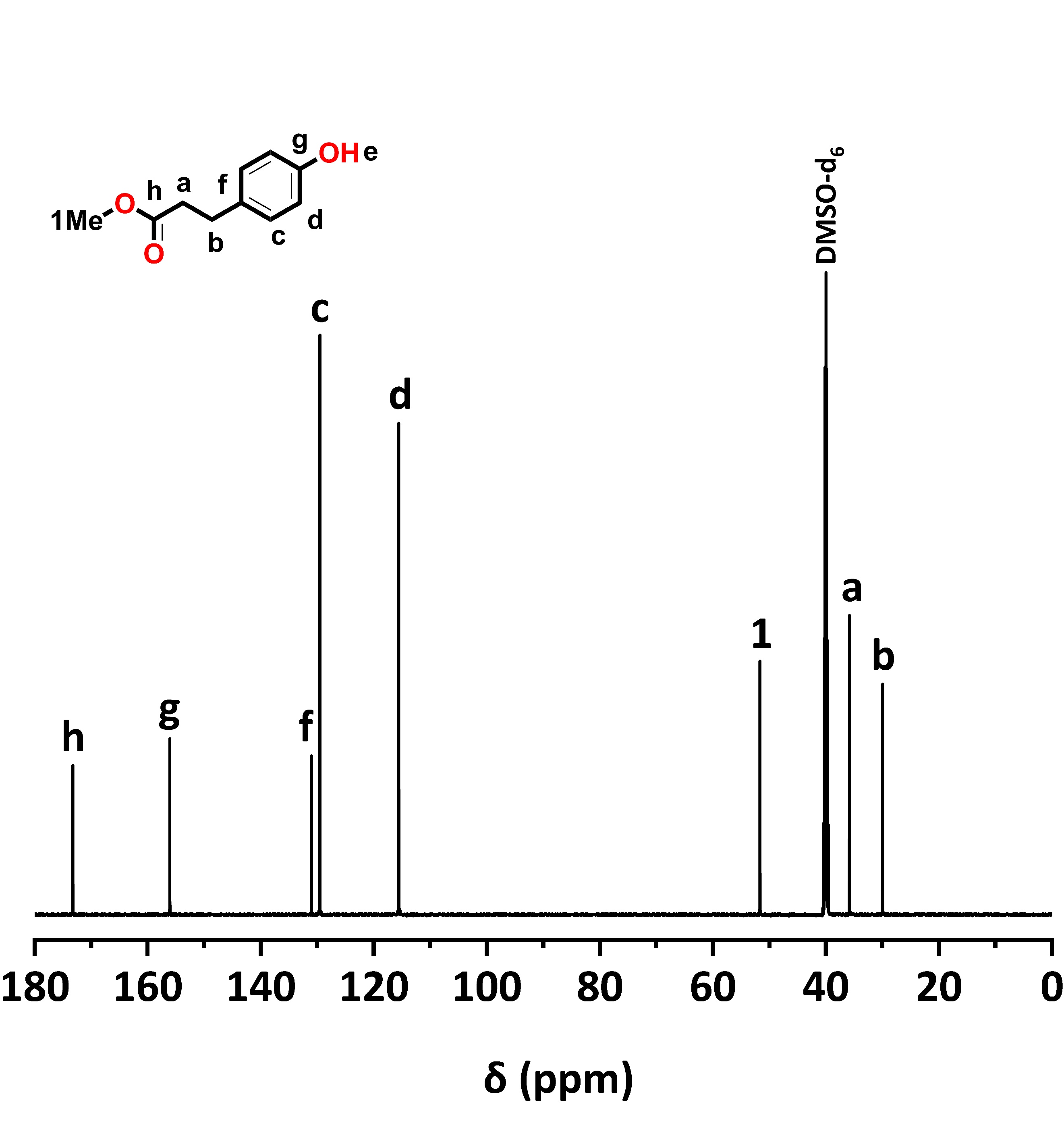

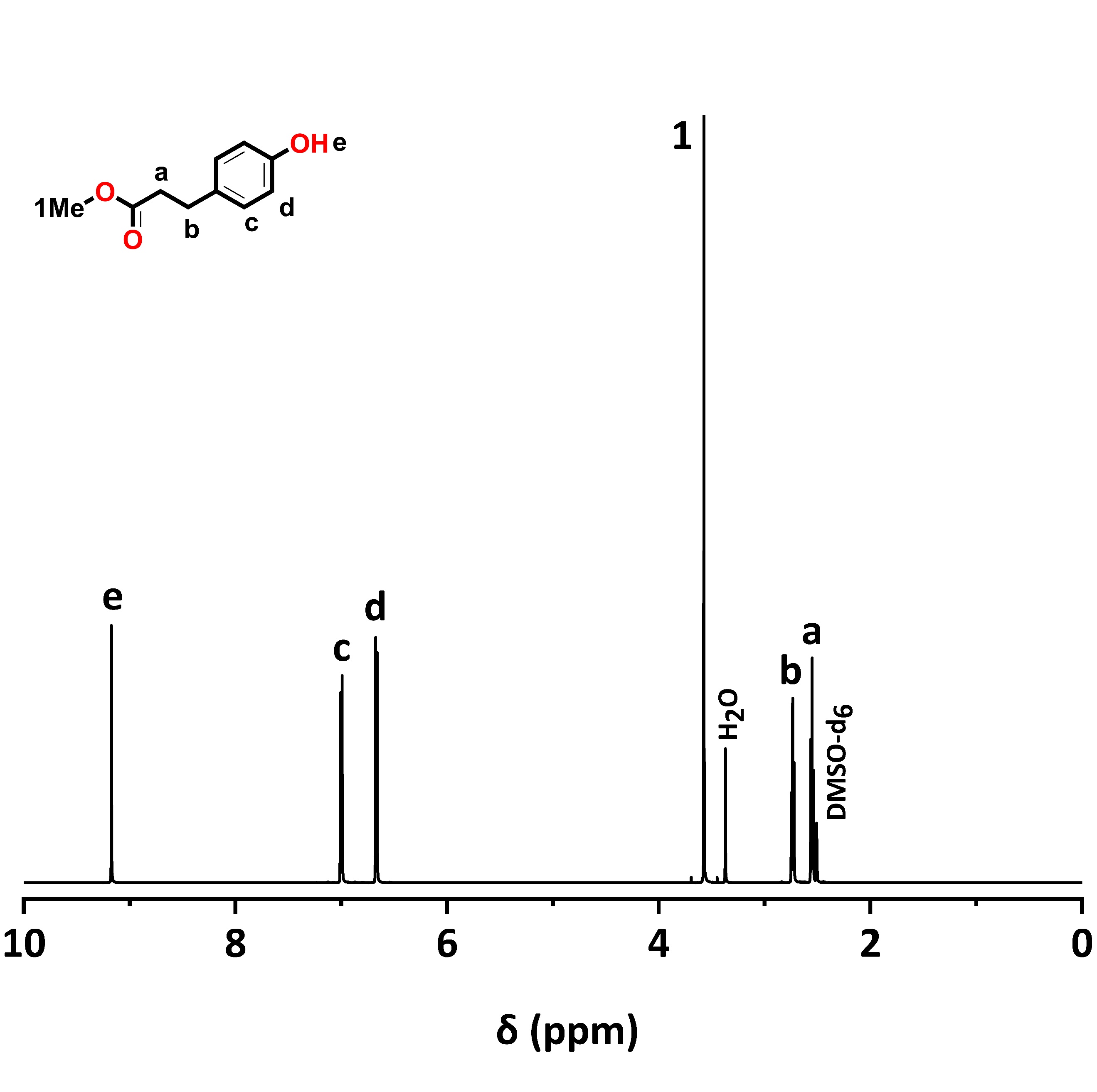
*Me-PA (Y= 73 wt.% after purification)*

**Figure S1** a) ^1^H and b) ^13^C NMR spectra of Me-PA.

**a) b)**

^1^H NMR (DMSO-d_6_, 600 MHz, 298°K): δ (ppm)= (assignment, multiplicity (coupling constant), [attribution], experimental integration, theoretical integration). δ= 2.55 (CH_2_-CH_2_*-C=O, t (J = 7.70 Hz), [a], exp 2.00H, th 2.00H); δ= 2.73 (CH_2_-CH_2_*-Ar, t (J = 7.61 Hz), [b], exp 2.01H, th 2.00H); δ= 3.57 (CH_3_*-O-C=O, s, [1], exp 3.00H, th 3.00H); δ= 6.66 (CH=CH*-C-OH, d (J = 8.45 Hz), [d], exp 1.98H, th 2.00H); δ= 6.99 (C-C-CH*=CH, d (J = 8.45 Hz), [c], exp 1.98H, th 2.00H); δ= 9.17 (Ar-OH*, s, [e], exp 0.99H, th 1.00H).

^13^C NMR (DMSO-d_6_, 600 MHz, 298°K): δ (ppm)= 30.0 [b]; 35.8 [a]; 51.7 [1]; 115.6 [d]; 129.5 [c]; 131.0 [f]; 156.1 [g]; 173.2 [h].

-
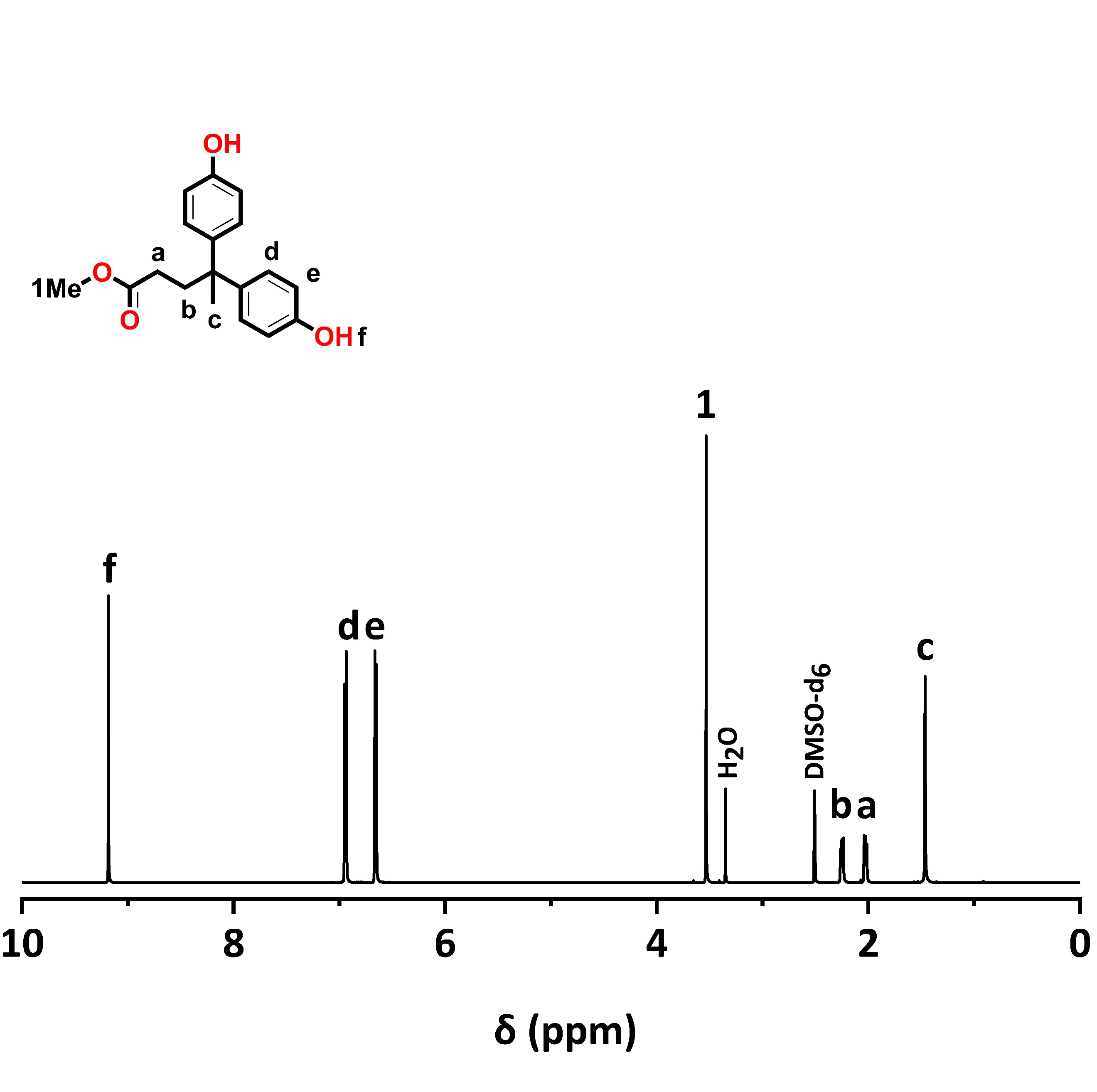

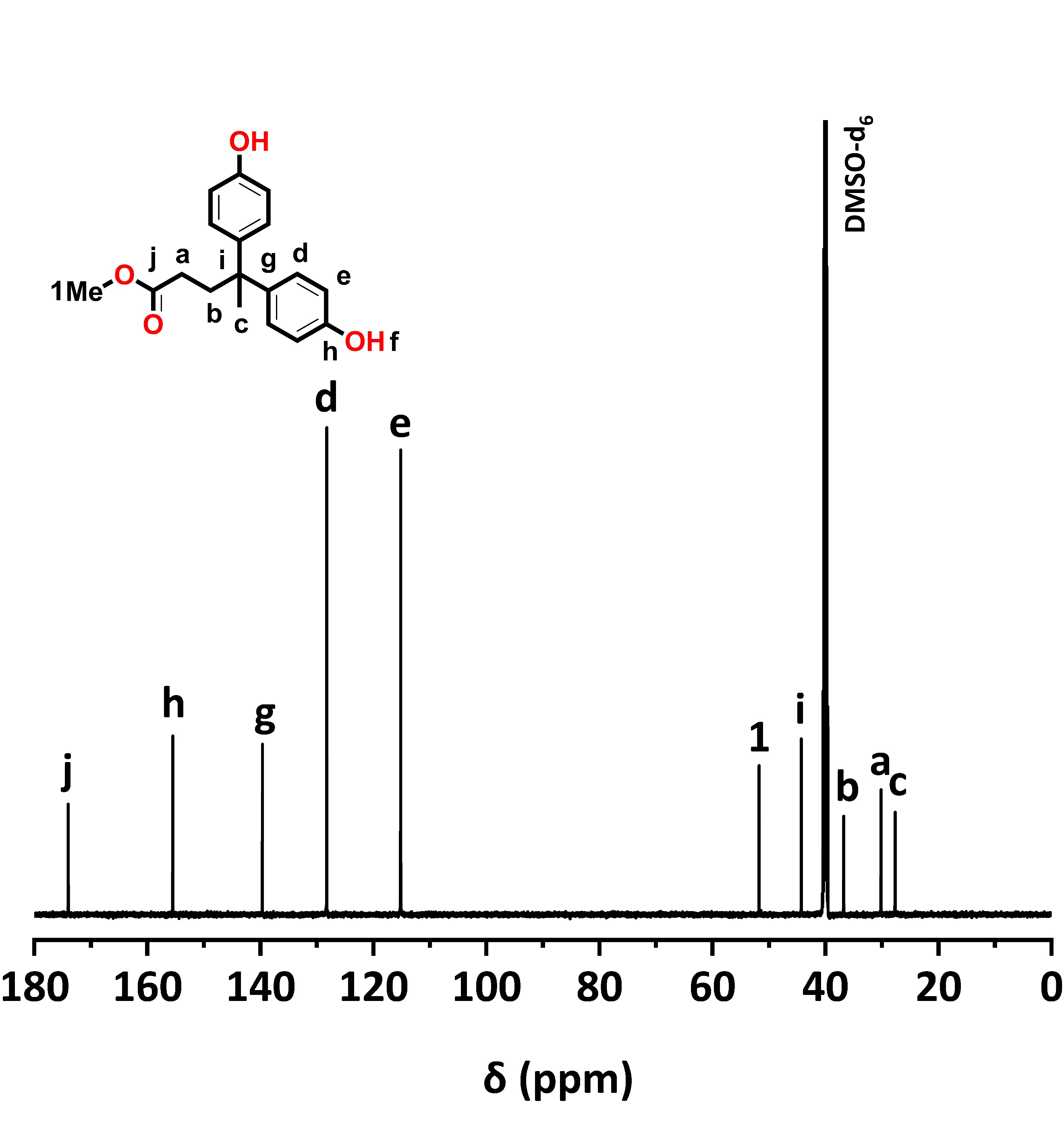
*Me-DPA (Y= 86 wt.% after purification)*

**Figure S2** a) ^1^H and b) ^13^C NMR spectra of Me-DPA.

**a) b)**

^1^H NMR (DMSO-d_6_, 600 MHz, 298°K): δ (ppm)= (assignment, multiplicity (coupling constant), [attribution], experimental integration, theoretical integration). δ= 1.46 (CH_3_*-C, s, [c], exp 3.00H, th 3.00H); δ= 2.03 (CH_2_-CH_2_*-C=O, t (J = 8.19 Hz), [a], exp 2.02H, th 2.00H); δ= 2.25 (CH_2_-CH_2_*-C, t (J = 8.18 Hz), [b], exp 2.00H, th 2.00H); δ= 3.53 (CH_3_*-O-C=O, s, [1], exp 3.00H, th 3.00H); δ= 6.65 (CH=CH*-C-OH, d (J = 8.72 Hz), [e], exp 4.00H, th 4.00H); δ= 6.94 (C-C-CH*=CH, d (J = 8.72 Hz), [d], exp 4.02H, th 4.00H); δ= 9.18 (Ar-OH*, s, [f], exp 2.00H, th 2.00H).

^13^C NMR (DMSO-d_6_, 600 MHz, 298°K): δ (ppm)= 27.7 [c]; 30.2 [a]; 36.8 [b]; 44.3 [i]; 51.7 [1]; 115.2 [e]; 128.2 [d]; 139.6 [g]; 155.5 [h]; 174.0 [j].

- *Et-PA (Y= 76 wt.% after purification)*

**Figure S3** a) ^1^H and b) ^13^C NMR spectra of Et-PA.

**a) b)**


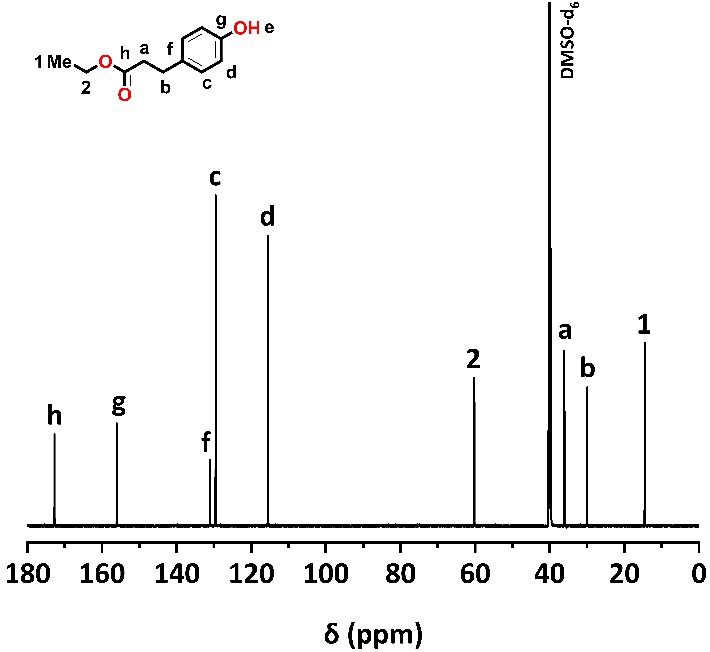

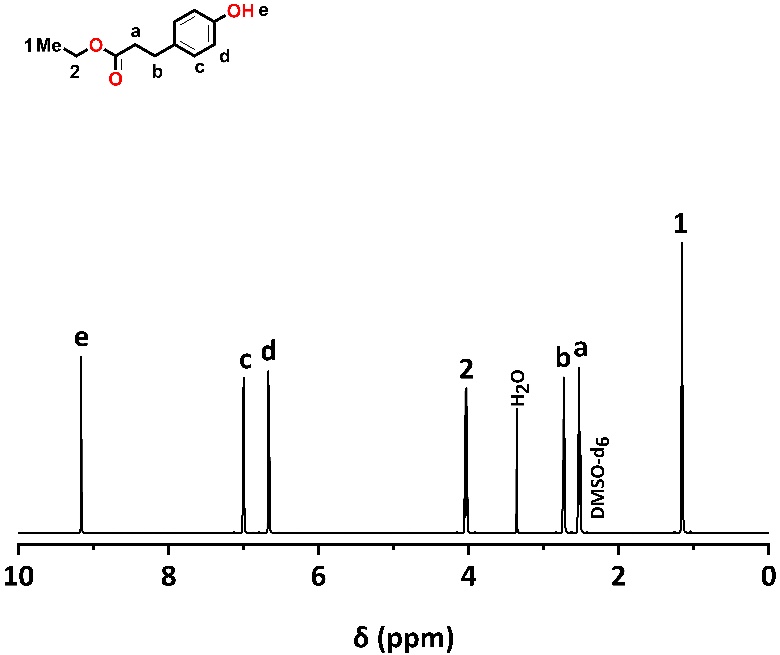


^1^H NMR (DMSO-d_6_, 600 MHz, 298°K): δ (ppm) = (assignment, multiplicity (coupling constant), [attribution], experimental integration, theoretical integration). δ= 1.15 (CH_3_*-CH_2_-O, t (J = 7.11 Hz), [1], exp 3.00H, th 3.00H); δ= 2.53 (CH_2_-CH_2_*-C=O, t (J = 7.73 Hz), [a], exp 2.03H, th 2.00H); δ= 2.73 (CH_2_-CH_2_*-Ar, t (J = 7.55 Hz), [b], exp 2.07H, th 2.00H); δ= 4.03 (CH_3_-CH_2_*-O, quad (J= 7.11 Hz), [2], exp 2.02H, th 2.00H); δ= 6.66 (CH=CH*-C-OH, d (J = 8.17 Hz), [d], exp 2.00H, th 2.00H); δ= 6.99 (C-C-CH*=CH, d (J = 8.17 Hz), [c], exp 2.01H, th 2.00H); δ= 9.16 (Ar-OH*, s, [e], exp 1.01H, th 1.00H).

^13^C NMR (DMSO-d_6_, 600 MHz, 298°K): δ (ppm) = 14.6 [1]; 30.0 [b]; 36.1 [a]; 60.2 [2]; 115.5 [d]; 129.6 [c]; 131.0 [f]; 156.1 [g]; 172.7 [h].

- *Et-DPA (Y= 91 wt.% after purification)*

**Figure S4** a) ^1^H and b) ^13^C NMR spectra of Et-DPA.

**a) b)**


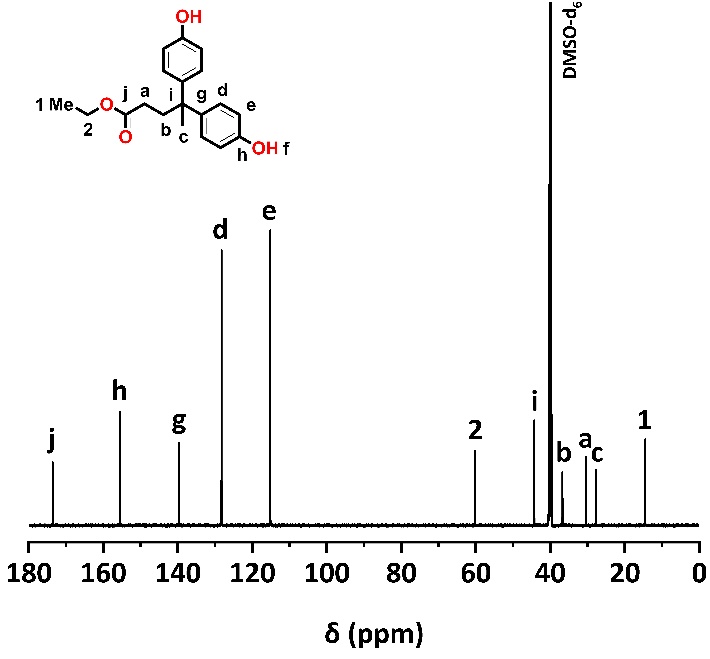

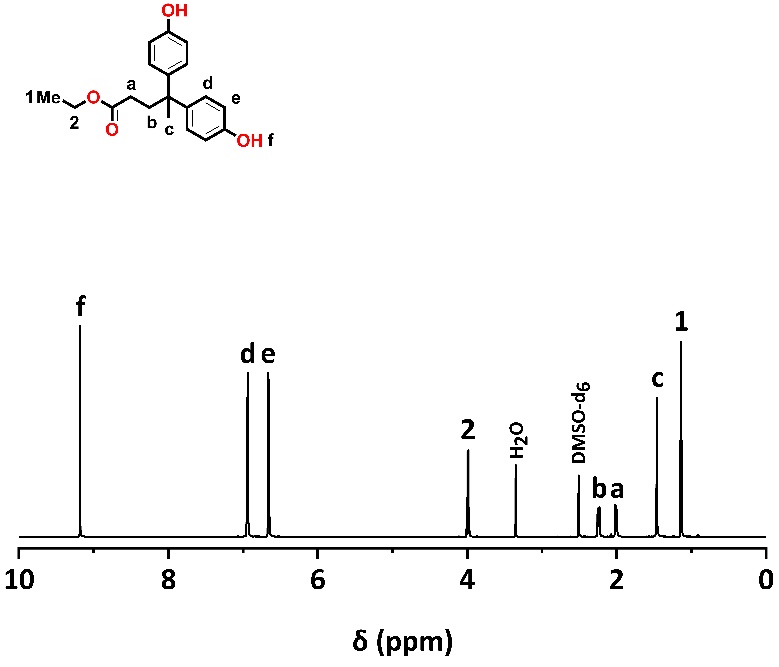


1H NMR (DMSO-d6, 600 MHz, 298°K): δ (ppm)= (assignment, multiplicity (coupling constant), [attribution], experimental integration, theoretical integration). δ= 1.14 (CH3*-CH2-O, t (J= 7.11 Hz), [1], exp 3.00H, th 3.00H); δ= 1.46 (CH3*-C, s, [c], exp 3.00H, th 3.00H); δ= 2.00 (CH2-CH2*-C=O, t (J = 8.09 Hz), [a], exp 1.99H, th 2.00H); δ= 2.24 (CH2-CH2*-C, t (J = 8.11 Hz), [b], exp 1.98H, th 2.00H); δ= 3.98 (CH3-CH2*-O, quad (J= 7.12 Hz), [2], exp 1.99H, th 2.00H); δ= 6.65 (CH=CH*-C-OH, d (J = 8.72 Hz), [d], exp 3.98H, th 4.00H); δ= 6.93 (C-C-CH*=CH, d (J = 8.72 Hz), [e], exp 4.00H, th 4.00H); δ= 9.18 (Ar-OH*, s, [f], exp 1.98H, th 2.00H).

13C NMR (DMSO-d6, 600 MHz, 298°K): δ (ppm)= 14.5 [1]; 27.7 [c]; 30.4 [a]; 36.7 [b]; 44.3 [i]; 60.2 [2]; 115.2 [e]; 128.2 [d]; 139.7 [g]; 155.5 [h]; 173.5 [j].

- *Pr-PA (Y= 90 wt.% after purification)*

**Figure S5** a) ^1^H and b) ^13^C NMR spectra of Pr-PA.

**a) b)**


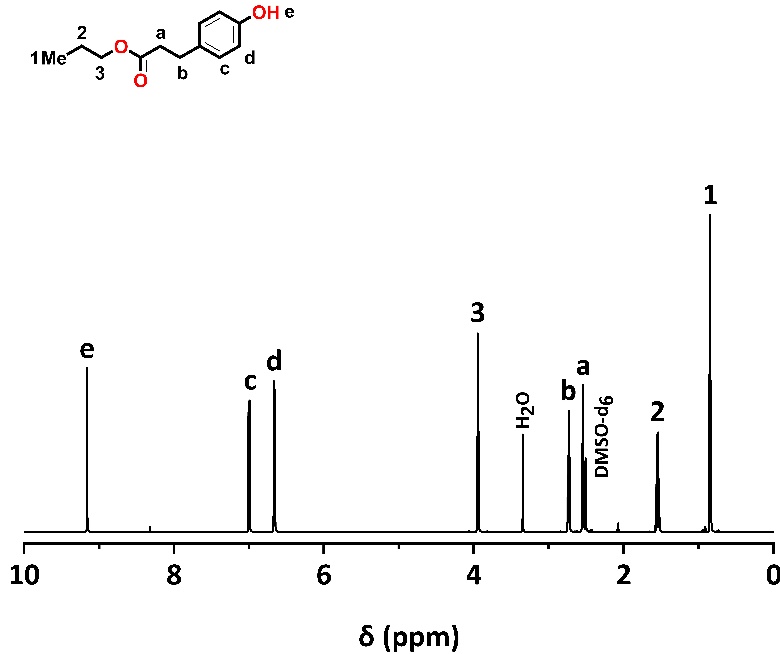

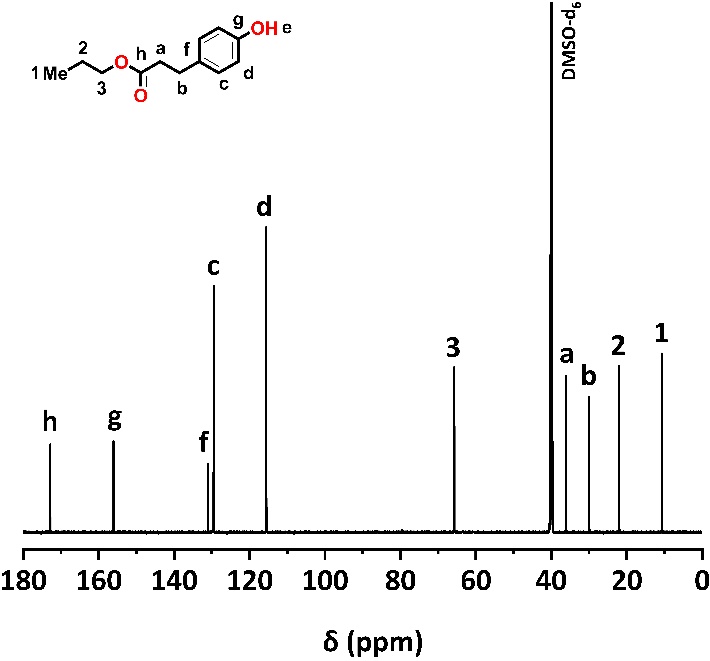


^1^H NMR (DMSO-d_6_, 600 MHz, 298°K): δ (ppm)= (assignment, multiplicity (coupling constant), [attribution], experimental integration, theoretical integration). δ= 0.85 (CH_3_*-CH_2_-CH_2_, d (J = 7.46 Hz), [1], exp 3.00H, th 3.00H); δ= 1.54 (CH_3_-CH_2_*-CH_2_, sex (J = 7.21 Hz), [2], exp 2.03H, th 2.00H); δ= 2.54 (CH_2_-CH_2_*-C=O, t (J = 7.59 Hz), [a], exp 2.04H, th 2.00H); δ= 2.73 (CH_2_-CH_2_*-Ar, t (J = 7.58 Hz), [b], exp 2.05H, th 2.00H); δ= 3.95 (CH_2_-CH_2_*-O, sept (J= 6.64 Hz), [2], exp 2.03H, th 2.00H); δ= 6.66 (CH=CH*-C-OH, d (J = 8.41 Hz), [d], exp 2.00H, th 2.00H); δ= 7.00 (C-C-CH*=CH, d (J = 8.65 Hz), [c], exp 2.02H, th 2.00H); δ= 9.16 (Ar-OH*, s, [e], exp 1.01H, th 1.00H).

^13^C NMR (DMSO-d_6_, 600 MHz, 298°K): δ (ppm)= 10.7 [1]; 22.0 [2]; 30.0 [b]; 36.0 [a]; 65.7 [3]; 115.5 [d]; 129.5 [c]; 131.0 [f]; 156.1 [g]; 172.8 [h].

- *Pr-DPA (Y= 92 wt.% after purification)*

**Figure S6** a) ^1^H and b) ^13^C NMR spectra of Pr-DPA.

**a) b)**


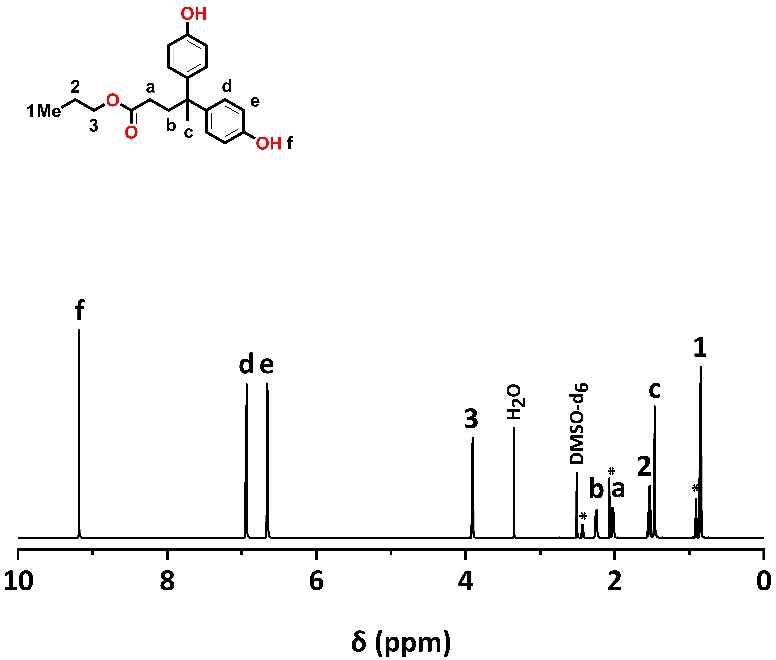

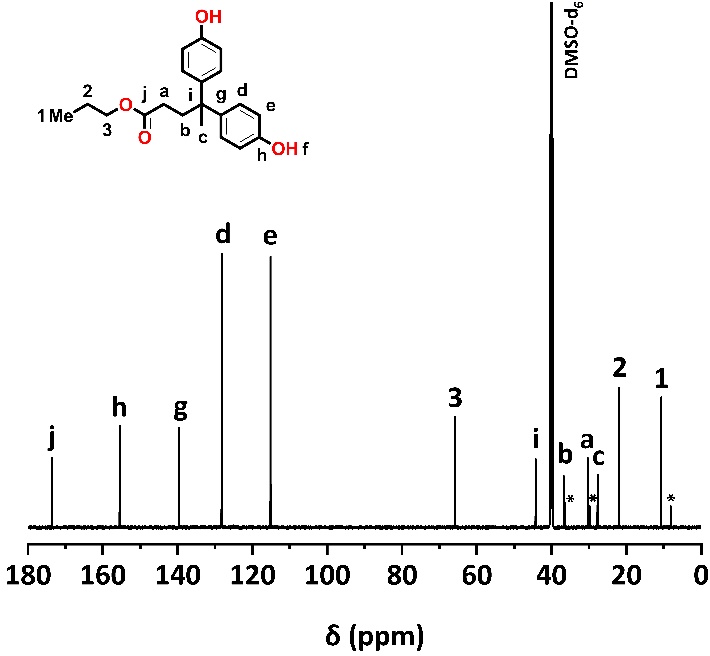


^1^H NMR (DMSO-d_6_, 600 MHz, 298°K): δ (ppm)= (assignment, multiplicity (coupling constant), [attribution], experimental integration, theoretical integration). δ= 0.85 (CH_3_*-CH_2_- CH_2_, t (J= 7.42 Hz), [1], exp 3.02H, th 3.00H); δ= 1.46 (CH_3_*-C, s, [c], exp 3.00H, th 3.00H); δ= 1.54 (CH_3_-CH_2_*- CH_2_, sex (J= 7.28 Hz), [2], exp 2.06H, th 2.00H); δ= 2.02 (CH_2_-CH_2_*-C=O, t (J = 8.03 Hz), [a], exp 2.01H, th 2.00H); δ= 2.25 (CH_2_-CH_2_*-C, t (J = 8.13 Hz), [b], exp 2.00H, th 2.00H); δ= 3.91 (CH_2_-CH_2_*-O, t (J= 6.77 Hz), [3], exp 2.00H, th 2.00H); δ= 6.65 (CH=CH*-C-OH, d (J = 8.78 Hz), [e], exp 4.01H, th 4.00H); δ= 6.94 (C-C-CH*=CH, d (J = 8.78 Hz), [d], exp 4.02H, th 4.00H); δ= 9.18 (Ar-OH*, s, [f], exp 2.01H, th 2.00H).

^13^C NMR (DMSO-d_6_, 600 MHz, 298°K): δ (ppm)= 10.7 [1]; 21.9 [2]; 27.7 [c]; 30.3 [a]; 36.7 [b]; 44.3 [i]; 65.7 [3]; 115.2 [e]; 128.2 [d]; 139.7 [g]; 155.5 [h]; 173.6 [j].

- *Bu-PA (Y= 71 wt.% after purification)*

**Figure S7** a) ^1^H and b) ^13^C NMR spectra of Bu-PA.

**a) b)**


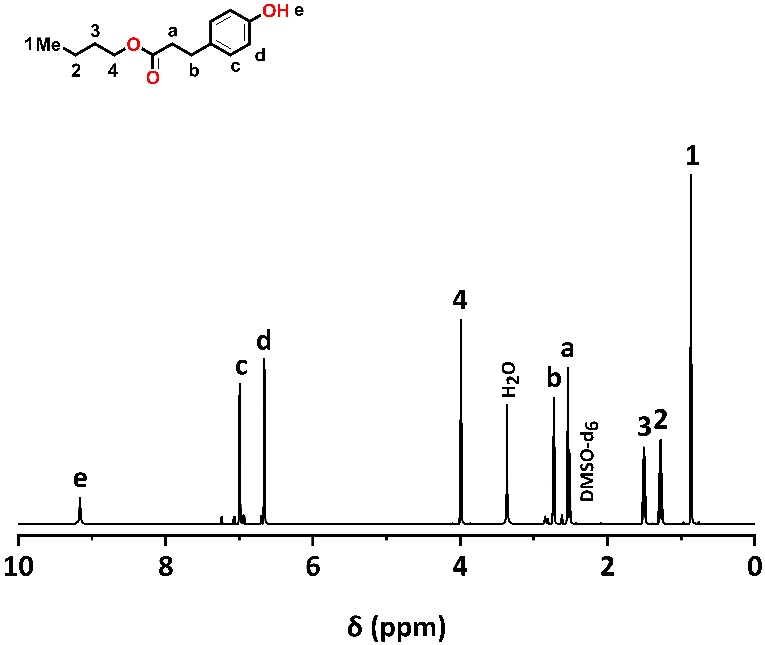

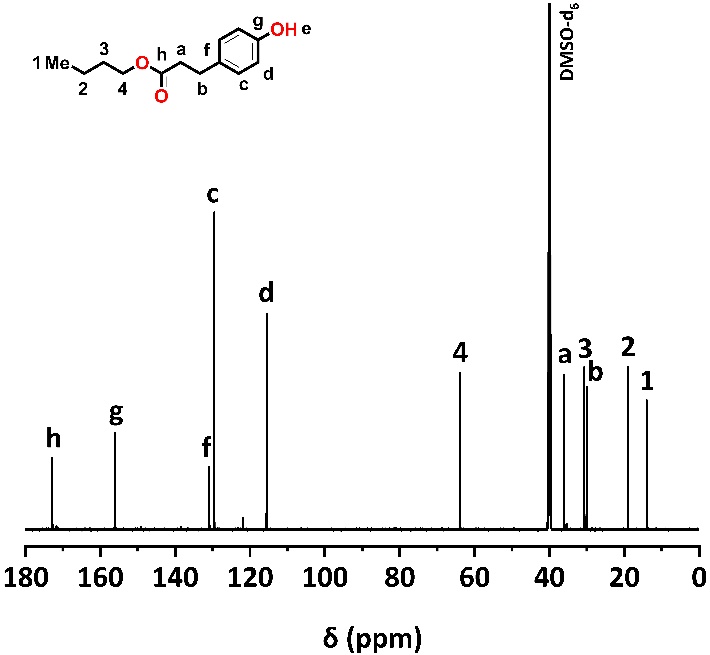


^1^H NMR (DMSO-d_6_, 600 MHz, 298°K): δ (ppm)= (assignment, multiplicity (coupling constant), [attribution], experimental integration, theoretical integration). δ= 0.87 (CH_3_*-CH_2_-CH_2_, t (J = 7.41 Hz), [1], exp 3.00H, th 3.00H); δ= 1.27 (CH_3_-CH_2_*-CH_2_, sext (J = 7.54 Hz), [2], exp 2.02H, th 2.00H); δ= 1.51 (CH_2_-CH_2_*-CH_2_, quint (J = 7.51 Hz), [3], exp 2.02H, th 2.00H); δ= 2.54 (CH_2_-CH_2_*-C=O, t (J = 7.59 Hz), [a], exp 1.89H, th 2.00H); δ= 2.73 (CH_2_-CH_2_*-Ar, t (J = 7.54 Hz), [b], exp 1.94H, th 2.00H); δ= 3.99 (CH_2_-CH_2_*-O, t (J= 6.70 Hz), [4], exp 2.01H, th 2.00H); δ= 6.66 (CH=CH*-C-OH, d (J = 8.47 Hz), [d], exp 1.99H, th 2.00H); δ= 6.99 (C-C-CH*=CH, d (J = 8.47 Hz), [c], exp 2.01H, th 2.00H); δ= 9.16 (Ar-OH*, s, [e], exp 1.00H, th 1.00H).

^13^C NMR (DMSO-d_6_, 600 MHz, 298°K): δ (ppm) = 14.0 [1]; 19.0 [2]; 30.0 [b]; 30.7 [3]; 36.0 [a]; 63.9 [4]; 115.5 [d]; 129.5 [c]; 131.0 [f]; 156.1 [g]; 172.8 [h].

-
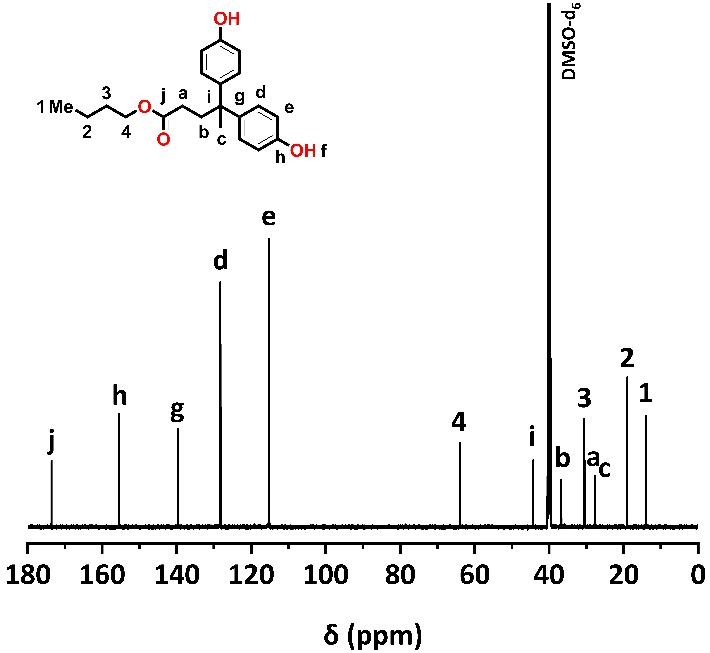

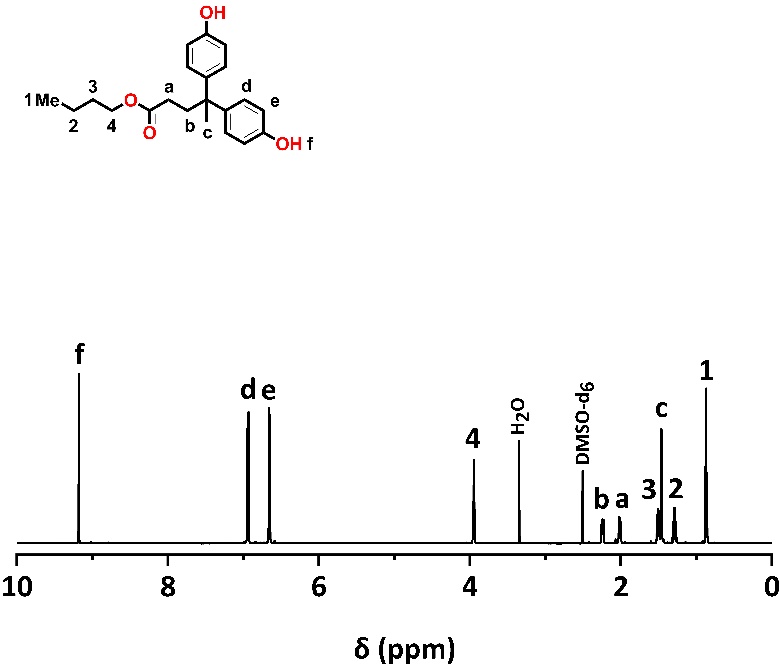
*Bu-DPA (Y= 67 wt.% after purification)*

**Figure S8** a) ^1^H and b) ^13^C NMR spectra of Bu-DPA.

**a) b)**

^1^H NMR (DMSO-d_6_, 600 MHz, 298°K): δ (ppm)= (assignment, multiplicity (coupling constant), [attribution], experimental integration, theoretical integration). δ= 0.87 (CH_3_*-CH_2_-CH_2_, t (J= 7.41 Hz), [1], exp 3.07H, th 3.00H); δ= 1.28 (CH_3_-CH_2_*-CH_2_, sex, [2], exp 2.10H, th 2.00H); δ= 1.46 (CH_3_*-C, s, [c], exp 3.00H, th 3.00H); δ= 1.50 (CH_2_-CH_2_*- CH_2_, quint. (J = 7.48 Hz), [3], exp 2.20H, th 2.00H); δ= 2.01 (CH_2_-CH_2_*-C=O, t (J = 8.05 Hz), [a], exp 2.00H, th 2.00H); δ= 2.24 (CH_2_-CH_2_*-C, t (J = 8.05 Hz), [b], exp 2.00H, th 2.00H); δ= 3.95 (CH_2_-CH_2_*-O, t (J= 6.63 Hz), [4], exp 1.98H, th 2.00H); δ= 6.65 (CH=CH*-C-OH, d (J = 8.71 Hz), [e], exp 3.99H, th 4.00H); δ= 6.94 (C-C-CH*=CH, d (J = 8.81 Hz), [d], exp 3.94H, th 4.00H); δ= 9.18 (Ar-OH*, s, [f], exp 2.00H, th 2.00H).

^13^C NMR (DMSO-d_6_, 600 MHz, 298°K): δ (ppm)= 14.0 [1]; 19.1 [2]; 27.7 [c]; 30.4 [a]; 30.6 [3]; 36.7 [b]; 44.3 [i]; 64.0 [4]; 115.2 [e]; 128.2 [d]; 139.6 [g]; 155.5 [h]; 173.6 [j].

- *Me-PA-mea*

**Figure S9** a) ^1^H and b) ^13^C NMR spectra of Me-PA-mea.

**a) b)**


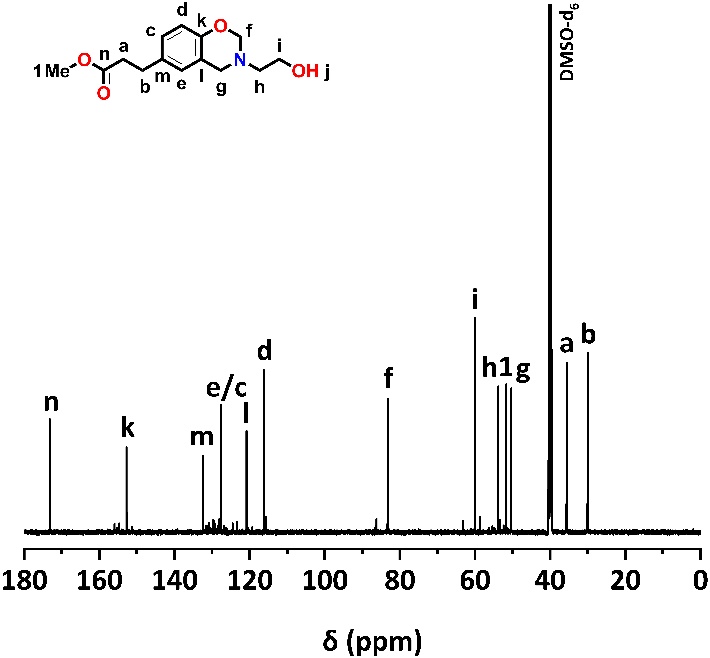

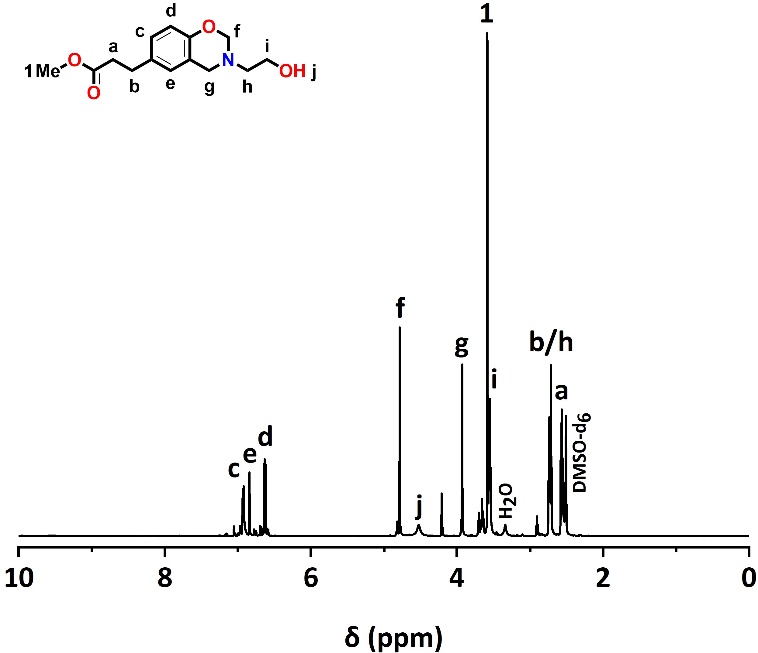


^1^H NMR (DMSO-d_6_, 600 MHz, 298°K): δ (ppm)= (assignment, multiplicity (coupling constant), [attribution], experimental integration, theoretical integration). δ= 2.57 (CH_2_-CH_2_*-C=O, t (J = 7.69 Hz), [a], exp 2.00H, th 2.00H); δ= 2.72 (CH_2_-CH_2_*-Ar & CH_2_-CH_2_*-N, m, [b&h], exp 3.73H, th 4.00H); δ= 3.55-3.58 (CH_3_*-C=O & CH_2_-CH_2_*-OH, [1&i], exp 4.98H, th 5.00H); δ= 3.93 (Ar-CH_2_*-N-, s, [g], exp 1.49H, th 2.00H); δ= 4.52 (CH_2_-OH*, s, [j], exp 0.79H, th 1.00H); δ= 4.78 (O-CH_2_*-N, s, [f], exp 1.50H, th 2.00H); δ= 6.62-6.93 (CH=CH*-C-O & C-CH*=C & C-CH*=CH, m, [d&e&c], exp 2.94H, th 3.00H).

^13^C NMR (DMSO-d_6_, 600 MHz, 298°K): δ (ppm)= 30.1 [b]; 35.7 [a]; 50.5 [g]; 51.8 [1]; 54.0 [h]; 60.1 [i]; 83.2 [f]; 116.3 [d]; 120.9 [l]; 127.7 [c]; 127.8 [e]; 132.6 [m]; 152.8 [k]; 173.3 [n].

-
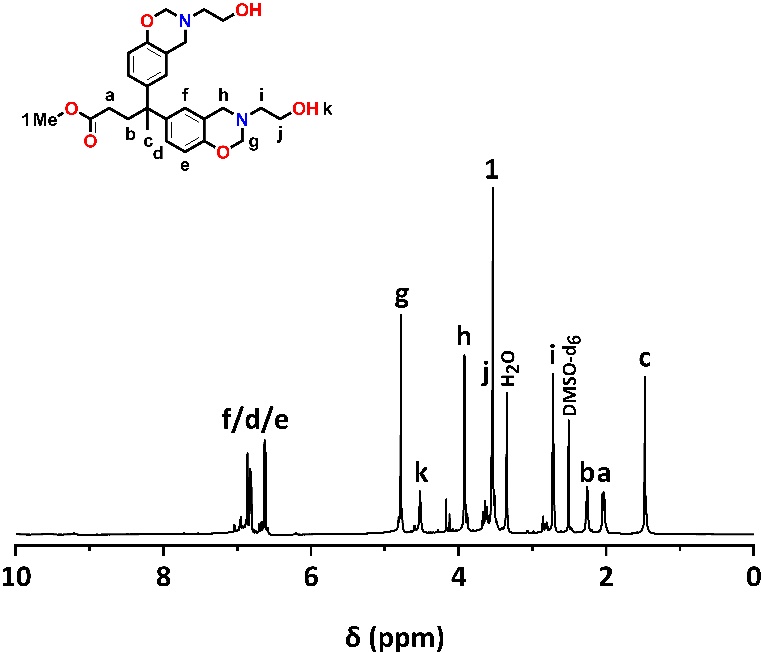
*Me-DPA-mea*

**Figure S10** a) ^1^H and b) ^13^C NMR spectra of Me-DPA-mea.

**a) b)**


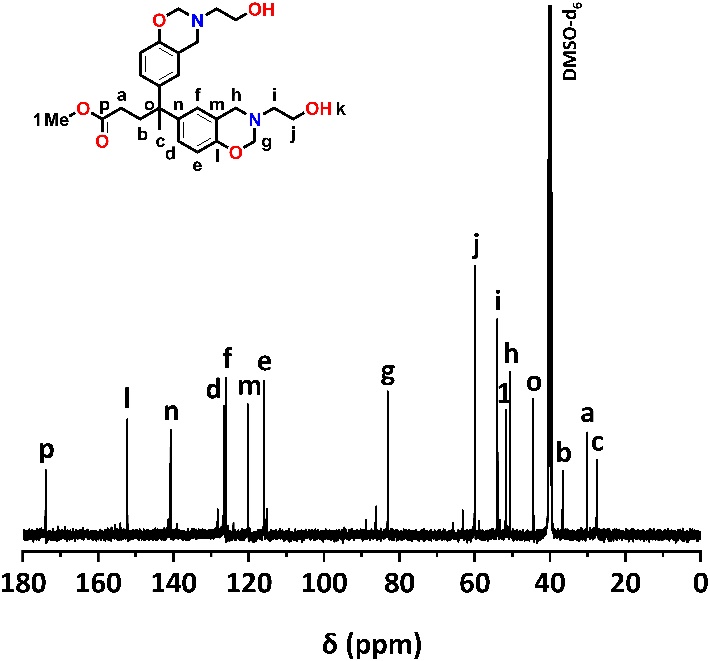


^1^H NMR (DMSO-d_6_, 600 MHz, 298°K): δ (ppm)= (assignment, multiplicity (coupling constant), [attribution], experimental integration, theoretical integration). δ= 1.48 (CH_3_*-C, s, [c], exp 3.00H, th 3.00H); δ= 2.03 (CH_2_-CH_2_*-C=O, t (J = 7.80 Hz), [a], exp 1.94H, th 2.00H); δ= 2.26 (CH_2_-CH_2_*-C, t (J = 7.95 Hz), [b], exp 1.95H, th 2.00H); δ= 2.72 (CH_2_-CH_2_*-N, t (J = 6.18 Hz), [i], exp 2.94H, th 4.00H); δ= 3.54 (CH_3_*-O-C=O, m, [1&j], exp 6.35H, th 7.00H); δ= 3.92 (Ar-CH_2_*-N, s, [h], exp 3.14H, th 4.00H); δ= 4.52 (CH_2_-OH*, m, [k], exp 1.50H, th 2.00H); δ= 4.78 (O-CH_2_*-N, s, [g], exp 3.21H, th 4.00H); δ= 6.62-6.86 (CH=CH*-C-O & C-CH*=CH & C-CH*=C, m, [e&d&f], exp 5.80H, th 6.00H).

^13^C NMR (DMSO-d_6_, 600 MHz, 298°K): δ (ppm)= 27.5 [c]; 30.1 [a]; 36.6 [b]; 44.5 [o]; 50.6 [h]; 51.8 [1]; 54.0 [i]; 60.0 [j]; 83.1 [g]; 115.9 [e]; 120.3 [m]; 126.0 [f]; 126.6 [d]; 140.8 [n]; 152.3 [l]; 173.9 [p].

- *Et-PA-mea*

**Figure S11** a) ^1^H and b) ^13^C NMR spectra of Et-PA-mea.

**a) b)**


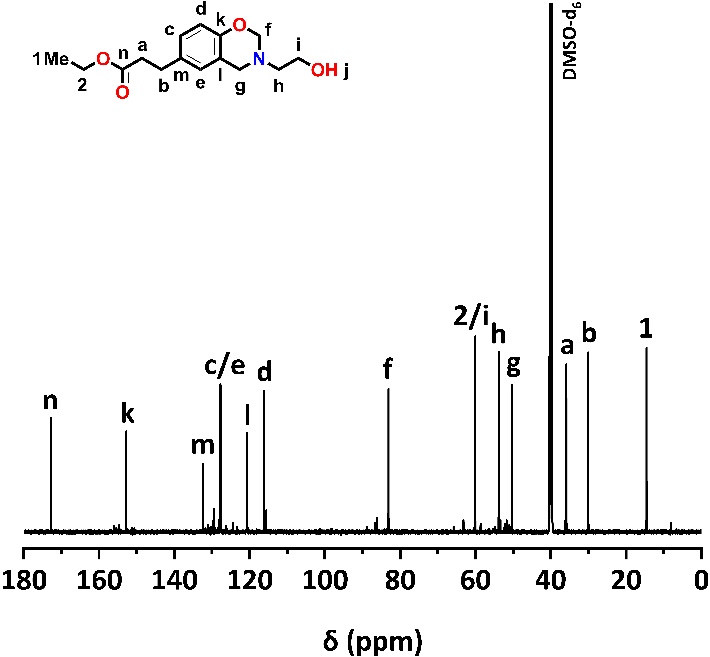

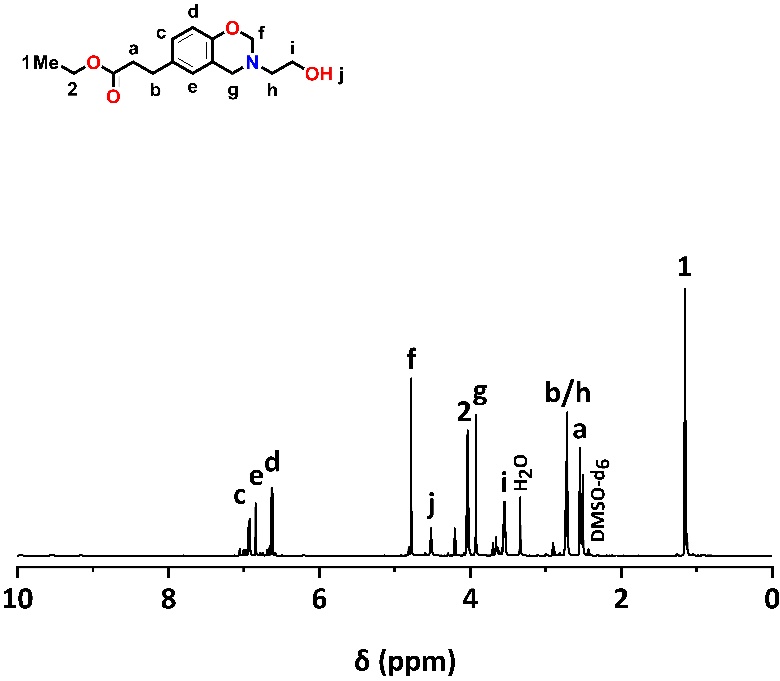


^1^H NMR (DMSO-d_6_, 600 MHz, 298°K): δ (ppm)= (assignment, multiplicity (coupling constant), [attribution], experimental integration, theoretical integration). δ= 1.16 (CH_3_*-CH_2_, t (J = 7.16 Hz), [1], exp 3.00H, th 3.00H); δ= 2.54 (CH_2_-CH_2_*-C=O, t (J = 7.58 Hz), [a], exp 2.02H, th 2.00H); δ= 2.73 (CH_2_-CH_2_*-Ar & CH_2_-CH_2_*-N, m, [b&h], exp 3.69H, th 4.00H); δ= 3.55 (CH_2_-CH_2_*-OH, m, [i], exp 1.78H, th 2.00H); δ= 3.92 (Ar-CH_2_*-N-, s, [g], exp 1.60H, th 2.00H); δ= 4.04 (CH_3_-CH_2_*-O, quad (J = 7.10 Hz), [2], exp 2.04H, th 2.00H); δ= 4.52 (CH_2_-OH*, s, [j], exp 0.84H, th 1.00H); δ= 4.78 (O-CH_2_*-N, s, [f], exp 1.60H, th 2.00H); δ= 6.62-6.94 (CH=CH*-C-O & C-CH*=C & C-CH*=CH, m, [d&e&c], exp 3.00H, th 3.00H).

^13^C NMR (DMSO-d_6_, 600 MHz, 298°K): δ (ppm)= 14.6 [1]; 30.1 [b]; 35.8 [a]; 50.3 [g]; 53.8 [h]; 60.0 [i]; 60.2 [2]; 83.1 [f]; 116.1 [d]; 120.8 [l]; 127.6 [e]; 127.7 [c]; 132.5 [m]; 152.7 [k]; 172.7 [n].

- *Et-DPA-mea*

**Figure S12** a) ^1^H and b) ^13^C NMR spectra of Et-DPA-mea.

**a) b)**


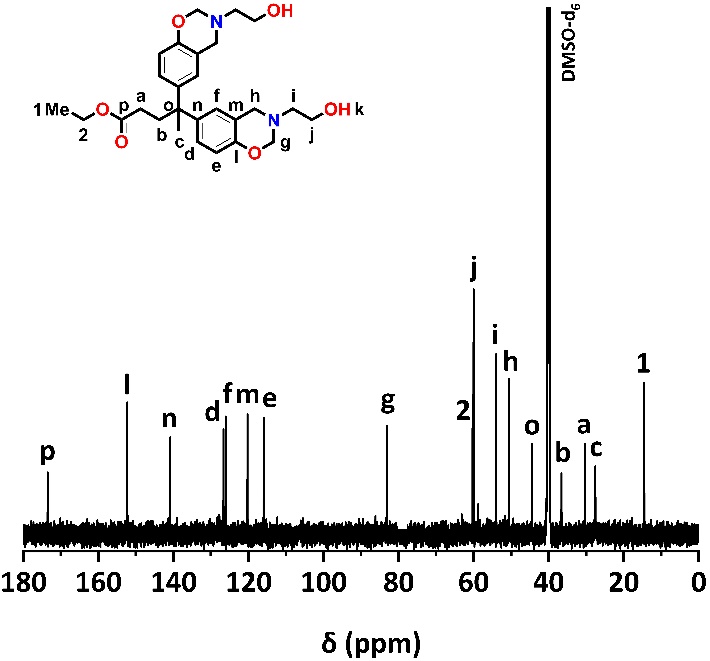

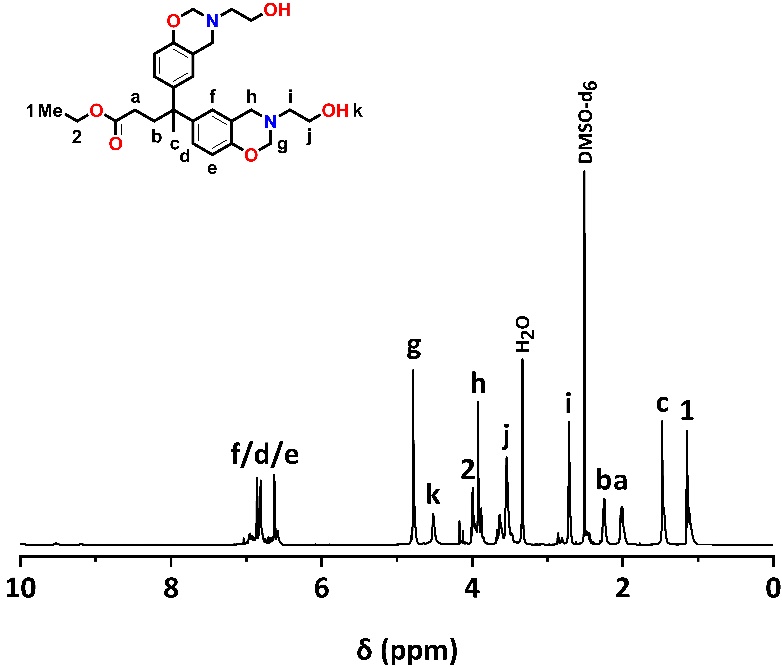


^1^H NMR (DMSO-d_6_, 600 MHz, 298°K): δ (ppm)= (assignment, multiplicity (coupling constant), [attribution], experimental integration, theoretical integration). δ= 1.14 (CH_3_*-CH_2_, t (J = 7.10 Hz), [1], exp 3.00H, th 3.00H); δ= 1.48 (CH_3_*-C, s, [c], exp 3.00H, th 3.00H); δ= 2.02 (CH_2_-CH_2_*-C=O, t (J = 7.80 Hz), [a], exp 1.98H, th 2.00H); δ= 2.25 (CH_2_-CH_2_*-C, t (J = 8.20 Hz), [b], exp 1.99H, th 2.00H); δ= 2.72 (CH_2_-CH_2_*-N, t (J = 6.10 Hz), [i], exp 3.16H, th 4.00H); δ= 3.54 (CH_2_-CH_2_*-OH, m, [j], exp 3.80H, th 4.00H); δ= 3.92 (Ar-CH_2_*-N, s, [h], exp 3.03H, th 4.00H); δ= 4.00 (CH_3_-CH_2_*-O, quad (J= 7.16 Hz), [2], exp 2.01H, th 2.00H); δ= 4.52 (CH_2_-OH*, s, [k], exp 1.63H, th 2.00H); δ= 4.78 (O-CH_2_*-N, s, [g], exp 3.17H, th 4.00H); δ= 6.63-6.86 (CH=CH*-C-O & C-CH*=CH & C-CH*=C, m, [e&d&f], exp 5.99H, th 6.00H).

^13^C NMR (DMSO-d_6_, 600 MHz, 298°K): δ (ppm)= 14.5 [1]; 27.5 [c]; 30.3 [a]; 36.6 [b]; 44.5 [o]; 50.6 [h]; 54.0 [i]; 60.0 [j]; 60.3[2]; 83.1 [g]; 115.9 [e]; 120.3 [m]; 126.0 [f]; 126.6 [d]; 140.8 [n]; 152.3 [l]; 173.5 [p].

- *Pr-PA-mea*

**Figure S13** a) ^1^H and b) ^13^C NMR spectra of Pr-PA-mea.

**a) b)**


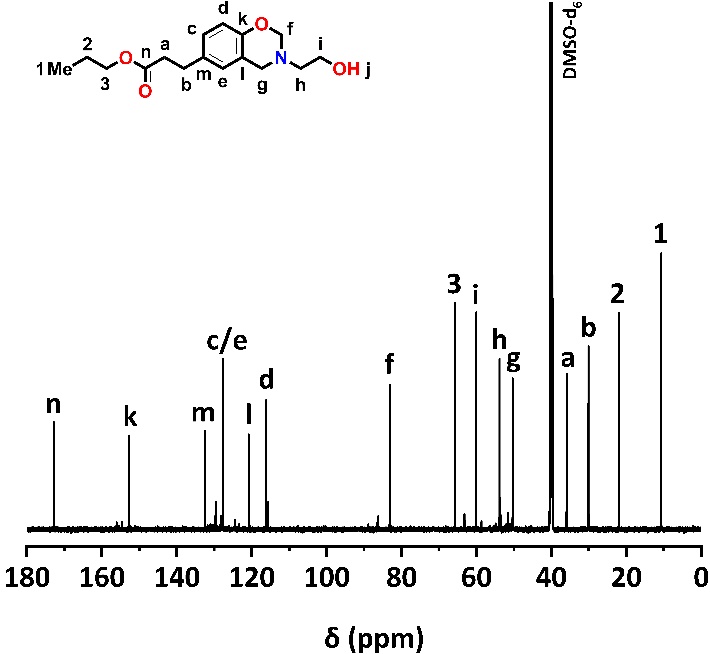

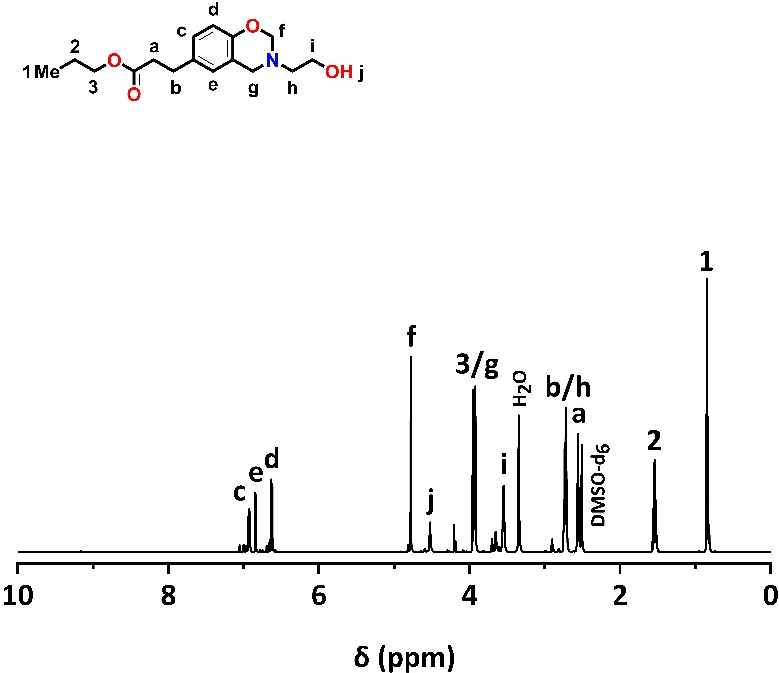


^1^H NMR (DMSO-d_6_, 600 MHz, 298°K): δ (ppm)= (assignment, multiplicity (coupling constant), [attribution], experimental integration, theoretical integration). δ= 0.85 (CH_3_*-CH_2_, t (J = 7.43 Hz), [1], exp 3.00H, th 3.00H); δ= 1.54 (CH_3_-CH_2_*-CH_2_, sex (J = 7.31 Hz), [2], exp 2.00H, th 2.00H); δ= 2.56 (CH_2_-CH_2_*-C=O, t (J = 7.61 Hz), [a], exp 2.02H, th 2.00H); δ= 2.72 (CH_2_-CH_2_*-Ar & CH_2_-CH_2_*-N, m, [b&h], exp 3.59H, th 4.00H); δ= 3.54 (CH_2_-CH_2_*-OH, t (J = 5.44 Hz), [i], exp 1.87H, th 2.00H); δ= 3.92 (Ar-CH_2_*-N-, s, [g], exp 1.61H, th 2.00H); δ= 3.95 (CH_2_-CH_2_*-O, t (J = 6.63 Hz), [3], exp 2.01H, th 2.00H); δ= 4.52 (CH_2_-OH*, s, [j], exp 0.84H, th 1.00H); δ= 4.78 (O-CH_2_*-N, s, [f], exp 1.60H, th 2.00H); δ= 6.62-6.94 (CH=CH*-C-O & C-CH*=C & C-CH*=CH, m, [d&e&c], exp 3.01H, th 3.00H).

^13^C NMR (DMSO-d_6_, 600 MHz, 298°K): δ (ppm)= 10.7 [1]; 22.0 [2]; 30.1 [b]; 35.8 [a]; 50.3 [g]; 53.8 [h]; 60.0 [i]; 65.7 [3]; 83.1 [f]; 116.1 [d]; 120.7 [l]; 127.6 [e]; 127.7 [c]; 132.4 [m]; 152.7 [k]; 172.8 [n].

- *Pr-DPA-mea*

**Figure S14** a) ^1^H and b) ^13^C NMR spectra of Pr-DPA-mea.

**a) b)**


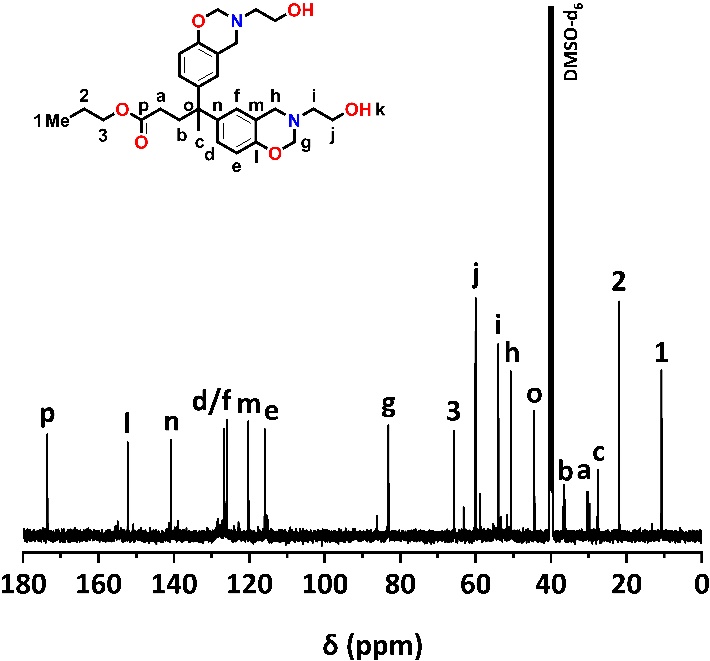

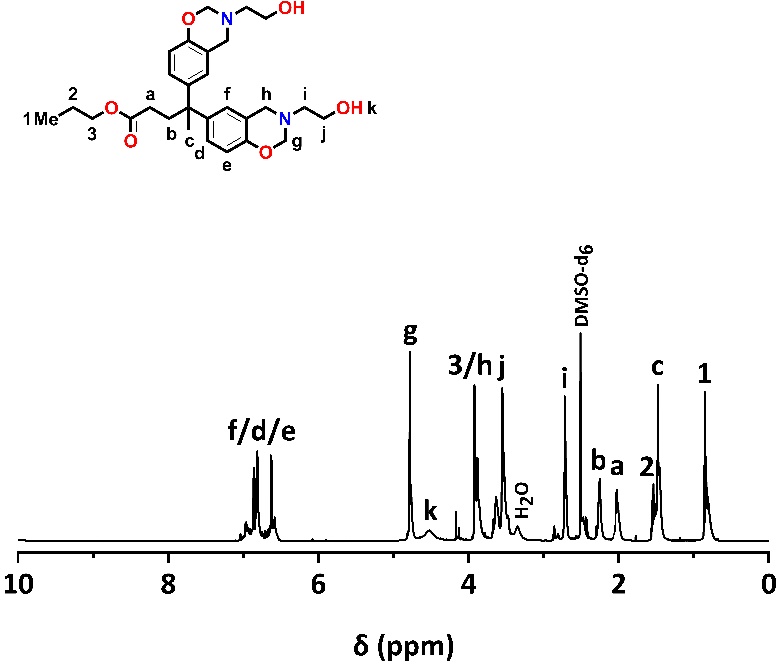


^1^H NMR (DMSO-d6, 600 MHz, 298 °K): δ (ppm) = (assignment, multiplicity (coupling constant), [attribution], experimental integration, theoretical integration). δ= 0.86 (CH_3_*-CH_2_, t (J = 7.41 Hz), [1], exp 3.01H, th 3.00H); δ= 1.48-1.54 (CH_3_*-C & CH_3_-CH_2_*-CH_2_-O, m, [c&2], exp 5.00H, th 5.00H); δ= 2.03 (CH_2_-CH_2_*-C=O, t (J = 8.10 Hz), [a], exp 2.00H, th 2.00H); δ= 2.26 (CH_2_-CH_2_*-C, t (J = 7.98 Hz), [b], exp 1.98H, th 2.00H); δ= 2.72 (CH_2_-CH_2_*-N, t (J = 6.13 Hz), [i], exp 3.00H, th 4.00H); δ= 3.55 (CH_2_-CH_2_*-OH, m, [j], exp 3.72H, th 4.00H); δ= 3.88-3.92 (Ar-CH_2_*-N & CH_2_-CH_2_*-O, m, [h&3], exp 5.08H, th 6.00H); δ= 4.53 (CH_2_-OH*, s, [k], exp 1.52H, th 2.00H); δ= 4.78 (O-CH_2_*-N, s, [g], exp 3.12H, th 4.00H); δ= 6.63-6.87 (CH=CH*-C-O & C-CH*=CH & C-CH*=C, m, [e&d&f], exp 5.76H, th 6.00H).

^13^C NMR (DMSO-d_6_, 600 MHz, 298°K): δ (ppm)= 10.7 [1]; 21.9 [2]; 27.5 [c]; 30.3 [a]; 36.7 [b]; 44.5 [o]; 50.6 [h]; 54.0 [i]; 60.0 [j]; 65.8 [3]; 83.1 [g]; 115.9 [e]; 120.3 [m]; 126.0 [f]; 126.6 [d]; 140.8 [n]; 152.3 [l]; 173.6 [p].

- *Bu-PA-mea*

**Figure S15** a) ^1^H and b) ^13^C NMR spectra of Bu-PA-mea.

**a) b)**


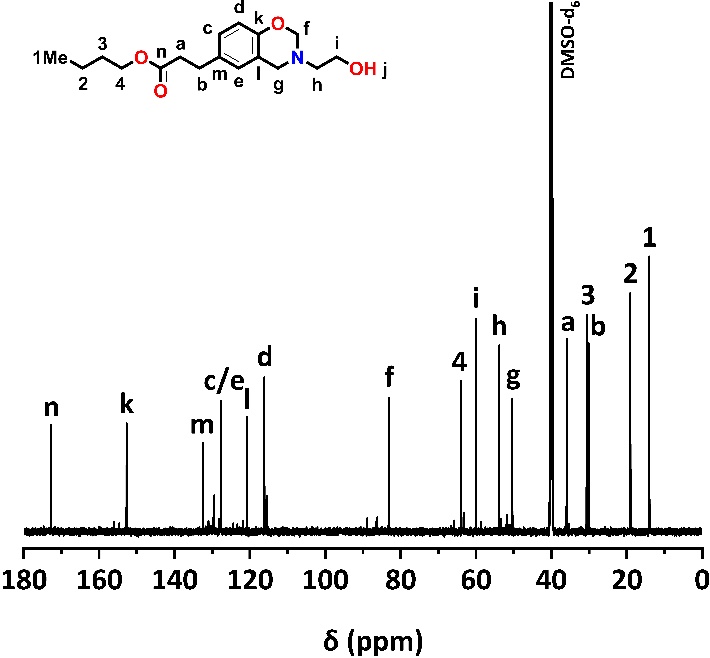

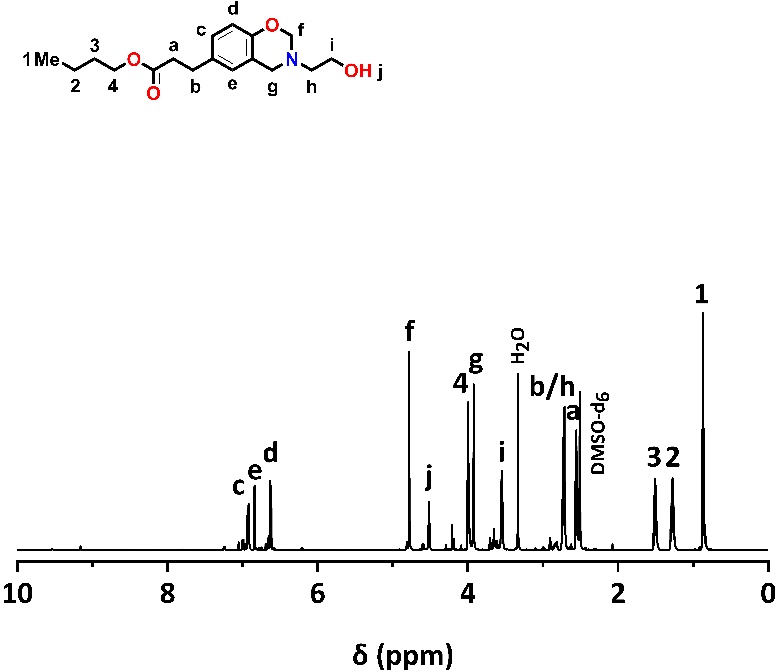


^1^H NMR (DMSO-d_6_, 600 MHz, 298°K): δ (ppm)= (assignment, multiplicity (coupling constant), [attribution], experimental integration, theoretical integration). δ= 0.87 (CH_3_*-CH_2_, t (J = 7.41 Hz), [1], exp 3.00H, th 3.00H); δ= 1.27 (CH_3_-CH_2_*-CH_2_, sex ( J= 7.41 Hz), [2], exp 2.00H, th 2.00H); δ= 1.51 (CH_2_-CH_2_*-CH_2_-O, quint. (J = 7.41 Hz), [3], exp 2.00H, th 2.00H); δ= 2.55 (CH_2_-CH_2_*-C=O, t (J = 7.60 Hz), [a], exp 1.98H, th 2.00H); δ= 2.72 (CH_2_-CH_2_*-Ar & CH_2_-CH_2_*-N, m, [b&h], exp 3.48H, th 4.00H); δ= 3.55 (CH_2_-CH_2_*-OH, quad (J = 5.70 Hz), [i], exp 1.75H, th 2.00H); δ= 3.92 (Ar-CH_2_*-N-, s, [g], exp 1.62H, th 2.00H); δ= 4.00 (CH_2_-CH_2_*-O, t (J = 6.65 Hz), [4], exp 1.97H, th 2.00H); δ= 4.52 (CH_2_-OH*, s, [j], exp 0.76H, th 1.00H); δ= 4.78 (O-CH_2_*-N, s, [f], exp 1.57H, th 2.00H); δ= 6.62-6.93 (CH=CH*-C-O & C-CH*=C & C-CH*=CH, m, [d&e&c], exp 3.05H, th 3.00H).

^13^C NMR (DMSO-d_6_, 600 MHz, 298°K): δ (ppm)= 14.0 [1]; 19.0 [2]; 30.1 [b]; 30.7 [3]; 35.8 [a]; 50.4 [g]; 53.9 [h]; 60.0 [i]; 63.9 [4]; 83.1 [f]; 116.1 [d]; 120.7 [l]; 127.6 [e]; 127.7 [c]; 132.4 [m]; 152.7 [k]; 172.8 [n].

- *Bu-DPA-mea*

**Figure S16** a) ^1^H and b) ^13^C NMR spectra of Bu-DPA-mea.

**a) b)**


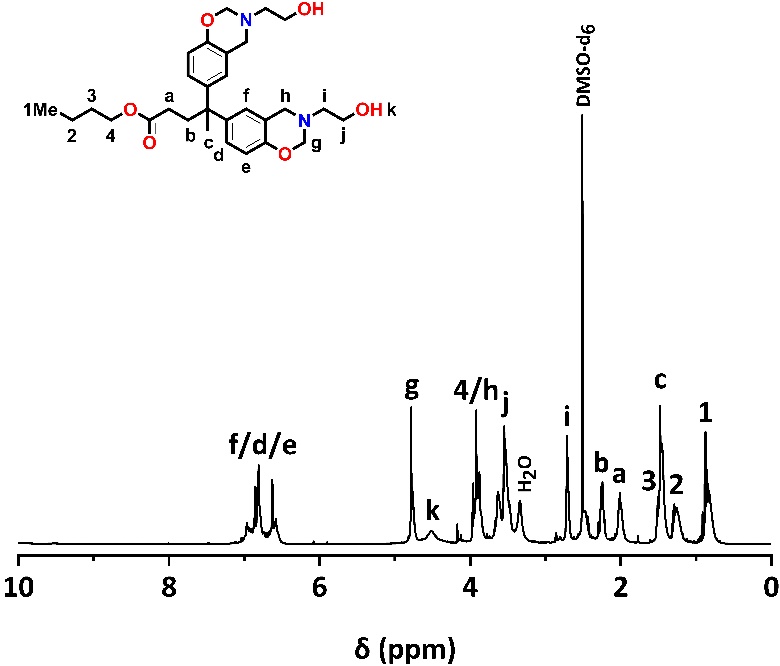

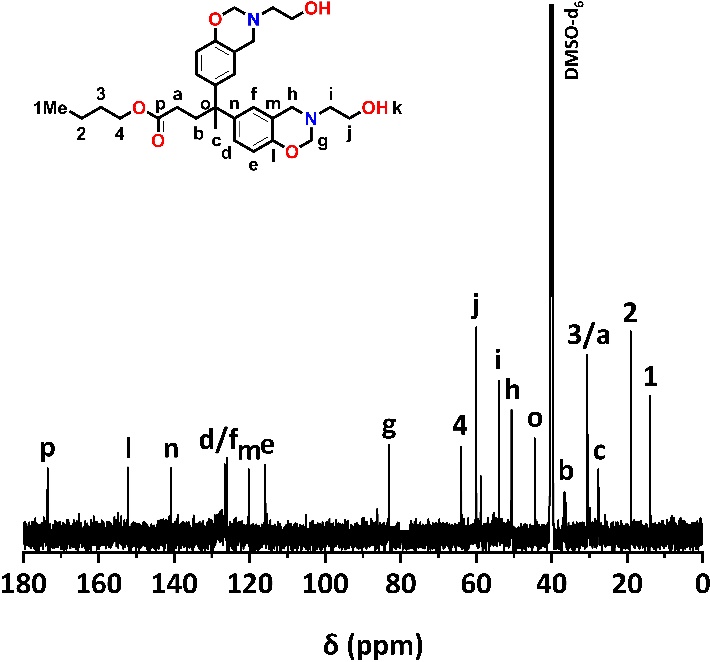


^1^H NMR (DMSO-d_6_, 600 MHz, 298 °K): δ (ppm) = (assignment, multiplicity (coupling constant), [attribution], experimental integration, theoretical integration). δ= 0.87 (CH_3_*-CH_2_, t (J = 7.39 Hz), [1], exp 3.05H, th 3.00H); δ= 1.29 (CH_3_-CH_2_*-CH_2_, m, [2], exp 2.03H, th 2.00H); δ= 1.47-1.52 (CH_3_*-C & CH_2_-CH_2_*-CH_2_-O, m, [c&3], exp 5.00H, th 5.00H); δ= 2.03 (CH_2_-CH_2_*-C=O, t (J = 8.11 Hz), [a], exp 1.97H, th 2.00H); δ= 2.26 (CH_2_-CH_2_*-C, t (J = 7.97 Hz), [b], exp 2.00H, th 2.00H); δ= 2.72 (CH_2_-CH_2_*-N, t (J = 6.26 Hz), [i], exp 3.02H, th 4.00H); δ= 3.55 (CH_2_-CH_2_*-OH, m, [j], exp 3.67H, th 4.00H); δ= 3.92 (Ar-CH_2_*-N & CH_2_-CH_2_*-O, m, [h&4], exp 5.07H, th 6.00H); δ= 4.52 (CH_2_-OH*, s, [k], exp 1.49H, th 2.00H); δ= 4.78 (O-CH_2_*-N, s, [g], exp 3.15H, th 4.00H); δ= 6.58-6.86 (CH=CH*-C-O & C-CH*=CH & C-CH*=C, m, [e&d&f], exp 6.02H, th 6.00H).

^13^C NMR (DMSO-d_6_, 600 MHz, 298°K): δ (ppm) = 14.0 [1]; 19.1 [2]; 27.5 [c]; 30.3 [a]; 30.6 [3] 36.7 [b]; 44.5 [o]; 50.6 [h]; 54.0 [i]; 60.0 [j]; 64.0 [4]; 83.1 [g]; 115.9 [e]; 120.3 [m]; 126.0 [f]; 126.6 [d]; 140.8 [n]; 152.3 [l]; 173.6 [p].

**Table S3** Elemental composition of alkylester-based benzoxazine precursors (theoretical composition).

| Molecule | C (%) | H (%) | N (%) | O (%) |
| --- | --- | --- | --- | --- |
| Me-PA-mea | 63.5 ± 0.2 (63.4) | 7.1 ± 0.2 (7.2) | 5.4 ± 0.0 (5.3) | 24.0 ± 0.3 (24.1) |
| Et-PA-mea | 64.4 ± 0.2 (64.5) | 7.8 ± 0.3 (7.6) | 5.2 ± 0.1 (5.0) | 22.6 ± 0.5 (22.9) |
| Pr-PA-mea | 65.3 ± 0.1 (65.5) | 7.8 ± 0.4 (7.9) | 4.7 ± 0.0 (4.8) | 22.2 ± 0.4 (21.8) |
| Bu-PA-mea | 66.3 ± 0.1 (66.4) | 8.1 ± 0.4 (8.2) | 4.9 ± 0.0 (4.6) | 20.8 ± 0.5 (20.8) |
| Me-DPA-mea | 64.2 ± 0.0 (66.4) | 7.8 ± 0.4 (7.3) | 5.9 ± 0.1 (6.0) | 22.1 ± 0.4 (20.4) |
| Et-DPA-mea | 64.8 ± 0.1 (66.9) | 7.8 ± 0.0 (7.5) | 5.8 ± 0.1 (5.8) | 21.4 ± 0.2 (19.8) |
| Pr-DPA-mea | 67.1 ± 0.9 (67.4) | 8.4 ± 0.3 (7.7) | 5.8 ± 0.2 (5.6) | 18.7 ± 1.3 (19.3) |
| Bu-DPA-mea | 67.7 ± 0.0 (67.9) | 8.6 ± 0.1 (7.9) | 5.5 ± 0.0 (5.5) | 18.2 ± 0.1 (18.7) |

^a^ determined by subtracting other elements to the final composition of the precursor knowing that S < 0.5 %

#





**Figure S17** DSC thermograms of R-PA-mea benzoxazine precursors (10°C·min^-1^).

**Figure S18** DSC thermograms of R-DPA-mea benzoxazine precursors (10°C·min^-1^).

*Comment*: The experimental monitoring of polyester cross-linking through condensation and irreversible transesterification of alkylester bonds by TGA-µGC is limited by the detection of the mono-alcohol solvents. Physical limitations such as the rigidity of the cross-linked network or alcohol’s vapor pressure may underestimate the release of mono-alcohol solvents adduct of irreversible TER.


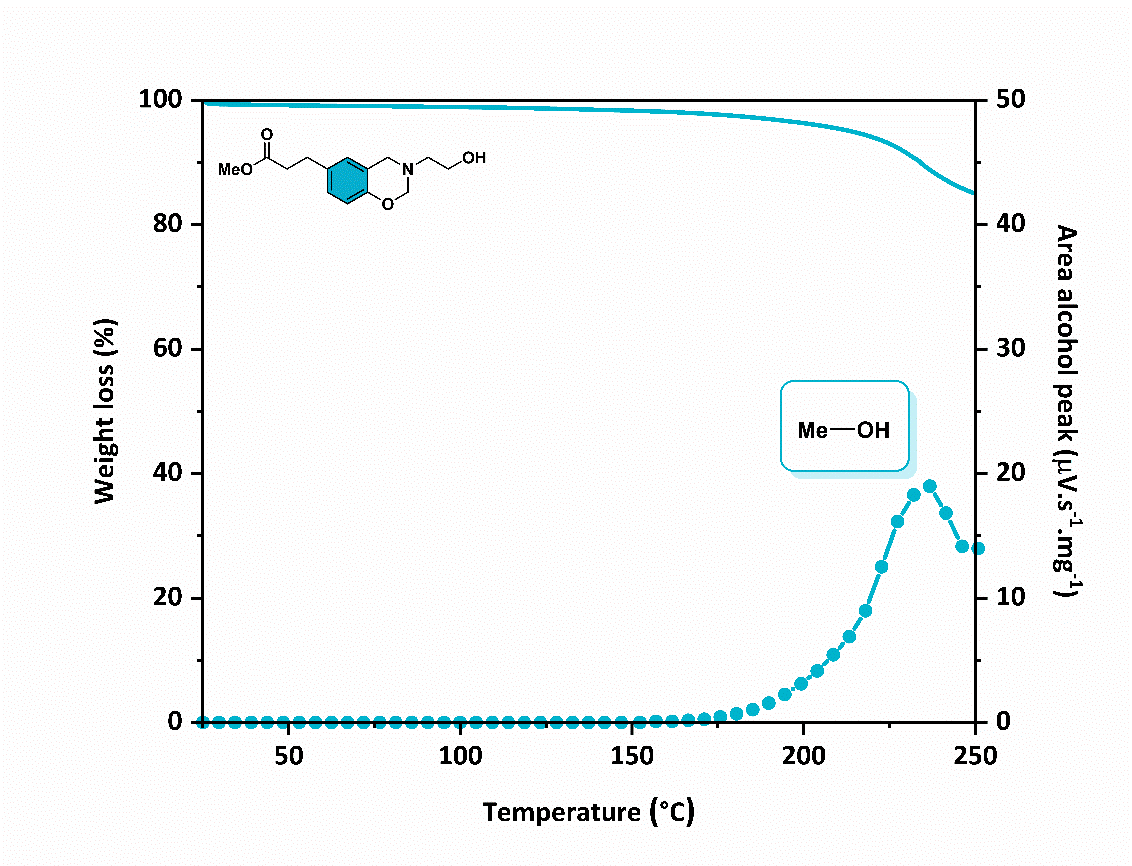


**Figure S19** TGA-µGC thermograms of Me-PA-mea (2°C·min^-1^).


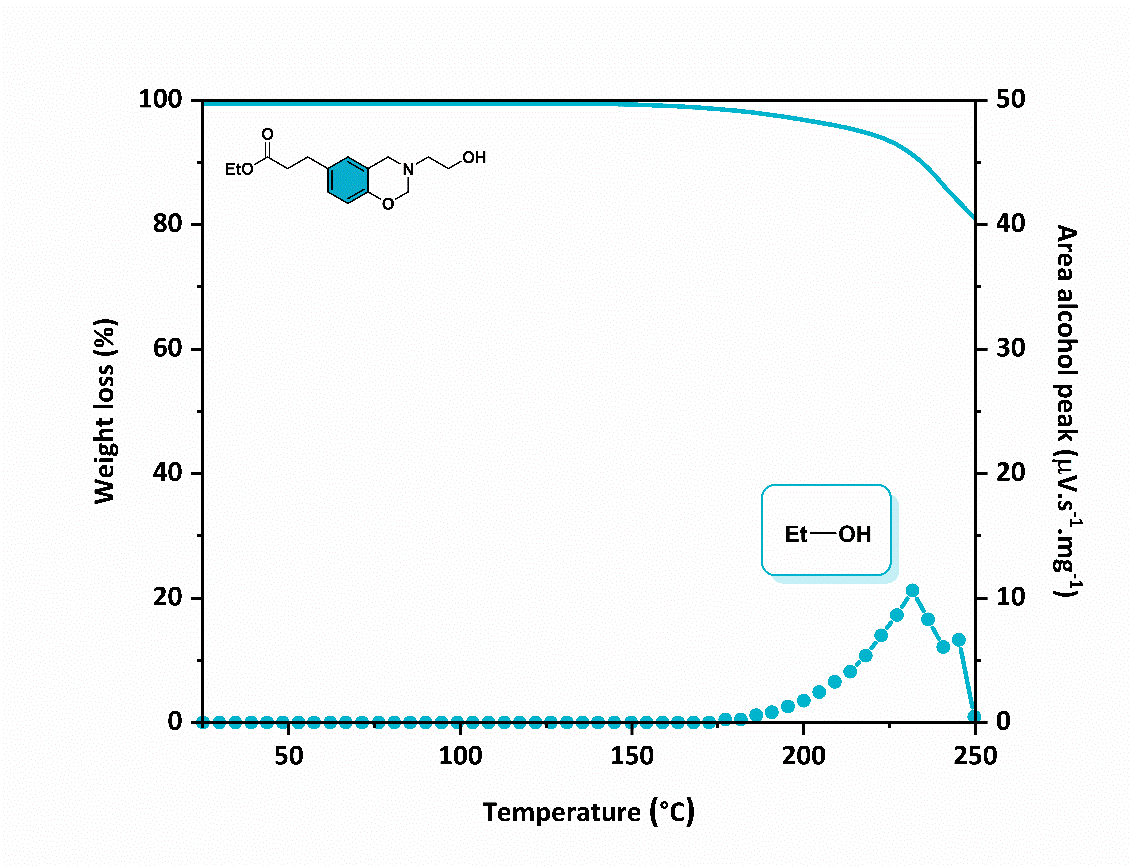


**Figure S20** TGA-µGC thermograms of Et-PA-mea (2°C·min^-1^).







**Figure S22** TGA-µGC thermograms of Bu-PA-mea (2°C·min^-1^).

**Figure S21** TGA-µGC thermograms of Pr-PA-mea (2°C·min^-1^).







**Figure S23** TGA-µGC thermograms of Me-DPA-mea (2°C·min^-1^).

**Figure S24** TGA-µGC thermograms of Et-DPA-mea (2°C·min^-1^).







**Figure S25** TGA-µGC thermograms of Pr-DPA-mea (2°C·min^-1^).

**Figure S26**  TGA-µGC thermograms of Bu-DPA-mea (2°C·min^-1^).







**Figure S27** Evolution of the complex viscosity of R-PA-mea as a function of time and temperature (2°C·min^-1^ heating rate).

**Figure S28** Evolution of the complex viscosity of R-DPA-mea as a function of time and temperature (2°C·min^-1^ heating rate).

**



**

**Figure S29** Isothermal evolution of the complex viscosity of R-PA-mea as a function of time at 180°C.

**Figure S30** Isothermal evolution of the complex viscosity of R-DPA-mea as a function of time at 160°C.







**Figure S32** Isothermal evolution of the viscosity of R-PA-mea as a function of the shear rate at 50°C.

**Figure S31** Evolution of the gelation time of R-PA-mea (160°C) and R-DPA-mea (180°C) as a function of the length of the alkylester side-chain.


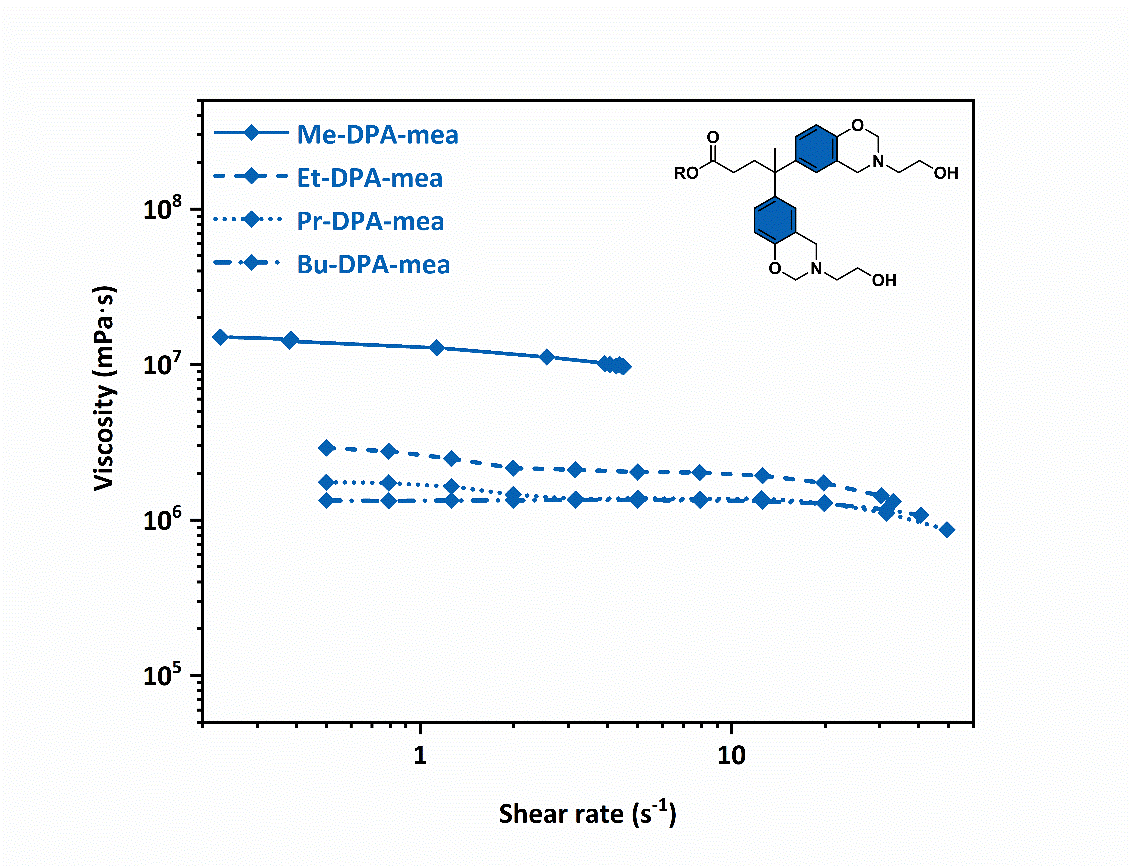


**Figure S33** Isothermal evolution of the viscosity of R-DPA-mea as a function of the shear rate at 50°C.

*Comment:* The viscosity of R-PA-mea and R-DPA-mea precursors gradually decreased from 10^3^ to 10^2^ mPa.s and from 10^7^ to 10^6^ mPa.s with increasing length of the alkylester side chain, from methyl to butyl, respectively. The lower viscosity of alkylester-based benzoxazine precursors increased the degree of mobility of reactive functional species, therefore increasing the probability of intermolecular activation promoting faster cross-linking reactions.

**Self-blown polybenzoxazine foams**


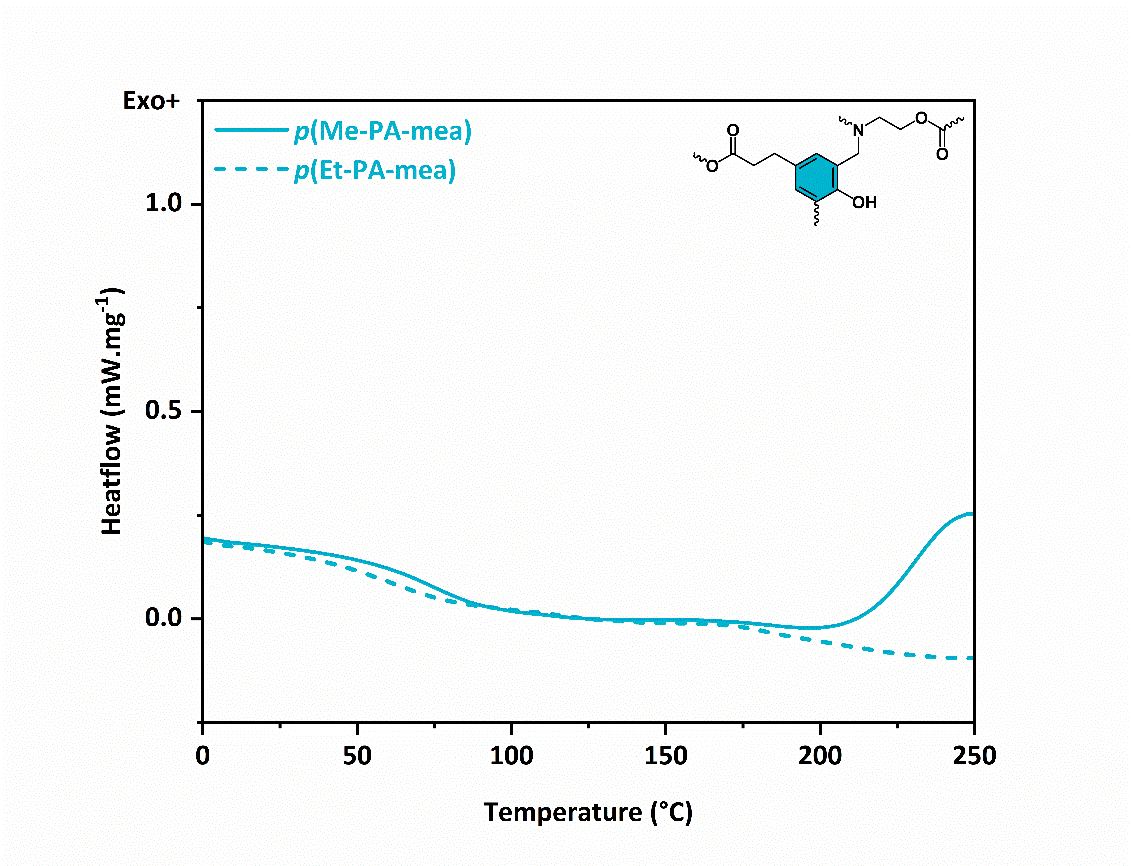


**Figure S35** DSC thermograms of p(R-PA-mea) self-blown polybenzoxazine foams (10°C·min^-1^).

**Figure S34** Optimization of the foaming process of Me-DPA-mea: evolution of the extent rate of irreversible transesterification and volumetric expansion as a function of the foaming time.


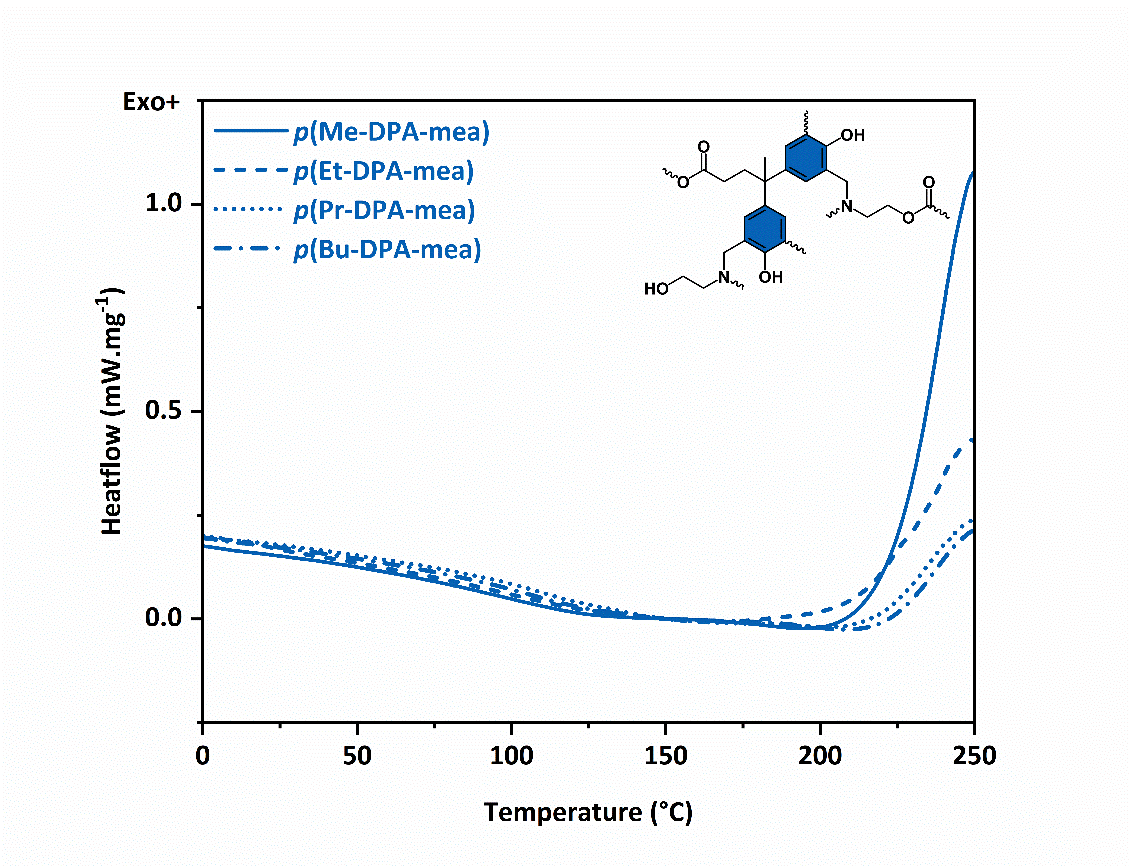


**Figure S36** DSC thermograms of p(R-DPA-mea) self-blown polybenzoxazine foams (10°C·min^-1^).

***
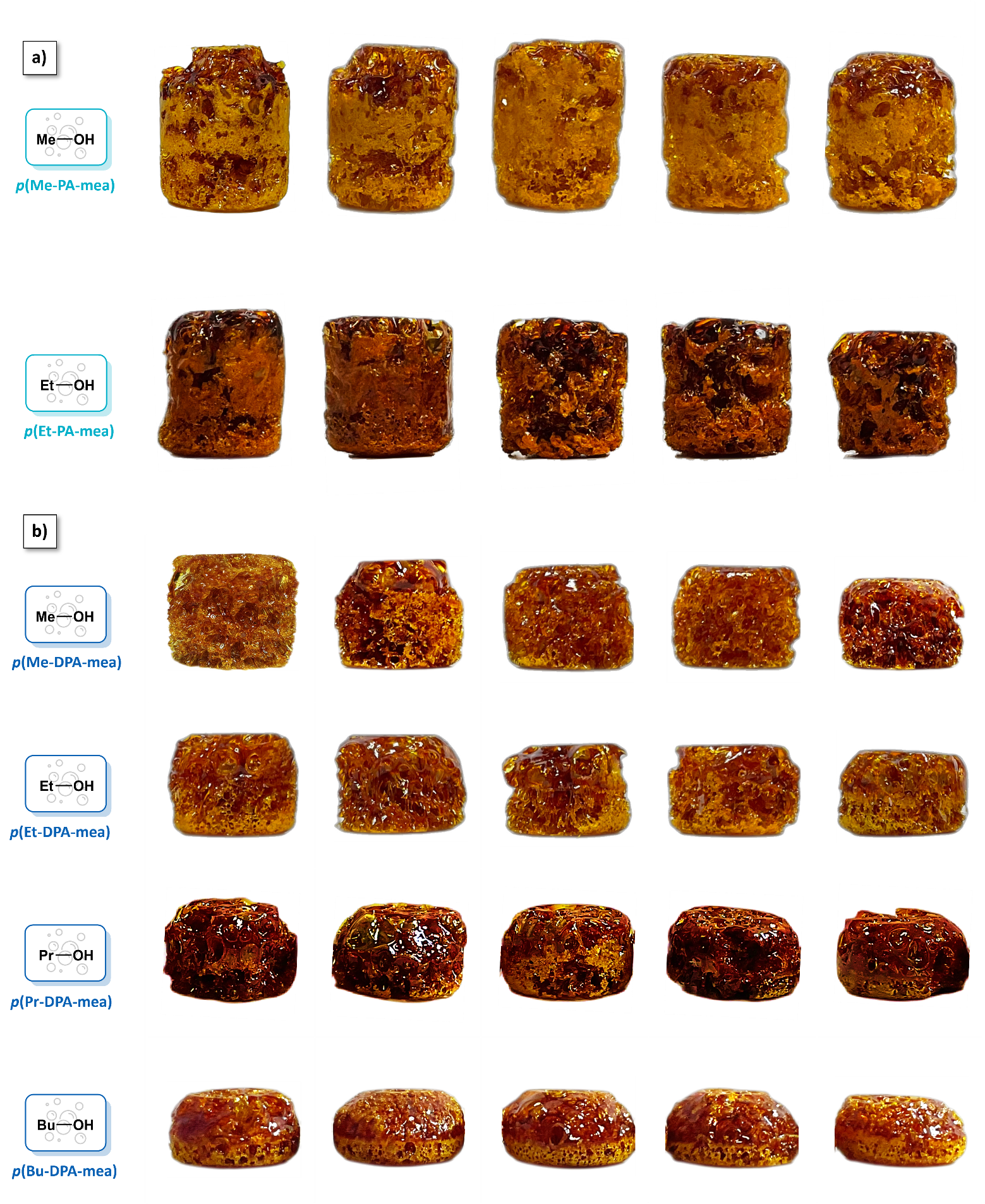
***

**Figure S37** Reproducibility of the foaming process of starting from a) R-PA-mea and b) R-DPA-mea alkylester-based benzoxazine precursors.


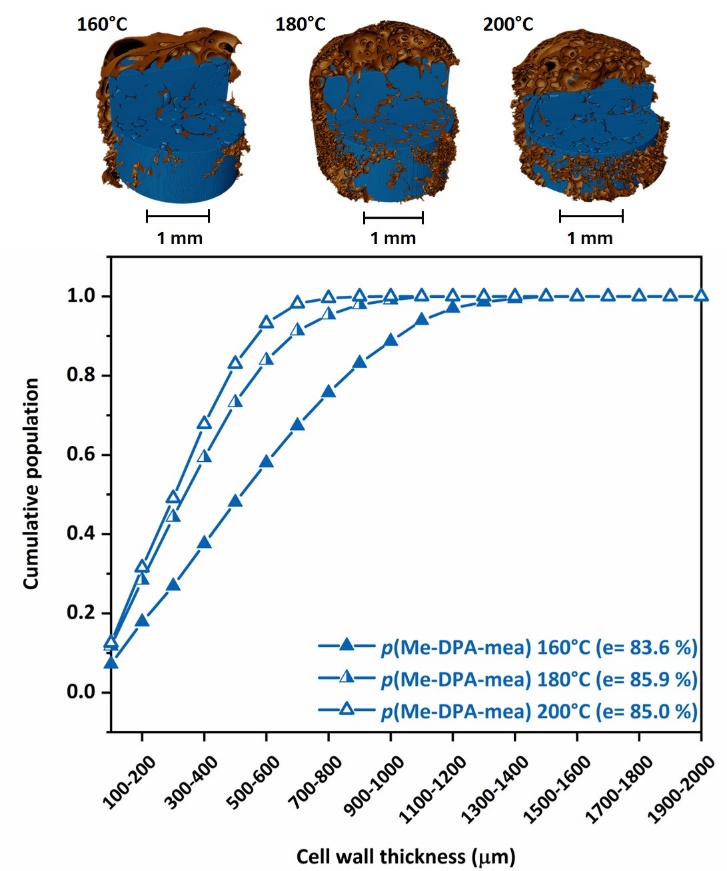


**Figure S39** Distribution of wall thickness of p(Me-DPA-mea) self-blown polybenzoxazine foam at different temperatures.

**Figure S38** Reproducibility of the foaming process of p(Me-PA-mea) and p(Me-DPA-mea) self-blown polybenzoxazine foams with different container’s volume.


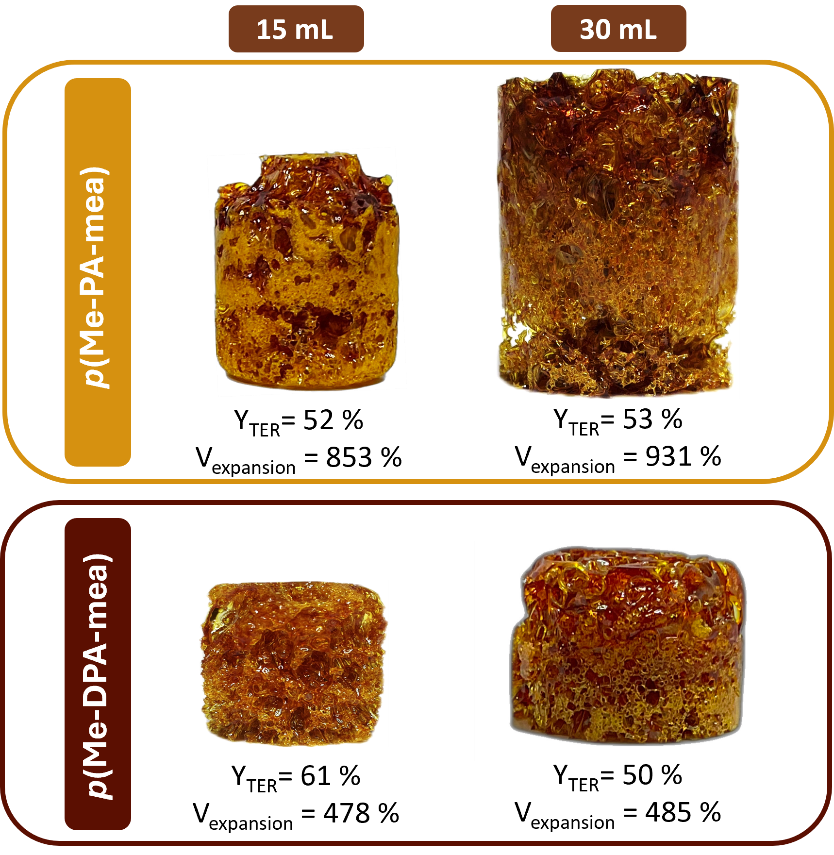


**Figure S40** Compressive strain-stress curves of p(R-PA-mea) self-blown polybenzoxazine foams (2 mm·min^-1^).





**Figure S41** Compressive strain-stress curves of p(R-DPA-mea) self-blown polybenzoxazine foams (2 mm·min^-1^).





**Figure S43** Dynamic TGA curves of p(R-DPA-mea) self-blown polybenzoxazine foams
(10°C·min^-1^, N_2_).







**Figure S42** Dynamic TGA curves of p(R-PA-mea) self-blown polybenzoxazine foams (10°C·min^-1^, N_2_).


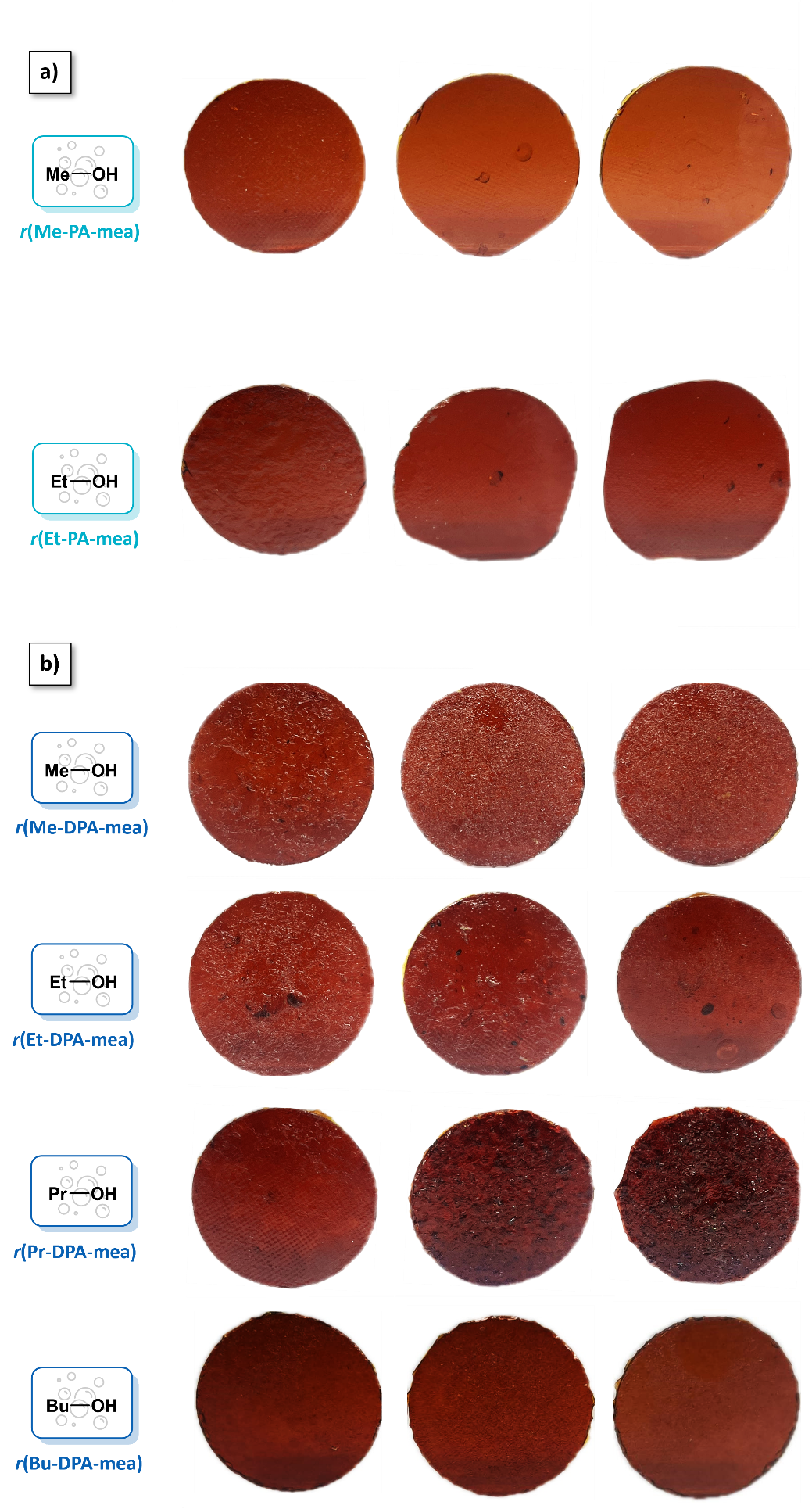


**Figure S44** Reproducibility of the mechanical reprocessing of starting a) r(R-PA-mea) and b) r(R-DPA-mea) reprocessed polybenzoxazine resins.

**Figure S46** Three-dimensional tomography images of reprocessed resins.


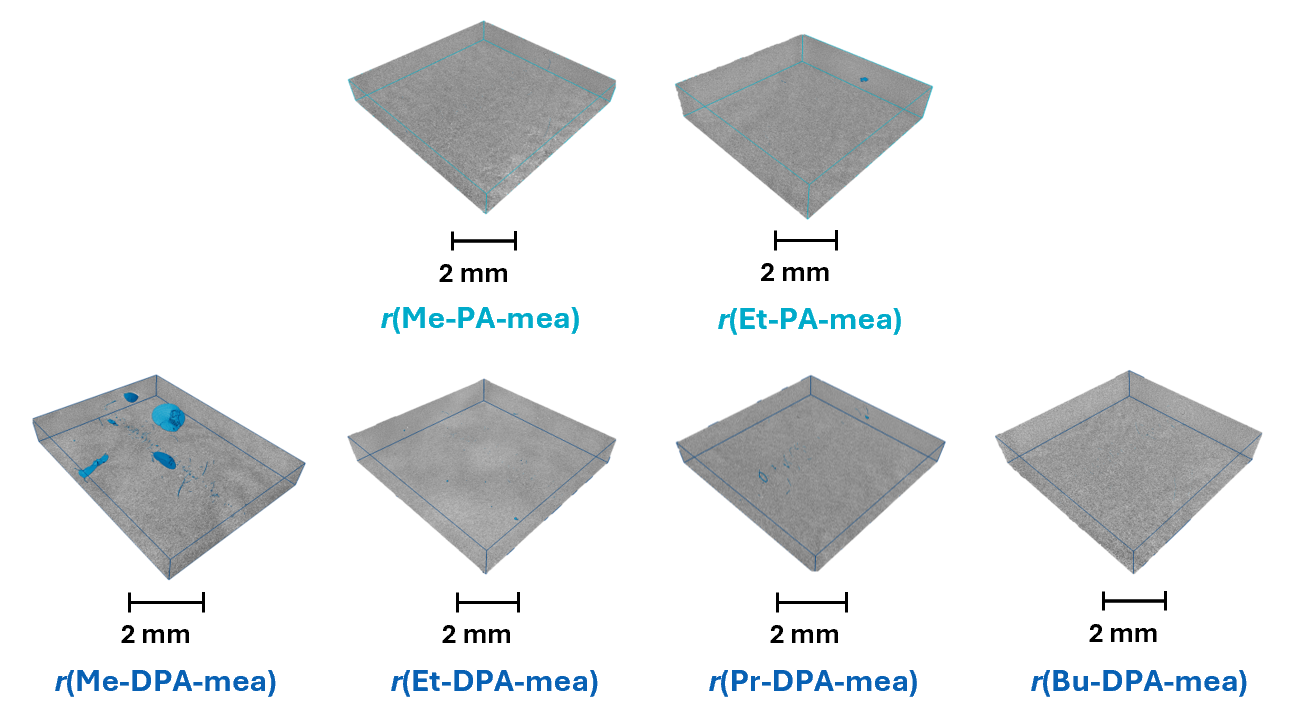


**Figure S45** Photographs of reprocessed (Me-DPA-mea) after one (R1), two (R2), and four (R4) mechanical reprocessing.


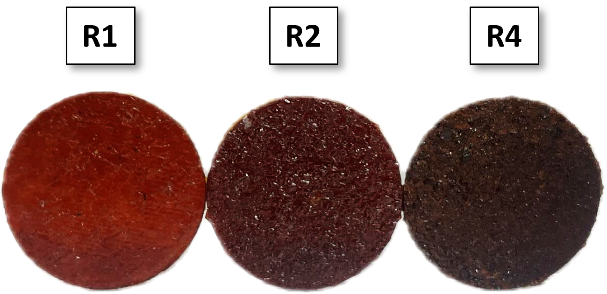

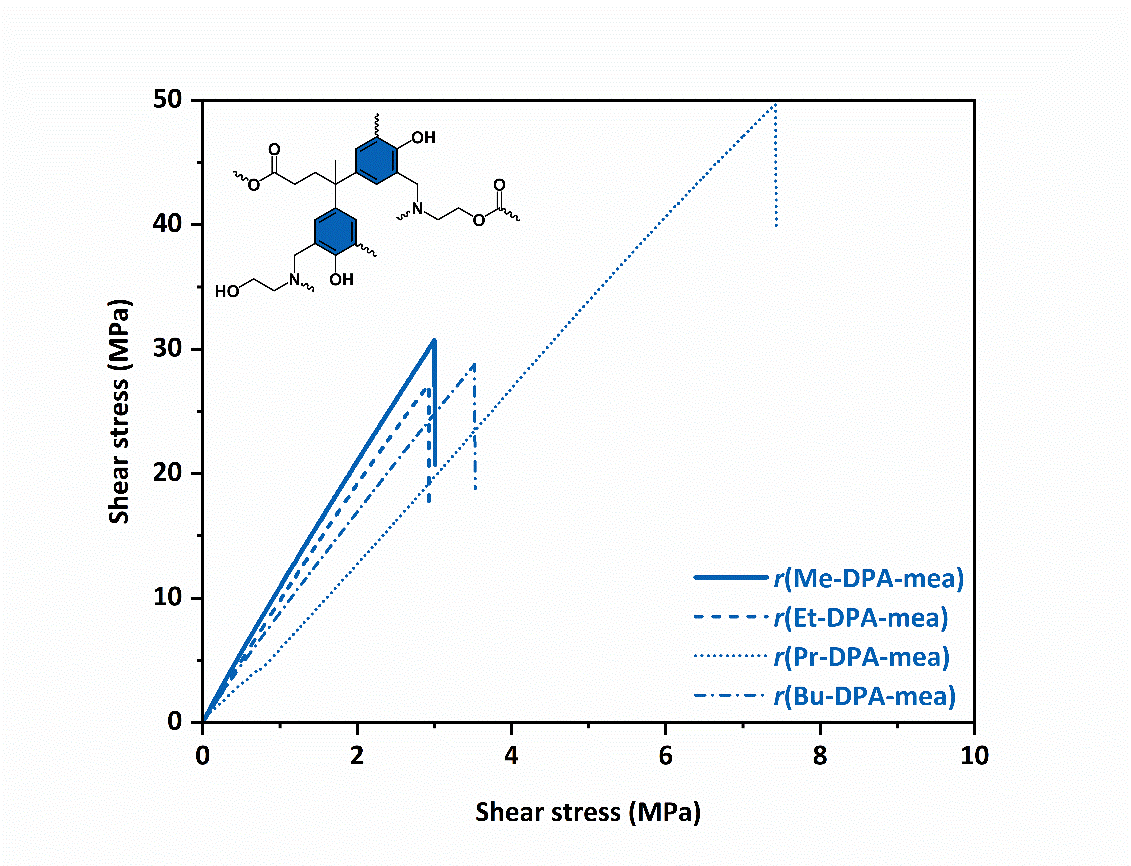


**Figure S48** Shear strain-shear stress curves of r(R-DPA-mea) reprocessed resins (0.5 %·min^-1^).


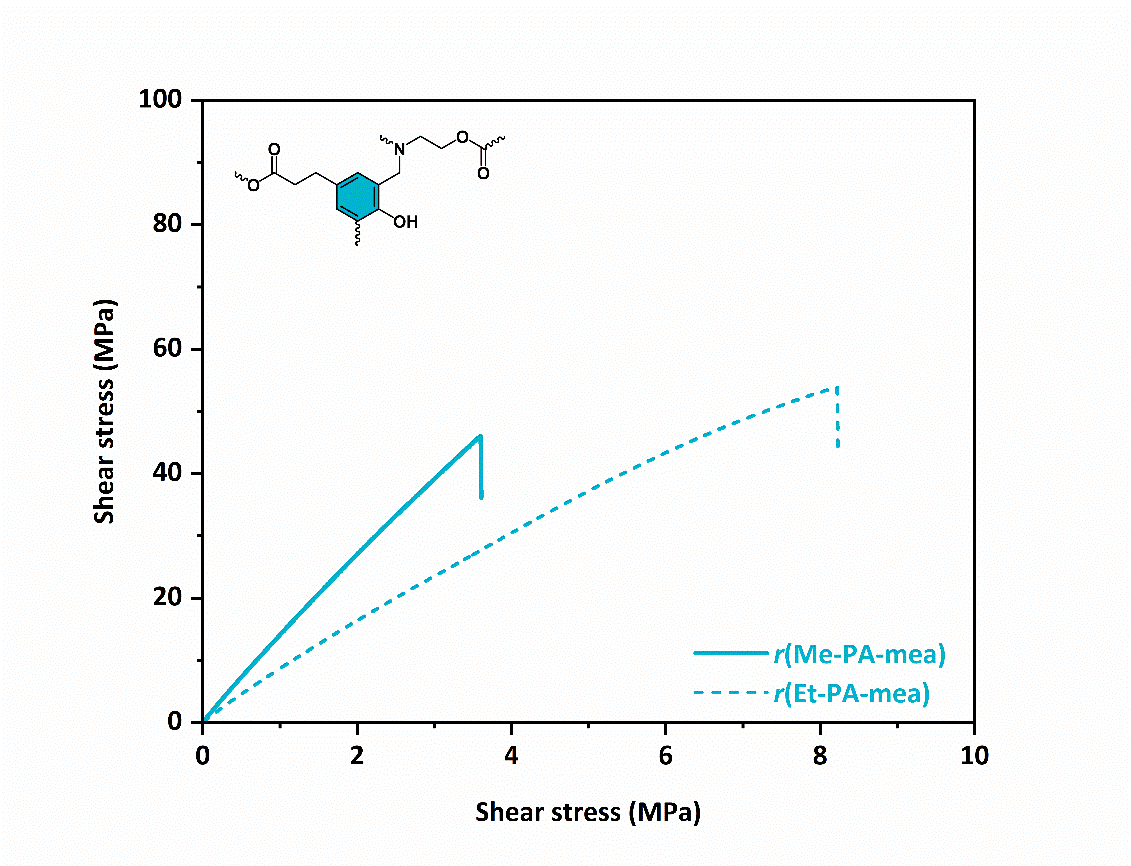


**Figure S47** Shear strain-shear stress curves of r(R-PA-mea) reprocessed resins (0.5 %·min^-1^).


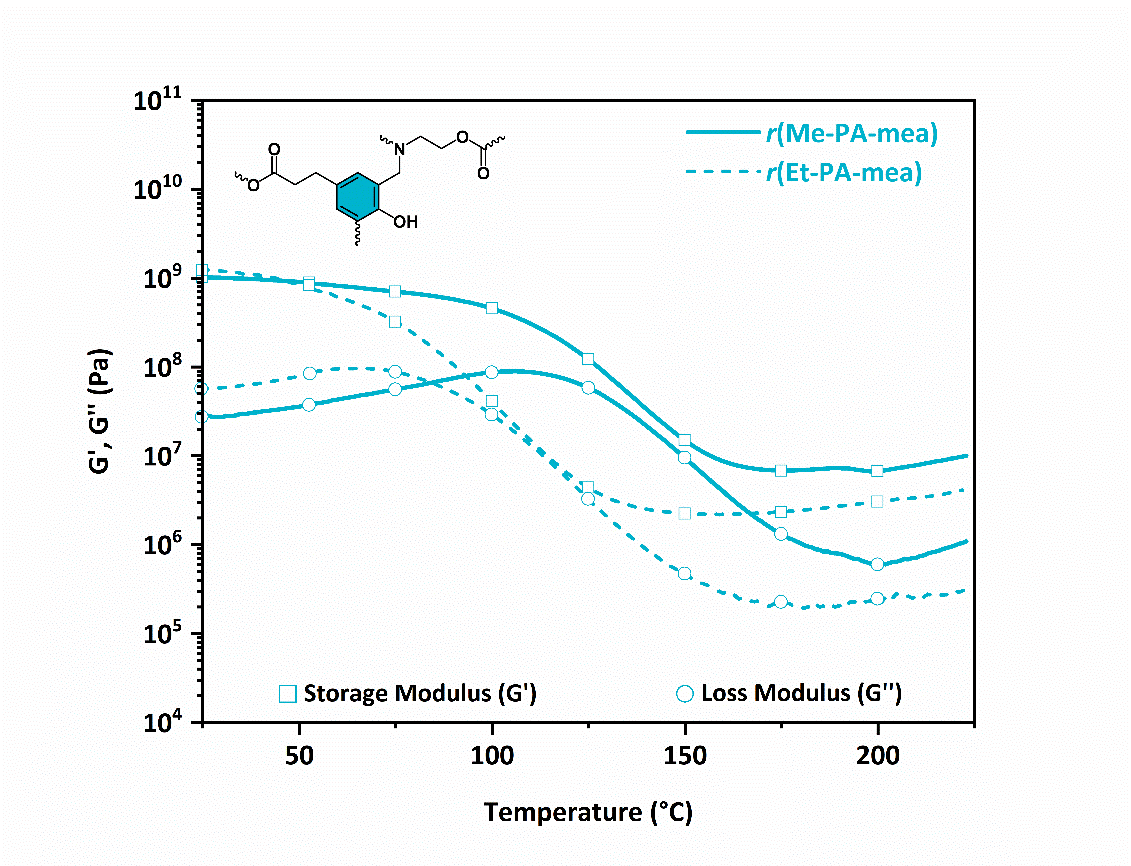


**Figure S49** Evolution of the storage and loss moduli of r(PA-mea) determined by rheology temperature sweep curve experiment. (f= 1 Hz, 2°C·min^-1^).


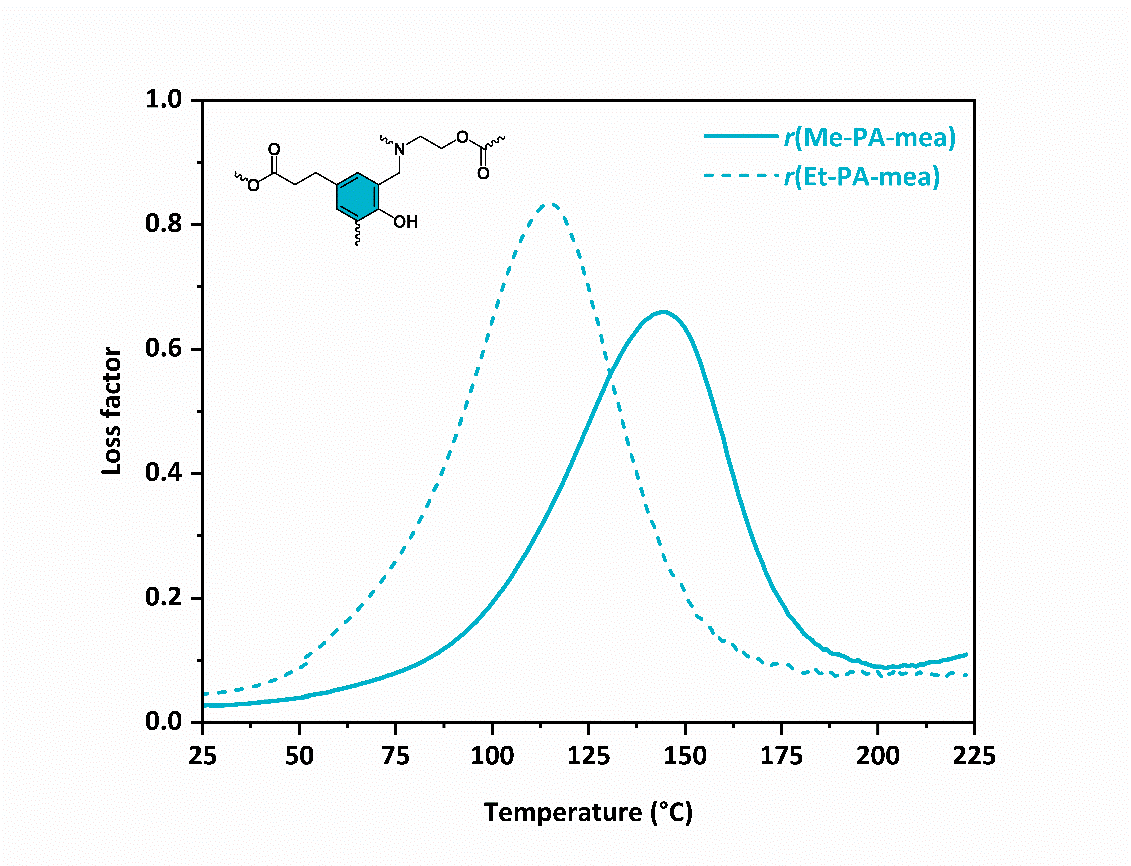


**Figure S50** Evolution of the loss factor of r(PA-mea) determined by rheology temperature sweep curve experiment. (f= 1 Hz, 2°C·min^-1^).


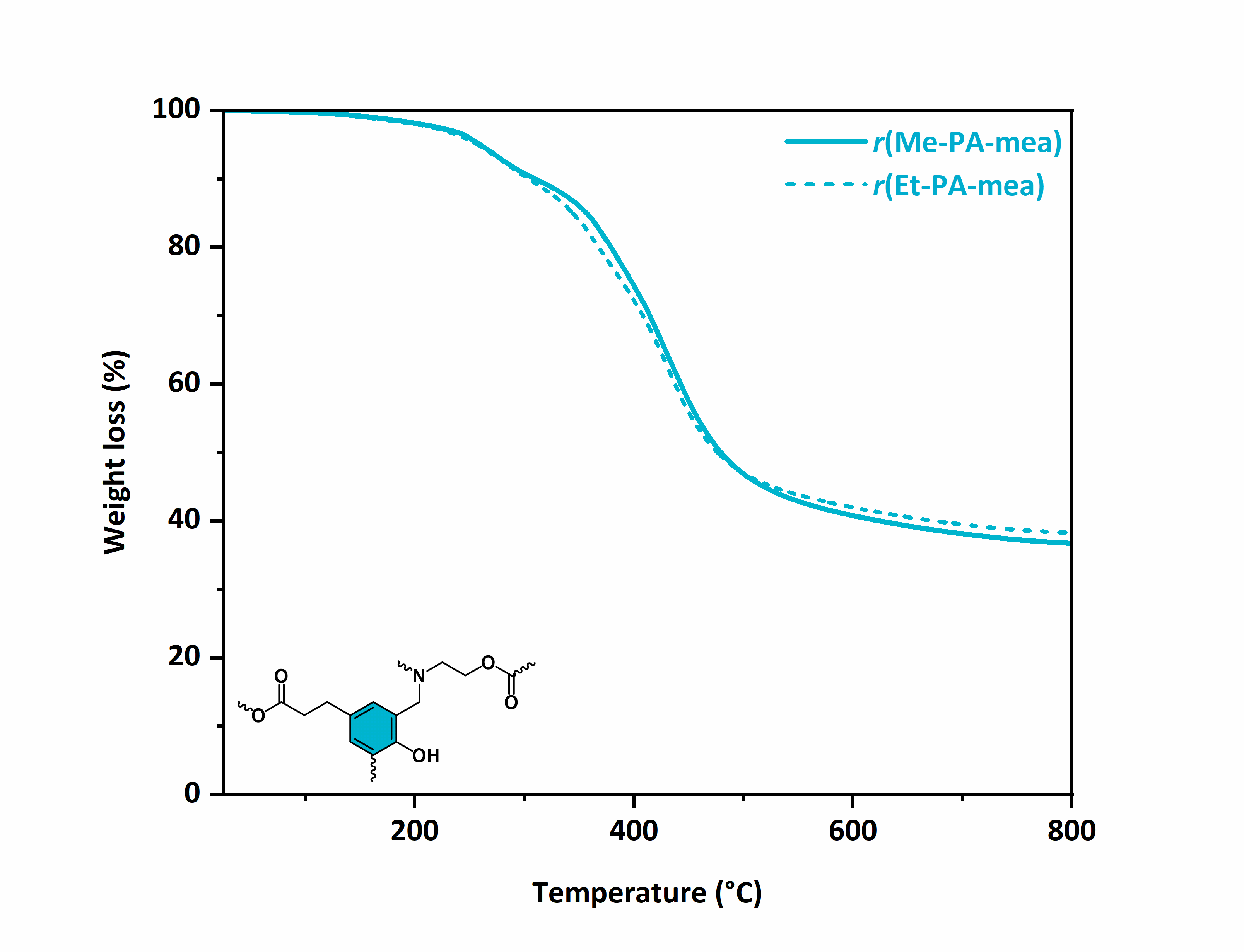


**Figure S52** Dynamic TGA curves of r(R-PA-mea) reprocessed polybenzoxazine resins (10°C·min^-1^, N_2_).


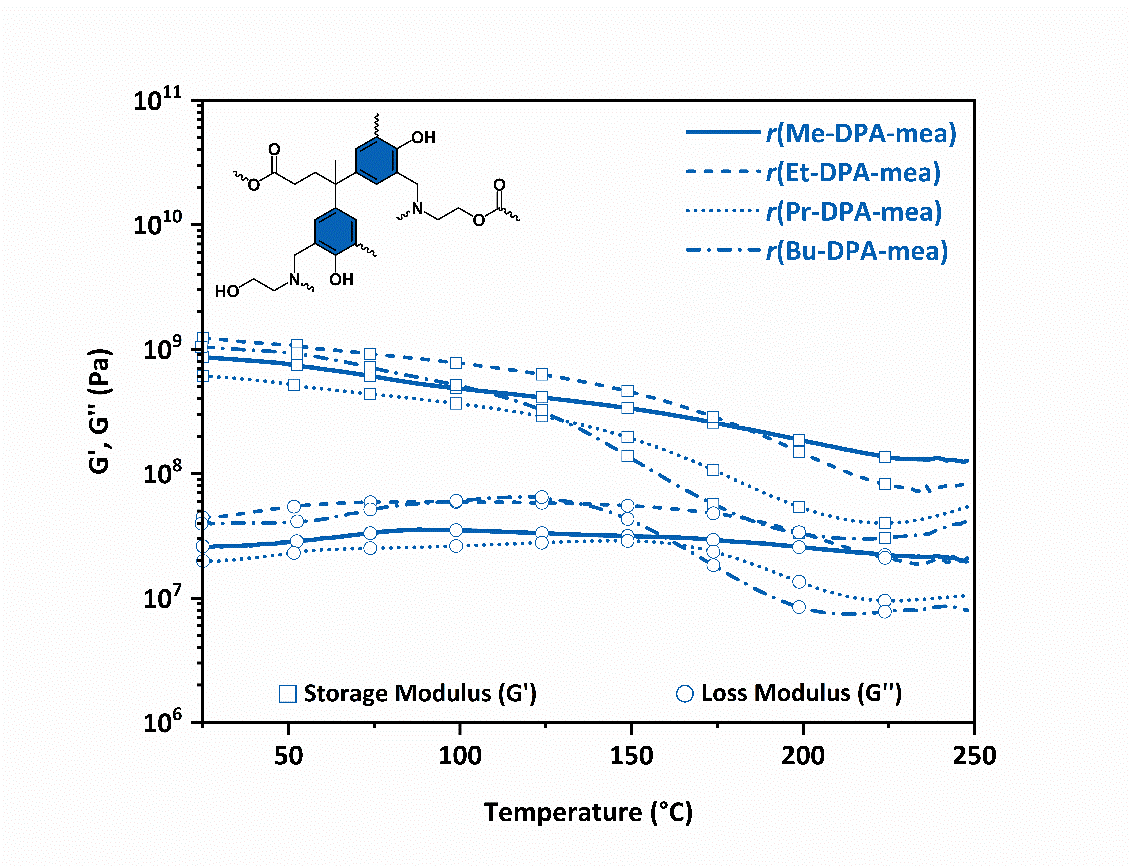


**Figure S51** Evolution of the storage and loss moduli of r(DPA-mea) determined by rheology temperature sweep curve experiment. (f= 1 Hz, 2°C·min^-1^).


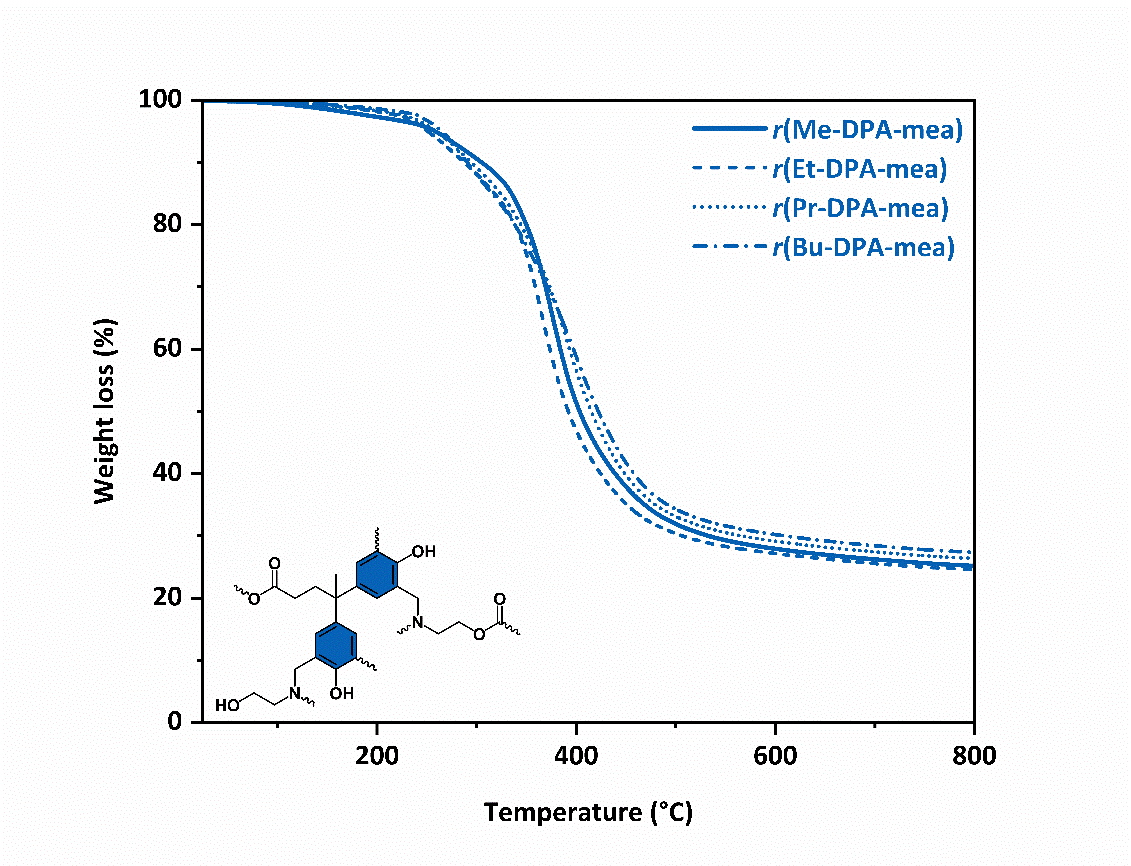


**Figure S53** Dynamic TGA curves of r(R-PA-mea) reprocessed polybenzoxazine resins (10°C·min^-1^, N_2_).

**Table S4** Cross-linking density and molecular weight between cross-links of reprocessed polybenzoxazine resins.

| Resin | ^a^ T_α_ (°C) | ^b^ G’ (MPa) | ^c^ Mc (g·mol^-1^) | ^d^ ν_e_ (mol·cm^-3^) |
| --- | --- | --- | --- | --- |
| r(Me-PA-mea) | 144 | 7.1 | 730 | 1860 |
| r(Et-PA-mea) | 114 | 2.2 | 2160 | 620 |
| r(Me-DPA-mea) | 232 | * 123.9 | 50 | 28640 |
| r(Et-DPA-mea) | 223 | ** 78.2 | 80 | 18220 |
| r(Pr-DPA-mea) | 196 | 43.7 | 110 | 10320 |
| r(Bu-DPA-mea) | 164 | 31.5 | 160 | 7930 |

^a^ α-mechanical relaxation (maximum of loss factor curve), ^b^ Storage modulus at the rubbery plateau (T_α_ + 40°C), ^c^ Molecular weight between crosslink determine according equation S5, ^d^ Cross-linking density determine according equation S6.

* Value taken at T_α_ + 10°C at the beginning of the rubbery plateau and before thermal degradation.
** Value taken at T_α_ + 20°C.
